# Modeling recapitulates the heterogeneous outcomes of SARS-CoV-2 infection and quantifies the differences in the innate immune and CD8 T-cell responses between patients experiencing mild and severe symptoms

**DOI:** 10.1371/journal.ppat.1010630

**Published:** 2022-06-27

**Authors:** Budhaditya Chatterjee, Harshbir Singh Sandhu, Narendra M. Dixit

**Affiliations:** 1 Centre for Biosystems Science and Engineering, Indian Institute of Science, Bangalore, India; 2 Department of Chemical Engineering, Indian Institute of Science, Bangalore, India; University of Leeds, UNITED KINGDOM

## Abstract

SARS-CoV-2 infection results in highly heterogeneous outcomes, from cure without symptoms to acute respiratory distress and death. Empirical evidence points to the prominent roles of innate immune and CD8 T-cell responses in determining the outcomes. However, how these immune arms act in concert to elicit the outcomes remains unclear. Here, we developed a mathematical model of within-host SARS-CoV-2 infection that incorporates the essential features of the innate immune and CD8 T-cell responses. Remarkably, by varying the strengths and timings of the two immune arms, the model recapitulated the entire spectrum of outcomes realized. Furthermore, model predictions offered plausible explanations of several confounding clinical observations, including the occurrence of multiple peaks in viral load, viral recrudescence after symptom loss, and prolonged viral positivity. We applied the model to analyze published datasets of longitudinal viral load measurements from patients exhibiting diverse outcomes. The model provided excellent fits to the data. The best-fit parameter estimates indicated a nearly 80-fold stronger innate immune response and an over 200-fold more sensitive CD8 T-cell response in patients with mild compared to severe infection. These estimates provide quantitative insights into the likely origins of the dramatic inter-patient variability in the outcomes of SARS-CoV-2 infection. The insights have implications for interventions aimed at preventing severe disease and for understanding the differences between viral variants.

## Author summary

Understanding the origins of the highly diverse outcomes of SARS-CoV-2 infection, ranging from clearance without symptoms to death, is an important challenge. Growing evidence points to the crucial roles of innate immune and CD8 T-cell responses in determining the outcomes. Delineating and quantifying the roles of these two immune arms would help better understand the origins of the diverse outcomes and inform intervention strategies aimed at preventing severe disease. Here, we developed a mathematical model of within-host SARS-CoV-2 dynamics and applied it to analyze clinical datasets comprising longitudinal viral load measurements from patients experiencing different severities of infection. Model predictions showed how a dynamical interplay between the strengths and the timings of innate and CD8 T-cell responses could recapitulate the diverse outcomes observed. Furthermore, the model provided excellent fits to the data and estimated that the innate immune and CD8 T-cell responses were nearly 80-fold and 200-fold stronger, respectively, in mildly versus severely infected patients. These estimates offer quantitative insights into the likely origins of the diverse outcomes of SARS-CoV-2 infection. We highlight potential implications for interventions and for understanding the differences between viral variants.

## Introduction

Infection by the severe acute respiratory syndrome coronavirus 2 (SARS-CoV-2) leads to remarkably heterogeneous clinical outcomes: Some individuals are cured without any symptoms, others experience mild to moderate symptoms, and yet others suffer severe disease, requiring hospitalization and intensive care, with a sizeable fraction of the latter suffering death [1–3]. While viral factors including mutations [4] may affect the outcomes, the heterogeneity in the outcomes has been observed from the early days of the pandemic [1, 3], before the different variants of SARS-CoV-2 emerged [5], suggesting that it potentially originates from the variability in host factors across individuals [6]. Indeed, several demographic correlates of disease severity, including gender, comorbidities, and age, have been identified [7]. The causes of the heterogeneous outcomes, however, are yet to be fully understood.

Accumulating evidence suggests that the variability in the immune responses across individuals, particularly innate and CD8 T-cell responses, may underlie the heterogeneous outcomes realized. Innate immune responses, involving type I and III interferons, are mounted soon after infection [8]. Patients with mild disease had higher levels of interferon responses early in infection in their upper respiratory airways than those with more severe disease [9,10]. A few days into the infection, the effector CD8 T-cell response is triggered and appears to play a critical role in the clearance of the infection [11]: The earlier the first detectable CD8 T-cell response, the shorter is the duration of the infection [12]. CD8 T-cell numbers were higher in the bronchoalveolar lavage fluids of individuals with mild/moderate symptoms than in those with severe infection [13]. Clonal expansion of CD8 T-cells was compromised in patients with severe symptoms [13,14]. The severity of the symptoms was also proportional to the level of exhaustion of CD8 T-cells [15,16].

If the disease is resolved in time, typically in 2–3 weeks, the cytokines and activated CD8 T-cell populations decline and eventually fade away, leaving behind memory CD8 T-cells [11]. If the disease is not resolved in a timely manner, uncontrolled cytokine secretion may result, triggering immunopathology and severe disease [6]. Elevated interferon responses were detected in severely infected and deceased patients late in infection [9,17,18], with the lung suffering the most damage [19]. With prolonged disease, where viral load could be detected in patients over extended durations–up to 66 days on average in some cohorts [20–22]–proliferation and differentiation of CD8 T-cells were compromised [20].

Other arms of the immune system appear to contribute much less to the clearance of the infection. Antibodies arise late, a couple of weeks into the infection [11,23], and, while important in vaccine-mediated protection [24–26], appear to play a minor role in clearing the infection in unvaccinated individuals [11]. Antibody titers are higher in severely infected than in mildly infected individuals, suggesting that antibody production trails virus growth [11]. Whereas a subset of antibodies may aid viral clearance [27], autoantibodies, targeting cytokines and cell surface and structural proteins of the host, may worsen disease [28]. Innate immune cells, such as neutrophils, macrophages, and natural killer cells, too are thought not to contribute significantly to clearance, but may nonetheless enhance immunopathology [6,29].

Taken together, current evidence points to the crucial roles of the innate immune and CD8 T-cell responses in determining the outcomes of the infection. Delineating and quantifying their roles would help better understand the origins of the heterogeneous outcomes and have implications for interventions. Here, we developed a mathematical model of within-host SARS-CoV-2 dynamics that incorporates the key features of the innate and the CD8 T-cell responses, tested its ability to recapitulate the heterogeneous outcomes realized, and employed it to analyze multiple patient datasets, representative of the heterogeneous outcomes.

## Results

### Mathematical model of within-host SARS-CoV-2 dynamics

**Fig 1:**
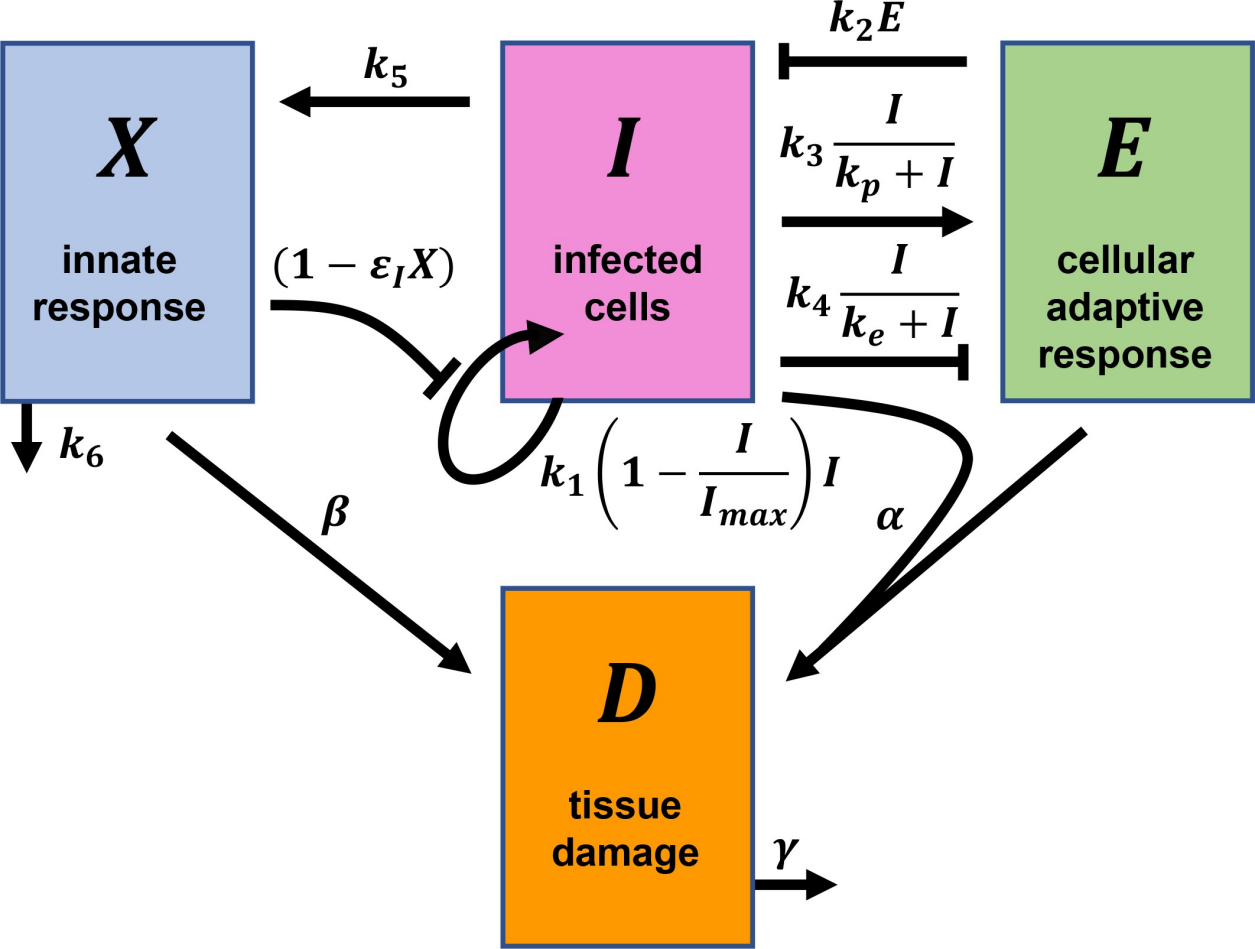
Schematic of the mathematical model of within-host SARS-CoV-2 infection. The key quantities and their interactions contained in our model (Eqs 1–4) are illustrated. Arrows and blunt-head arrows depict positive and negative regulation, respectively. The parameters and rate expressions shown next to the arrows are described in the text.

We modeled disease progression in an individual infected by SARS-CoV-2 by following the time-evolution of the population of infected cells (*I*), the strength of the effector CD8 T-cell response (*E*), the strength of the cytokine-mediated innate immune response (*X*), and tissue damage (*D*) (Fig 1). Following previous studies [30–32], we considered the essential interactions between these entities and constructed the following equations to describe their time-evolution:

$$\frac{dI}{dt}=k_{1}(1-\varepsilon_{I}X)I\left(1-\frac{I}{I_{max}}\right)-k_{2}IE$$ (1)

$$\frac{dE}{dt}=k_{3}\left(\frac{IE}{k_{p}+I}\right)-k_{4}\left(\frac{IE}{k_{e}+I}\right)$$ (2)

$$\frac{dX}{dt}=k_{5}I-k_{6}X$$ (3)

$$\frac{dD}{dt}=\alpha IE+\beta X-\gamma D$$ (4)

Here, the infected cell population follows logistic growth [30] with the per capita growth rate *k*_1_ and carrying capacity *I*_*max*_. (The list of all model parameters is in Table 1.) This growth arises from the infection of target cells by virions produced by infected cells [30]. *I*_*max*_ is the maximum number of cells that can get infected, due to target cell or other limitations. The growth rate *k*_1_ is assumed to be reduced by the innate immune response, *X*, with the efficacy *ε*_*I*_*X*, due to interferon-mediated protection of target cells and/or lowering of viral production from infected cells [8]. Effector cell-mediated killing of infected cells is captured by a mass action term with the second-order rate constant *k*_2_. The proliferation and exhaustion of CD8 T-cells are both triggered by infected cells at maximal per capita rates *k*_3_ and *k*_4_, respectively. *k*_*p*_ and *k*_*e*_ are the levels of infected cells at which the proliferation and exhaustion rates are half-maximal, respectively. Following previous studies, we let *k*_3_<*k*_4_ and *k*_*p*_<*k*_*e*_, so that proliferation dominates at low antigen levels and exhaustion at high antigen levels [30,31,33], consistent with the delayed onset of exhaustion relative to proliferation [34]. Alternative forms have been employed to describe exhaustion, which allow cumulative antigenic stimulation to trigger exhaustion, but have been shown to yield similar outcomes to the present form [30,35]. We explore these alternative forms below. The innate response, *X*, is triggered by infected cells at the per capita rate *k*_5_ and is depleted with the first-order rate constant *k*_6_.

**Table 1:** Model parameters and their description.

Parameter	Description	Units
*k*_1_	Per capita growth rate of infected cells	day^-1^
*k*_2_	Second-order rate constant for effector cell-mediated killing of infected cells	cells^-1^ day^-1^
*k*_3_	Per capita proliferation rate of CD8 T-cells	day^-1^
*k*_4_	Per capita exhaustion rate of CD8 T-cells	day^-1^
*k*_5_	Per capita triggering rate of innate immune response by infected cells	cells^-1^ day ^-1^
*k*_6_	Per capita depletion rate of innate immune response	day^-1^
*ε*_*I*_	Per capita reduction in infected cell growth rate by the innate immune response	dimensionless
*I*_*max*_	Carrying capacity of infected cells	cells
*k*_*p*_	Half maximal constant for proliferation rate of effector cells	cells
*k*_*e*_	Half maximal constant for exhaustion rate of effector cells	cells
*α*	Rate of tissue damage caused by CD8 cells	cells^-1^ day ^-1^
*β*	Rate of tissue damage caused by innate immune response	day^-1^
*γ*	Rate of recovery of the damaged tissue	day^-1^
*τ*	Viral incubation period	day
*ζ*	Time period from the start of viral growth to symptom onset	day

To assess the severity of infection, we employed *D*, which represents the instantaneous tissue damage, with contributions from CD8 T-cell mediated killing of infected cells, determined by *αIE*, and from proinflammatory cytokines, represented by *βX*. Inflamed tissue is assumed to recover with the first order rate constant *γ*. Using *D*, we quantified the extent of immunopathology, *P*, as follows. In our model, *D* typically rose as the infection progressed and declined as it got resolved (see below). We reasoned that the severity of infection would be determined by the maximum tissue damage suffered as well as the duration of such damage. Significant damage that is short-lived or minimal damage that is long-lived may both be tolerable and lead to mild symptoms. We, therefore, calculated the area under the curve (AUC) of *D* as a measure of immunopathology. To aid comparison across individuals, we set the scale for immunopathology by the AUC of *D* calculated using the population parameters for mildly infected individuals (see below), starting from when *D* ascended above its half-maximal level to the time when it descended below that level (S1 Fig). For any individual, we computed the AUC of *D* between the same threshold levels (half-maximal levels corresponding to the population parameters). We reported the ratio of the AUC of the individual to that of the population parameters as a measure of relative immunopathology, *P*, of the individual and the associated disease severity. *P*>1 would thus imply more severe disease than the typical mildly infected individual, whereas *P*<1 would indicate less severe disease. We explored alternative ways of estimating *P* from the predictions of *D* and found that they all yielded similar qualitative conclusions (S1 Text and S2 Fig).

Eqs 1–4 along with the above formalism for estimating immunopathology presented a model of within-host SARS-CoV-2 dynamics that incorporated the essential features of innate immune and CD8-T cell responses. To test whether the model was representative of the dynamics *in vivo* and to estimate model parameters, we fit the model to patient data.

### Model was consistent with *in vivo* dynamics

To test our model, we sought datasets that included accurate estimates of the time of contracting the disease because the initial phases of the immune response were likely to be important in determining disease outcome; in asymptomatic individuals, this early response clears the infection [36]. We found such data in a study of one of the first SARS-CoV-2 transmission chains in Germany in early 2020 [37,38]. The study traced the dates of first exposure to the virus of each patient in the transmission chain [37] (S2 Text and S1 Table). Further, daily viral load data, measured in nasopharyngeal swab and sputum samples, from all patients starting from the onset of symptoms or earlier were reported [38]. We employed data from the sputum samples first. We considered data from day zero to day 15 of the infection (S2 Text and S1–S3 Tables) to avoid any possible confounding effects from the humoral response, which is mounted after 2 weeks in most patients [11,23].

**Fig 2:**
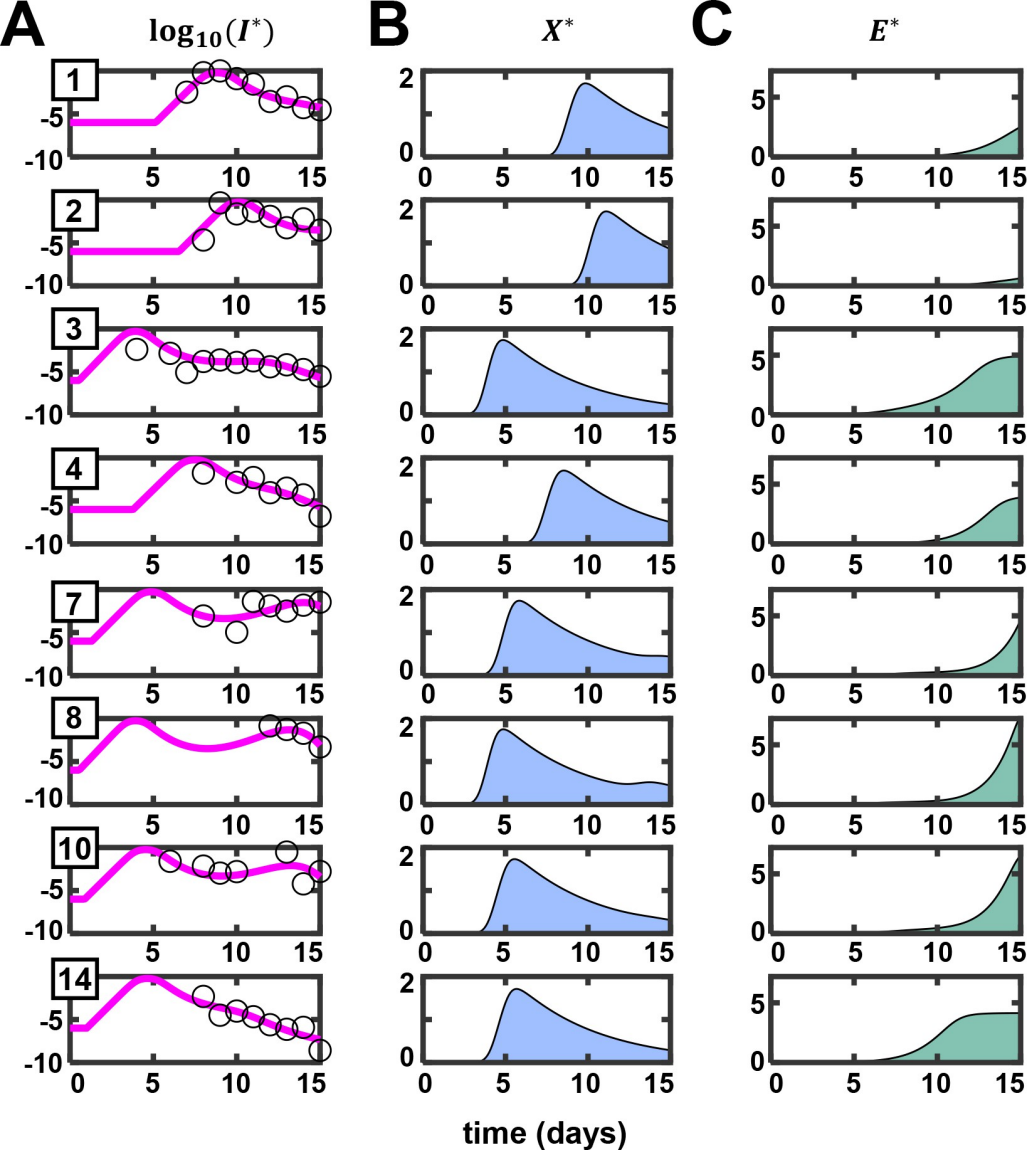
Model predictions are consistent with *in vivo* dynamics. (A) Best-fits of our model (lines) to patient data (symbols) of normalized sputum viral load as a function of time from viral exposure [38]. The corresponding (B) innate immune responses and (C) CD8 T-cell responses predicted by our model. The asterisks represent rescaled variables (see Methods). Patient IDs, as provided in Bӧhmer et al. [37], are in the top-left corner of each subplot in (A).

All patients in this dataset had mild symptoms, which waned by day 7 after the first virological test. The patients were of working age and otherwise healthy. In such patients, markers of T-cell exhaustion are not significantly higher than healthy individuals and are markedly lower than severely infected patients [15]. Therefore, to facilitate more robust parameter estimation, we ignored CD8 T-cell exhaustion in the present fits (by fixing *k*_4_ = 0). Furthermore, we assumed that the viral population, *V*, is in a pseudo-steady state with the infected cell population, so that *V*∝*I*. The assumption is supported by the large burst size of SARS-CoV-2 (~10^5^ virions/cell) [39], which is comparable to that of HIV [40] and much larger than influenza (~10^3^ virions/cell) [41]. Because the dynamics of tissue damage (*D*) is dependent on but does not affect disease dynamics in our model, we ignored *D* for the present fitting. This was further justified because the patients considered for fitting were mildly/moderately infected and were expected to have suffered minimal tissue damage. Because the patients were all similar, we assumed that *I*_*max*_ would be similar in them and proportional to *V*_*max*_, the highest viral load reported across the patients. We thus fit log_10_(*I*/*I*_*max*_), i.e., log_10_(I*), calculated with our model to the normalized data of log_10_(*V*/*V*_*max*_) (Methods). Our fits were not sensitive to *I*_*max*_; best fit parameter estimates were similar and/or had overlapping error ranges (S4A and S4B Table). We allowed a delay following exposure to account for the incubation period before viral replication can begin. This delay, denoted *τ*, was introduced using Heaviside functions in our model (see Eqs 5–7 in Methods). We used a nonlinear mixed-effects modeling approach for parameter estimation [42]. Our model provided good fits to the data (Fig 2A) and yielded estimates of the parameters at the population-level (Table 2) and for the individual patients (Table 3). Visual predictive check and shrinkage of parameters estimated indicated the reliability of our fits (S3 Fig). The fits indicated that our model was consistent with the dynamics *in vivo*. We quantified the uncertainties in our individual patient fits and parameter estimates using multiple realizations of the predictions with parameter combinations sampled from distributions conditioned on the individual patient data (S4 Fig and S5 Table).

**Table 2:** Estimated population parameters of the model fit to the sputum viral load dataset [38] (Fig 2).

Parameter	Unit	Fixed effect (SD)		Random effect (SD)	
***k***_**1**_	day^-1^	4.49	(0.20)	0.28	(0.14)
***k***_**3**_ ×10^1^	day^-1^	7.4	(1.19)	0.05	(0.17)
$k_{5}^{*}$	day^-1^	2.83	(0.83)	0.32	(0.31)
$E_{0}^{*}$ ×10^3^	^§^day^-1^	6.65	(5.97)	0.25	(0.99)
$k_{p}^{*}$ ×10^6^	dimensionless	249.67	(284.22)	2.37	(0.86)
***τ***	day	1.51	(0.60)	1.22	(0.25)

^§^ E* = *k*_2_. *E* (see Methods).

**Table 3:** Estimated individual parameters of the model fit to the sputum viral load dataset [38] (Fig 2). Units are the same as in Table 2.

Patient ID	*k*_1_	*k*_3_×10^1^	$k_{5}^{*}$	$E_{0}^{*}\times 10^{3}$	$k_{p}^{*}\times 10^{6}$	*τ*
**1**	4.43	7.43	2.46	6.91	79.65	5.14
**2**	4.44	7.36	2.93	6.57	320.76	6.54
**3**	4.65	7.45	3.34	7.16	130.39	0.53
**4**	4.37	7.38	2.49	6.57	35.05	3.78
**7**	4.48	7.33	2.99	6.51	1216.12	1.31
**8**	4.72	7.35	3.04	6.42	1078.61	0.55
**10**	4.41	7.34	2.64	6.42	893.37	0.88
**14**	4.45	7.35	2.53	6.39	30.38	1.00

To ascertain the robustness of our model and fits, we tested several variants of our model. We fit variants without the adaptive response; without the innate response; with a logistic growth formulation of the innate immune response; with the innate response amplifying the adaptive response; or combinations thereof; to the same data (S3 Text and S6 Table). The fits were all poorer than the present model as indicated by the AICc and BICc values (Fig 2 and S7 Table). We also examined a model that allowed effector cell proliferation to depend on the rate of antigen increase and found it to be structurally similar to the present model (S3 Text). We therefore employed the present model for further analysis.

### Model elucidated plausible origins of distinct patterns of viral clearance

The best-fits above yielded important insights into the underlying dynamics of disease progression and clearance. First, our model offered a plausible explanation of the two distinct patterns of clearance observed in the patients. Patients 1, 2, 4, and 14 had a single peak in their viral load data followed by a decline leading to clearance (Fig 2A, open circles). Patients 7 and 10, in contrast, had a second peak following the first before clearance. The origins of these multiple peaks have been elusive [43]. For patients 7, 8, and 10, our best-fits predicted an early innate immune response and a delayed CD8 T-cell response (Fig 2B and 2C). The second peak was thus likely to arise from the interactions between the virus and the innate immune response, before the CD8 T-cell response was mounted. To test this, we examined model predictions in the absence of the CD8 T-cell response.

**Fig 3:**
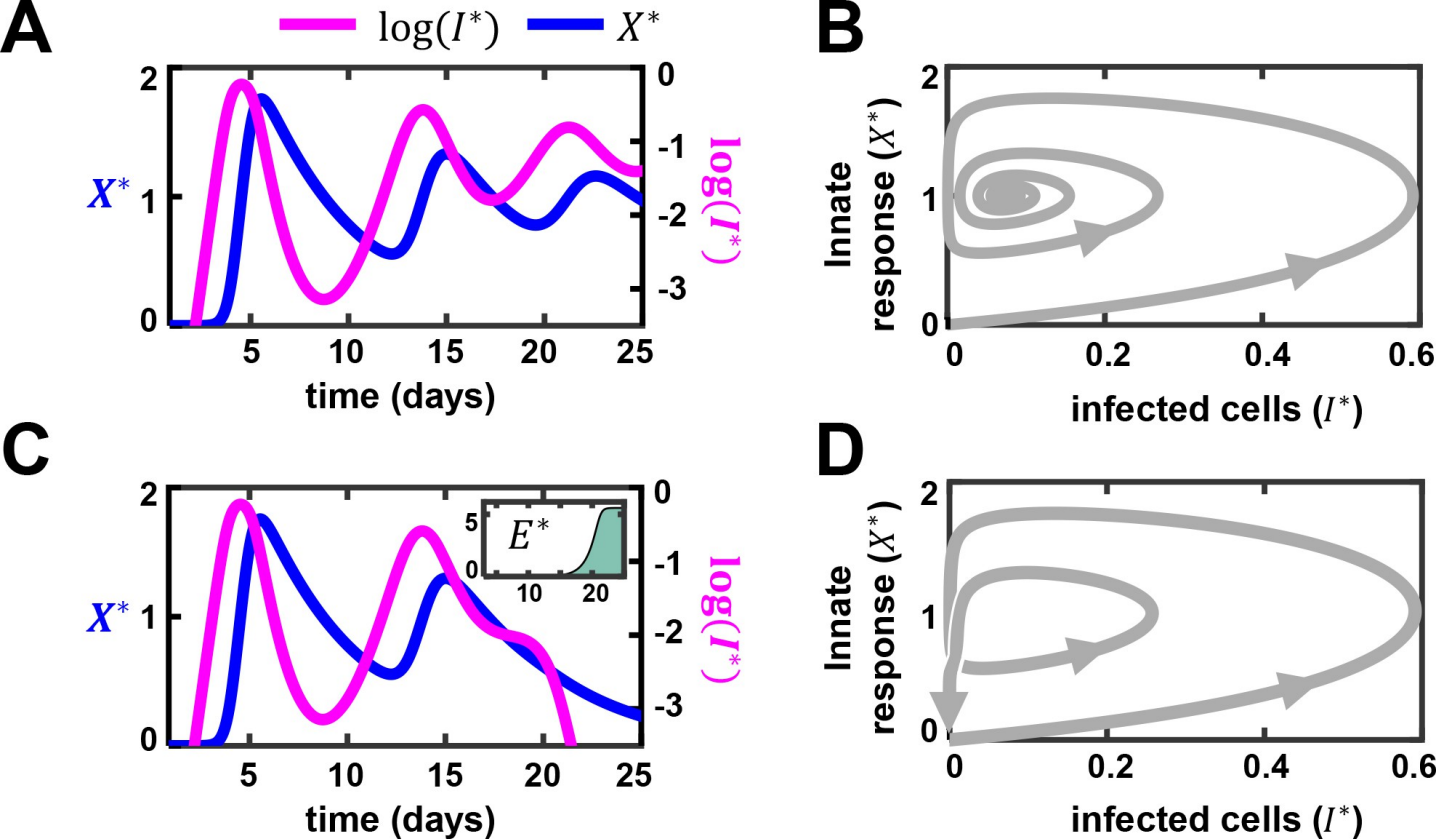
Predator-prey-like oscillations may underlie multiple viral load peaks. (A) The dynamics of infected cells (*I*) and innate immune response (*X*) in the absence of CD8 T-cells (*E*). The asterisk represents rescaled variables (see Methods). (B) Corresponding trajectory on a phase plane plot of infected cells and innate response. (C) and (D) Predictions as in (A) and (B) but in the presence of *E*. Inset shows the dynamics of *E* over time (in days). The individual parameters for patient id 10 (Table 3) were used. Parameter values used are as the following: *k*_1_ = 4.41/day, *k*_3_ = 0.73/day, $k_{5}^{*}=2.64$/day, $k_{p}^{*}=8.93\times 10^{-4},$ *τ* = 0.88 day, *k*_6_ = 0.2/day, *k*_4_ = 1.5/day, $k_{e}^{*}=0.7,$ *α* = 1.0×10^4^, *β* = 2.0×10^4^/day, *γ* = 0.5/day. In (A) and (B), we set $E_{0}^{*}=0$ whereas in (C) and (D), we let $E_{0}^{*}$ = 6.42×10^−3^/day.

In our model, the innate immune response, *X*, and infected cells, *I*, showed signatures of the classic predator-prey interactions [44], with *I* the prey and *X* the predator: *I* grows in the absence of *X*, whereas *X* declines in the absence of *I*. *I* triggers the growth of *X*, which in turn suppresses *I*. These interactions, as with the predator-prey system [44,45], result in oscillatory dynamics (Fig 3A). Thus, following infection, *I* grows, causing a rise of *X* in its wake. When *X* rises sufficiently, it suppresses *I*. When *I* declines substantially, the production of *X* is diminished and *X* declines. This allows *I* to rise again, and the cycle repeats. For the parameter values chosen, the oscillations were damped and settled to a persistent infection state with non-zero *I* and *X* (Fig 3B). Using stability analysis, we found that clearance was not a stable steady state of the system (S4 Text). Thus, viral clearance was not possible in our model without the CD8 T-cell response (*E*).

We next reintroduced CD8 T-cells in our simulations (Fig 3C and 3D). Our results indicated that CD8 T-cells broke the oscillatory predator-prey cycles and facilitated clearance. When *E* rises, it can suppress *I* independently of *X*. By lowering *I*, it creates the opportunity for *X* to dominate *I*. Together, *X* and *E* can then clear the infection (Fig 3C and 3D). Note that previous modeling studies have shown that CD8 T-cells alone can drive viral clearance, but the associated immunopathology (or disease severity) may depend on the innate immune response [30].

It followed from the above analysis that the second peak in viremia seen in patients 7, 8 and 10 was likely to be a manifestation of the underlying predator-prey oscillations that occurred before the CD8 T-cell response was mounted. Indeed, when we fit the data in the absence of an effector response (*E* = 0), the fits were similar until the late stages of infection, when the effector response is expected to be mounted, and yielded prolonged predator-prey like oscillations (S5 Fig). (We note that values of X*>1 imply that the innate immune response not only prevents new infections but also reduces the population of infected cells, which could occur either by the triggering of inflammatory cell death [46] or by driving infected cells to an antiviral state [47].) In patients 3, 4, and 14, a relatively early CD8 T-cell response was predicted, which precluded the second peak. In patients 1 and 2, both the innate and CD8 T-cell responses were delayed, leaving little time for the oscillations to arise in the 15 day period of our study.

Second, the post-exposure delay in viral replication varied from *τ* = 0.6 d to 6.5 d in the patients analyzed (Table 3), with a mean±SEM of 2.5±0.8 days, reflecting the variability in the time of the establishment of systemic infection following exposure, and consistent with the variable prodromal period observed [48]. (Note that the mean mentioned is of the individual patient parameters in Table 3 and is thus different from the population mean in Table 2.) The initial, possibly stochastic [49], events during the establishment of infection might be associated with the variability in the delay in viral replication. Third, the transient but robust innate immune response predicted (Fig 2B) is consistent with observations in mildly/moderately infected patients [50]. In the latter study [50], the type I interferon level was elevated early in moderately infected patients compared to severely infected patients and was also resolved sooner. Fourth, the prediction of the dynamics of the CD8 T-cell response, where a gradual build-up is followed by a stationary phase (Fig 2C), is also consistent with observations: In mildly infected patients, SARS-CoV-2 specific T-cells were detected as early as 2–5 days post symptom onset [12]. This effector population remained stable or increased for several months after clinical recovery [51,52].

Our model thus fit the dynamics of infection in individuals and offered explanations of disease progression patterns that had remained confounding. This gave us confidence in our model. We applied it next to assess whether the variability in innate and CD8 T-cell responses could capture the heterogeneity of the outcomes realized.

### The interplay between innate and CD8 T-cell responses can explain the heterogeneous outcomes

**Fig 4:**
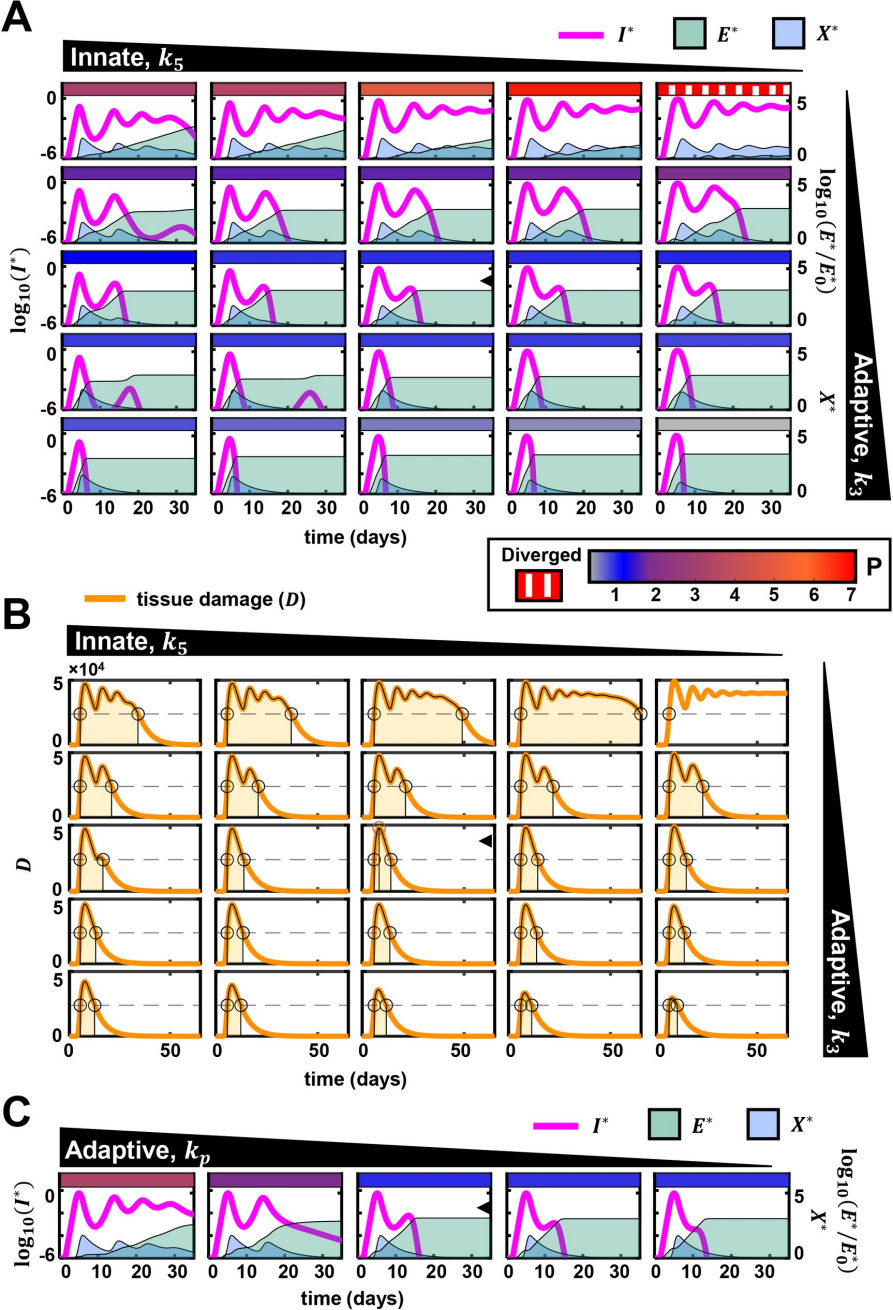
Variations in innate and CD8 T-cell responses capture disease heterogeneity. (A) Effect of variation of parameters determining the strengths of innate and CD8 T cell responses on the trajectory of the infection. The black annotated triangles at the top and right indicate the nature and the direction of the variation of the indicated parameters. Individual subplots show the dynamics of infected cells, cytokine mediated innate immune response, and effector CD8 T-cell response. In each subplot, the left Y-axis shows the normalized infected cell dynamics and the right Y-axis shows the other two species. The rectangular, colored patch at the top of each subplot represents the extent of immunopathology. The range of immunopathology is given by the color scale at the bottom. On the left-side of the color scale, a separate legend denotes the texture used for depicting unbounded immunopathology. Unity on the colorscale indicates the immunopathology quantified in the central subplot (subplot with an arrowhead), calculated using the population parameters estimated from Fig 2. (B) The tissue damage (*D*) associated with each subplot in (A) is shown. The area shaded light orange in each panel is used to calculate immunopathology (see S1 Fig), and is also depicted by the colored patches in the subplots of (A). (C) The effect of varying the sensitivity of CD8 T-cell response to antigen, *k*_*p*_. The presentation is similar to (A). The scale for immunopathology is in (A). The population estimates (fixed effects) of the parameters estimated in Table 2 were used. Other parameter values used are: *k*_6_ = 0.2/day, *k*_4_ = 1.5/day, $k_{e}^{*}=0.7,$ *α* = 10^4^, *β* = 2.0×10^4^/day, *γ* = 0.5/day. Variations in *k*_3_ are obtained as the following fold-changes to the above value: 0.35, 0.75, 1, 2, 3. The fold-changes for variation in $k_{5}^{*}$ are: 0.5, 0.75, 1, 2, 5. Values of $k_{p}^{*}$ used in (C) are: 1.0×10^−5^, 1.0×10^−4^, 2.497×10^−4^, 5.0×10^−3^, 5.0×10^−2^.

To delineate the roles of the innate and CD8 T-cell responses in determining the outcomes, we performed a comprehensive scan of the parameter space, spanning wide ranges of the strengths and timings of the two immune arms. We present the dynamics of *I*, *E*, *X* and *D*, and hence *P*, over a range of values of *k*_3_ and *k*_5_ (Fig 4). We recall that *k*_3_ is the proliferation rate constant of CD8 T-cells and *k*_5_ is the growth rate constant of the innate immune response. The other parameters were held constant unless indicated otherwise. When both *k*_3_ and *k*_5_ were high, indicating strong innate and CD8 T-cell responses, *I* rose following the infection, attained a peak, and then declined to low levels and vanished, marking rapid clearance of the infection (Fig 4A, bottom left). *X* correspondingly rose and declined following the rise and fall of *I*. *E* too rose swiftly following the infection and remained high after the infection was cleared, mimicking the existence of effector cells well past the clearance of infection [51,52]. (In our model, an explicit decay of CD8 T-cells is not incorporated for simplicity [30].) These overall dynamics are representative of mild or asymptomatic infections.

Decreasing *k*_5_ weakened the innate response and resulted in an increase in the peak of infected cells (Fig 4A, bottom row, left to right). The slower induction of the innate response allowed an increased number of infected cells to accumulate (S6A and S6B Fig). Peak viral load thus rose. The latter trends have parallels to infected patients with impaired innate responses, such as those harboring mutations in the genes associated with the activation of the antiviral resistance in host cells [53]. Clearance was still achieved without substantial variation in the infection duration and with limited immunopathology because of a strong CD8 T-cell response. This behavior is consistent with observations where an early and robust effector T-cell response has been associated with mild infections [12,51,52].

Decreasing *k*_3_ weakened and delayed the CD8 T-cell response and increased the duration of the infection (Fig 4A, left column, bottom to top). Furthermore, with a decrease in *k*_3_, the duration of tissue damage, *D*, increased, increasing immunopathology, *P* (Fig 4A and 4B, left columns, bottom to top). When *k*_3_ was low and *k*_5_ was high (Fig 4A, four subplots at the top-left), the efficient innate response controlled the initial peak of the infection. However, the slow proliferation of the effector cells delayed clearance. This scenario has parallels to the reported cases of prolonged RT-PCR positivity [20–22]. Restrained CD8 T-cell differentiation was associated with such cases [20]. Delayed clearance was also realized when the parameter *k*_*p*_ was increased, which increased the threshold antigen level required for significant effector CD8 T-cell proliferation (Fig 4C). These latter predictions were consistent with observations of defects in T-cell proliferation delaying the clearance of infection [21].

CD8 T-cell responses could also be weakened by exhaustion. Indeed, exhausted CD8 T-cells were associated with prolonged infection in some patients [54]. Interestingly, low proinflammatory cytokine and monocyte levels and high regulatory T cell levels appeared to limit immunopathology in the latter cohort [54]. In our model, a higher rate of T-cell exhaustion (increasing *k*_4_ and/or decreasing *k*_*e*_) and a weaker innate response (increasing *k*_5_) together resulted in prolonged infection (S7A and S7B Fig). Further, a lower rate of cytokine-mediated tissue damage (*β*) limited immunopathology despite prolonged SARS-CoV-2 positivity, recapitulating the latter clinical observations (S8A and S8B Fig).

When both *k*_3_ and *k*_5_ were low, indicating weak innate and CD8 T-cell response, (Fig 4A, four subplots at the top-right), our model predicted severe immunopathology along with prolonged infection with high viral load and high cytokine levels. When *k*_3_ and *k*_5_ were the lowest in the ranges we considered, clearance was not achieved in our predictions. To understand this outcome, we performed a detailed dynamical systems analysis of our model (S5 Text and S9 and S10 Figs). Although clearance was the predominant outcome and was associated with a wide range of parameter values (Fig 4), parameter regimes could exist where clearance was not realized and the infection could persist long-term in our model (S5 Text and S9 and S10 Figs). Note that long-term persistence has been recognized as an alternative outcome of such dynamical systems associated with different viral infections [30–32,55]. In our present predictions, trajectories leading to persistence were typically associated with high cytokine and infected cell levels and high levels of CD8 T-cell exhaustion and resulted in excessive immunopathology (Fig 4A and 4B, top right corner). Such trajectories were likely to be terminated prematurely by fatality [56]. These trends in the model mirrored clinical features of severe COVID-19 [50], which include consistently high viral loads, heightened proinflammatory cytokines and interferons [50,56,57], and attenuated proliferation [13] and increased exhaustion of T-cells [13,14,16].

The initial pool of CD8 T-cells, *E*_0_, was important in determining outcomes (S5 Text and S10 Fig), with a large pool leading to rapid clearance, in agreement with observations of such clearance facilitated by cross-reactive effector T cells [12,58]. The outcomes were less sensitive to the viral inoculum size (S6 Text and S11 Fig), i.e., *I*_0_, consistent with studies on macaques where different inoculum sizes led to comparable disease outcomes [59].

Our model thus successfully recapitulated the spectrum of outcomes observed following primary SARS-CoV-2 infection. The variations in innate and CD8 T-cell responses in our model allowed this recapitulation. To quantify the influence of the innate and CD8 T-cell responses in determining the outcomes, we next fit our model to patient data from different cohorts, experiencing mild and severe infections.

### Model fits patient data and quantifies differences between mild and severe infections

To our knowledge, datasets with frequent viral load measurements from sputum or saliva samples of severely infected patients do not exist. Measurements from nasopharyngeal (NP) swab samples, however, have been reported [60]. We employed the latter datasets here. To compare between severely and mildly infected patients, we also considered data of NP swab samples from the mildly infected patients above [37,38]. This was necessary despite our fits to the sputum samples above because the dynamics of viral load reported by sputum and NP swab measurements can be distinct [37,38,56,61]. The reasons for this distinction remain poorly understood. The distributions of CD8+ and CD4+ T cells in pulmonary and gastrointestinal mucosa may be distinct [62]. Besides, the local environments, such as the nasal microbiota, might play a role in establishing compartment specific effects [63]. Because both cohorts were studied before the major SARS-CoV-2 variants had emerged [5], we expected the intrinsic growth rate of the virus to be similar in the two cohorts. We, therefore, fixed the parameter *k*_1_ at the value estimated above (Table 2; fixed and random effect values of 4.49/day and 0.28/day, respectively). We normalized the viral load measurements using the maximum viral load across the cohorts.

**Fig 5:**
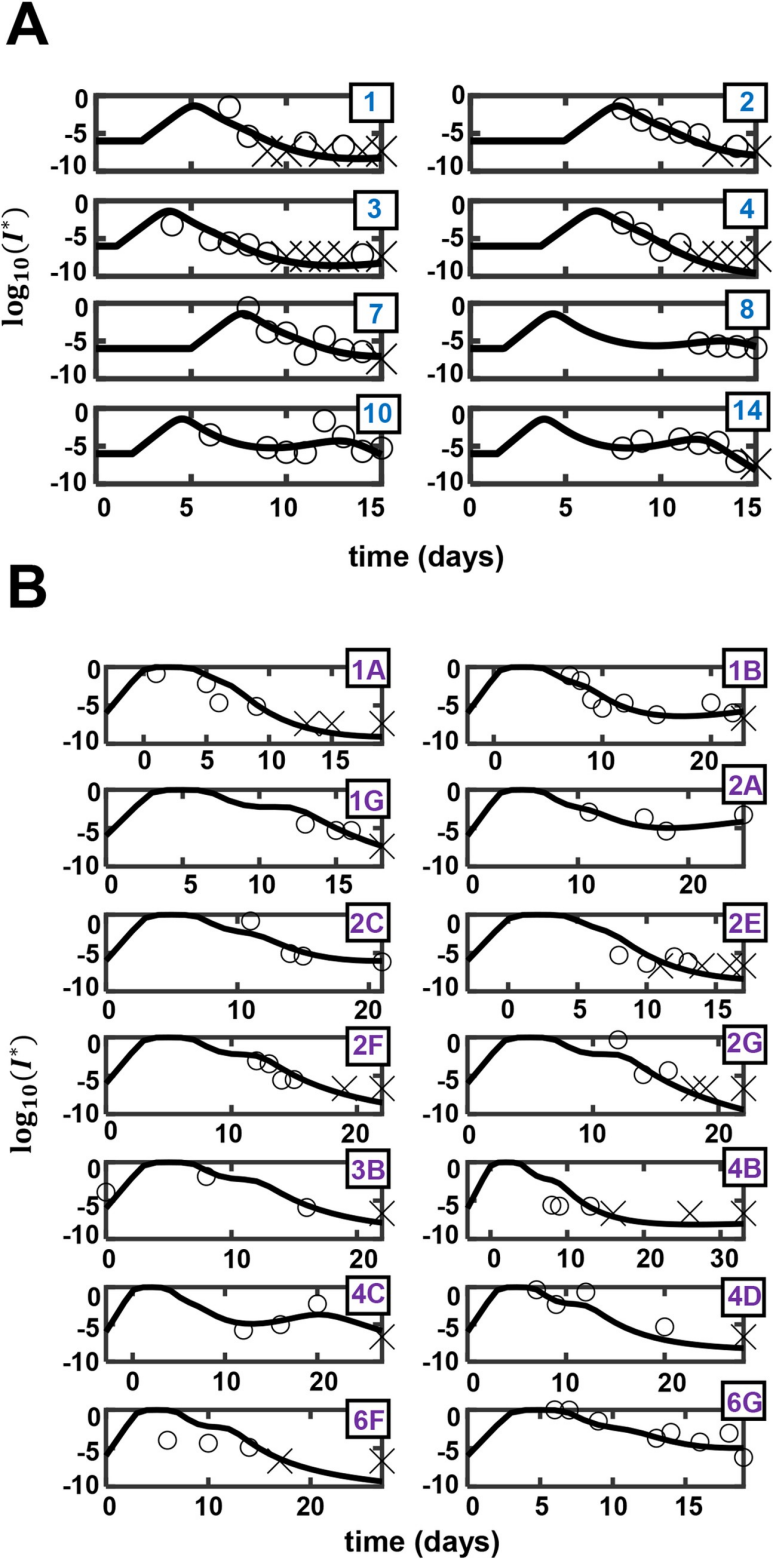
Fits of the model to viral load data from patients with mild and severe symptoms. Best-fits of our model (lines) to data (symbols) of nasopharyngeal viral load from patients with (A) mild and (B) severe symptoms. Cross marks represent data points below the limit of detection. Entries in the boxes show patient IDs as provided in [38] in (A) and the location (row and column) of the subplot in the original figure in [60] from which the data was extracted in (B).

We fit the model first to NP swab data from mildly infected patients up to 15 days post-exposure, as described earlier (Fig 2). The model provided excellent fits to the data (Fig 5A and Tables 4 and S8). Visual predictive check and shrinkage of parameters estimated indicated the reliability of our fits (S12 Fig). Expectedly, the best-fit parameters associated with the sputum and NP swab datasets were different (Tables 2 and 4). For instance, the viral incubation period (*τ*) estimated from the swab dataset was higher than that estimated from the sputum dataset, in agreement with earlier observations that NP swabs might provide a delayed positive RT-PCR result [61]. The trends in the parameter estimates and associated predictions observed in the sputum data, however, were broadly maintained. For instance, the model fits to data from patients who showed a rebound after the first peak indicated delayed and weak CD8 T-cell responses, as was also observed above (S8 Table and S13 Fig). As we did above, we quantified the uncertainties in our individual patient fits and parameter estimates using multiple realizations of the predictions with parameter combinations sampled from distributions conditioned on the individual patient data (S14 Fig and S9 Table).

**Table 4:** Population parameters estimated by fitting the model to NP swab viral load data from mildly infected patients [38] (Fig 5A).

Parameter	Unit	Fixed effect (SD)		Random effect (SD)	
***k***_**3**_	day^-1^	1.62	(0.078)	0.028	(0.035)
$k_{5}^{*}$	day^-1^	54.61	(3.04)	0.34	(0.92)
$E_{0}^{*}\times 10^{3}$	day^-1^	0.65	(0.36)	0.36	(0.26)
$k_{p}^{*}$ ×10^6^	dimensionless	18.11	(7.07)	1.35	(0.93)
***τ***	day	2.44	(0.5)	0.66	(0.19)

Next, we fit our model to data from severely infected patients (Table 5 and Fig 5B). In this dataset, day zero was reported as the time of symptom onset [60]. We, therefore, introduced a parameter *ζ*, representing the time from the start of viral growth to symptom onset (Methods), which we estimated from the fits (instead of *τ*). Our model again yielded excellent fits to the data (Figs 5B and S15 and S10 Table). Visual predictive check and shrinkage of parameters estimated again indicated the reliability of our fits (S16 Fig). We quantified the uncertainties in our individual patient fits and parameter estimates as above (S17 Fig and S11 Table). Following previous studies [30,31,35,55], we also considered a model that allowed exhaustion to depend on the accumulation of antigenic stimulation and found that it had a higher BICc value (298.4) compared to the present model (279) (S7 Text and S18 Fig and S12 Table).

**Table 5:** Population parameters estimated by fitting the model to NP swab viral load data set from severely infected patients [60] (Fig 5B).

Parameter	Unit	Fixed effect (SD)		Random effect (SD)	
***k***_**3**_	day^-1^	1.74	(0.063)	0.039	(0.02)
$k_{5}^{*}$	day^-1^	0.69	(0.053)	0.11	(0.13)
$E_{0}^{*}$ ×10^3^	day^-1^	0.18	(0.07)	0.42	(0.15)
$k_{p}^{*}$ ×10^6^	dimensionless	3712.12	(145.66)	1.22	(0.33)
***ζ***	day	0.0019	(0.014)	18.46	(15.09)

We note that our population estimates of *ζ* showed a small fixed effect and a large random effect (Table 5). This implied that in most patients symptom onset co-occured with the start of viral replication, although large deviations were possible in some individuals. This was consistent with observations from a recent study on human volunteers challenged with a small inoculum of SARS-CoV-2 and monitored closely [64]. In the study, 17 volunteers reported PCR-confirmed infection and a symptom score >2 at any point in 18 days post-inoculation. We estimated *ζ* for these individuals as the difference between the time of the onset of symptoms and the time when the virus was first detected, the latter expected to be close to the start of viral replication. We found that *ζ* had a mode of 0 days and mean of 0.5 days with a standard deviation of 1.8 days. Specifically, 5 participants had *ζ* = 0 days, 2 had *ζ* = 0.5 days and one had *ζ* = 5.5 days. These observations were consistent with our estimates of a small fixed effect and a large random effect of *ζ*.

In the above fits, we used all the data available, including past 15 days of symptom onset, where antibody responses may have arisen. Antibody responses are expected to exert only a minimal influence in primary infection [11]. Nonetheless, we tested the robustness of our fits to possible antibody responses as follows. We refit our model to the above data using data only up to day 15 and, using the resulting best-fit parameter estimates, projected viral loads post day 15. We found that the projected viral loads were in most cases (11 of 14 patients) higher, but only marginally so, than viral loads in our best-fits obtained using all the data (S19 Fig), suggesting a minor role for antibody responses. (In the remaining 3 patients (with IDs 1A, 2A, 6G), the projected viral loads were marginally lower.) Further, the best-fit population parameters were similar to those obtained earlier (S13 Table). This comparison reinforces the notion that antibody responses play only a minor role in primary infection, further justifying the assumptions in our model.

**Fig 6:**
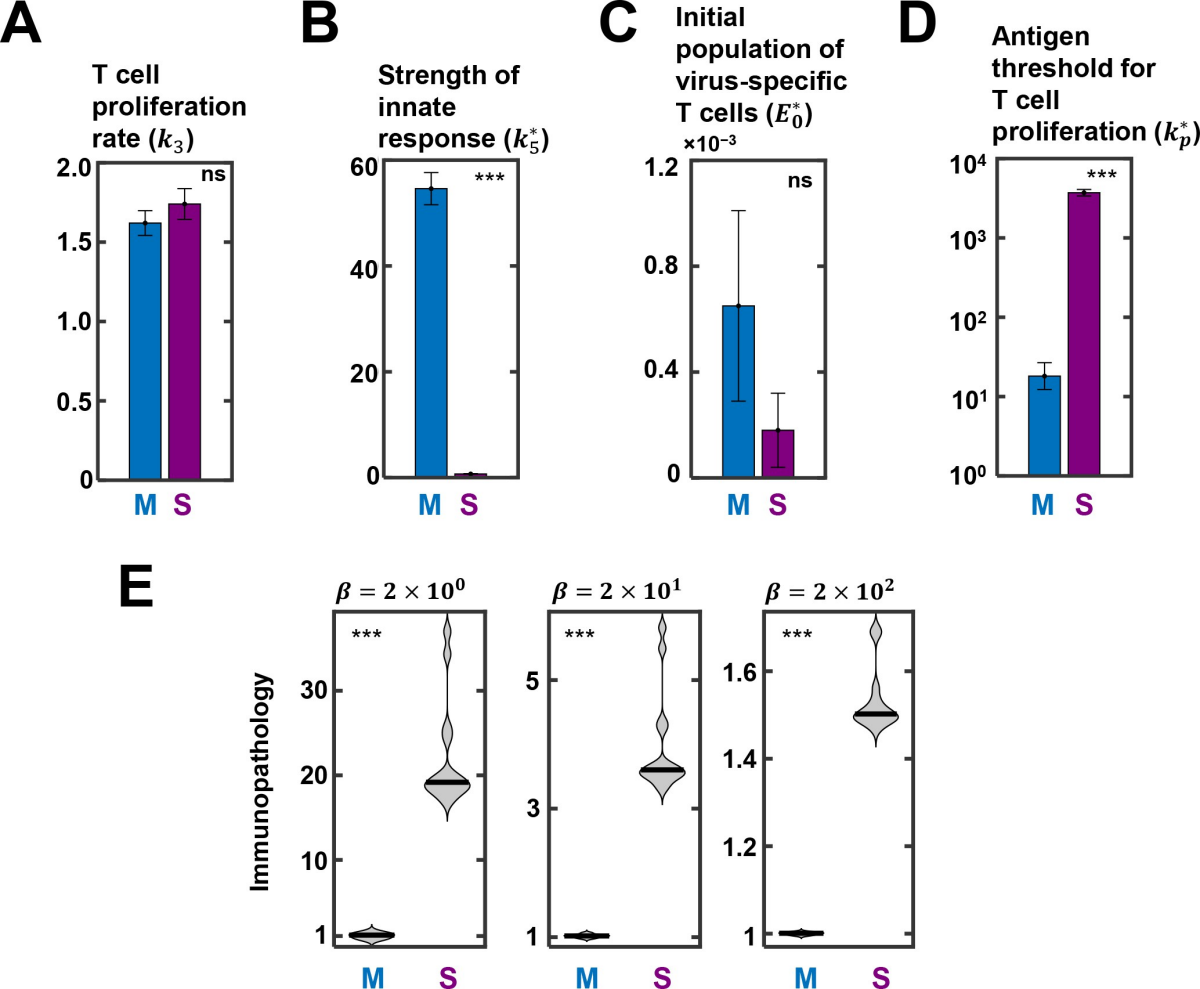
Comparison of parameter estimates between mildly and severely infected patients. Best-fit estimate of (A) T cell proliferation rate (*k*_3_), (B) strength of innate response ($k_{5}^{*}$), (C) initial population of virus-specific T cells ($E_{0}^{*}$), (D) antigen threshold for T cell proliferation ($k_{p}^{*}$) between mildly (M) and severely (S) infected patients (see Fig 5). ns: not significant, ***: p < 0.0001. (E) The distributions of model-predicted immunopathology of the two patient cohorts using different values of *β*, reflecting the relative contribution of cytokines to immunopathology. The solid lines in the violin plots are medians.

We now compared the parameter estimates between mildly and severely infected patients to identify the key differences between the patient groups. Among the fit parameters, *k*_3_, the rate of CD8 T-cell expansion, was similar between the mild and severely infected patients (Tables 4 and 5 and Fig 6A). Interestingly, *k*_5_, the strength of the innate response, was starkly different between the two cohorts, with a value (54.6 d^-1^) nearly 80-fold higher in the mildly infected cohort than the severely infected cohort (0.69 d^-1^) (Tables 4 and 5 and Fig 6B). The initial level and/or activity of specific CD8 T-cells, i.e., *E*_0_, was higher in the mildly infected patients (Tables 4 and 5) but the difference did not achieve statistical significance (Fig 6C). Finally, *k*_*p*_, the antigen threshold for triggering CD8 T-cell proliferation, was remarkably different between the cohorts (Tables 4 and 5 and Fig 6D).

The threshold was >200-fold higher (3712 vs. 18) in the severely infected patients compared to the mildly infected ones. The mounting of the CD8 T-cell response was thus delayed in severely infected patients (see also Fig 4C); a >200-fold larger pool of infected cells had to accumulate before a significant CD8 T-cell response could be mounted. The origins of the differences remain poorly elucidated. It is possible that HLA polymorphisms, which could directly affect CD8 T-cell activation, may underlie the differences. Indeed, specific HLA alleles have been argued to be significantly more associated with severity and mortality in COVID-19 [65–67].

For confirmation, using the best-fit parameter values, we estimated the immunopathology in the cohorts (Fig 6E). As expected, a markedly higher immunopathology was predicted in the severely infected patients than the mildly infected patients. This was true of all the metrics we used to estimate immunopathology (see S1 Text). Further, we considered variations in the relative contribution of cytokines (or innate immune responses) versus CD8 T-cells to immunopathology in calculation of tissue damage, *D*, by varying *β* (Eq 4). The higher the value of *β*, the greater the relative contribution from cytokines. In all cases, the immunopathology in the severely infected individuals was significantly higher than in the mildly infected individuals.

We also estimated the within-host basic reproductive ratio *R*_0_ using our model to assess whether the difference in the severity of infection arose from the early stages of growth of the infection. *R*_0_ is defined as the number of infected cells produced by one infected cell in a wholly susceptible target cell population. We realized that in the early stages of infection, when the effector response is yet to be mounted, virus induced cytopathy can be a significant contributor to infected cell death. We recall that effector cell killing of infected cells occurs at the rate of $E_{0}^{*}∼10^{-4}-10^{-3}$ day^-1^, whereas estimates of virus induced cytopathy from in vitro studies [68,69] are *δ*~0.3−0.35 day^-1^. We obtained the latter estimates from two studies: In one study, where fully differentiated primary human alveolar epithelial cell cultures were infected by 0.1 MOI SARS-CoV-2, about 30 of 50 infected cells imaged were found to be apoptotic 72 h after infection [68]. In the second study, cell lines with a vector containing SARS-CoV-2 ORF3a, the viral protein thought to trigger apoptosis in SARS-CoV-2 infected cells, 30% of the transfected cells were apoptotic 24 h after transfection [69]. A first order death process would yield *δ*~0.3−0.35 day^-1^ from these observations. Accounting for the latter process in our model (by adding the term −δI* to right hand side of Eq 5) and using the next generation matrix method [70], we derived $R_{0}=k_{1}/(E_{0}^{*}+\delta )$. Using the above parameter values, we estimated $R_{0}\approx \frac{k_{1}}{\delta }\approx 13-15$, consistent with current estimates of *R*_0_≈10 [71], and similar for both mildly and severely infected patients we examined. Thus, the differences in severity appeared to arise from the differences in the immune responses ‘after’ the initial stages of infection. (We note that once the immune response is mounted, effector killing (E*~5 day^-1^; see Figs 2 and S13 and S15) dominates viral cytopathicity (*δ*~0.3 day^-1^), justifying ignoring the −δI* in our model.)

In summary, mildly infected patients appeared to mount a nearly 80-fold swifter innate immune responses and a CD8 T-cell response that was over 200-fold more sensitive to antigen. These estimates quantified the underlying differences in the strength and the timing of the innate and CD8 T-cell responses between individuals who readily cleared the infection and those who suffered severe disease in the two cohorts we studied.

## Discussion

The extreme heterogeneity in the outcomes of SARS-CoV-2 infection across infected individuals has been puzzling. Here, using mathematical modeling and analysis of patient data, we argue that the heterogeneity could arise from the variations in the strength and the timing of the innate and the CD8 T-cell responses across individuals. In our model, when the CD8 T-cell arm was strong, clearance of the infection resulted. When the innate arm was also strong, asymptomatic or mild infections resulted. If the innate arm was weak, the peak viral load was large, resulting in higher immunopathology and moderate symptoms. When the CD8 T-cell response was strong but delayed, a predator-prey type interaction between the innate arm and the virus resulted, causing multiple peaks in the viral load. These oscillations ended when the CD8 T-cell response was mounted, and clearance ensued. When the CD8 T-cell response was weak but the innate arm was strong, prolonged infection could result before clearance. When both the arms were weak, severe infection including mortality followed. These predictions offer a plausible qualitative explanation of the heterogeneous outcomes of SARS-CoV-2 infection. The predictions also offer a synthesis of the numerous independent and seemingly disconnected clinical observations associated with the outcomes. Furthermore, our model provided excellent fits to longitudinal viral load data from patients and quantified the differences in the strength and the timing of the innate and CD8 T-cell responses between mildly and severely infected patients. The best-fits indicated that the innate immune response was nearly 80-fold swifter and the CD8 T-cell response over 200-fold more sensitive to antigen in mildly infected individuals than those who suffered severe disease. These estimates offer quantitative insights into the underlying within-host viral dynamics in patients suffering mild and severe disease and may inform intervention strategies aimed at preventing severe disease.

Several mathematical models of within-host SAR-CoV-2 dynamics have been developed and have offered valuable insights [72,73]. For instance, they have helped estimate the within-host basic reproductive ratio [60,74,75] and assess the effects of drugs and vaccines [26,43,76–80]. Attempts have been made to capture the role of the immune system in disease progression and outcome [43,77,79,81–88]. Some models have also analysed the same datasets that we have used here [38,43,60,75,77]. Available models, however, have either not been shown to fit longitudinal patient data or to describe the entire range of outcomes realized. To our knowledge, ours is the first study to describe the outcomes realized comprehensively using a mathematical model that is consistent with patient data.

Our model predictions may help better understand known demographic correlates of disease severity and mortality, such as gender, age and comorbidities. Male patients trigger higher levels of peripheral cytokine expression and elicit weaker CD8 T-cell responses than female patients [89], resulting in more frequent severity and mortality in males [45]. The increased mortality in the elderly is caused by immunosenescence, which is associated with decreased proliferative capacity of lymphocytes and impaired functionality of innate immune cells [90]. Increased mortality is also associated with comorbidities, such as type-2 diabetes [91], where uncontrolled production of proinflammatory cytokines and inappropriate recruitment of lymphocytes is observed [92]. These observations are consistent with our predictions, where more severe infections would result from weaker CD8 T-cell responses and/or unregulated innate immune responses. Our model could be tested by analyzing longitudinal datasets categorized by the above correlates to draw quantitative inferences of their influence on disease severity.

Factors other than the demographic correlates above could also contribute to variations in the innate and the CD8 T-cell responses across individuals. For instance, certain mutations, reported in a subset of severe COVID-19 patients, may preclude a potent interferon response [53]. A section of severely infected patients is reported to harbor neutralizing autoantibodies against interferons [28,93]. Overzealous production of antibodies against SARS-CoV-2 might inhibit the pathway for interferon-mediated induction of antiviral genes [94]. Further, in vitro studies suggest that different SARS-CoV-2 proteins can inhibit the TBK1-IRF3 pathway or the JAK/STAT pathway at several signaling nodes, adversely affecting interferon production and/or signaling [95]. Variability in the CD8 T-cell response may come from different precursor populations, due for instance to variable prior exposure to circulating human coronaviruses [96]. Patients pre-exposed to other coronaviruses or rhinoviruses harbor populations of effector T-cells that might cross-react with SARS-CoV-2 antigens and contribute to the early clearance of the infection [96,97]. Population-level variations in effector cell frequencies [98] and inter-individual heterogeneity in lymphocytic gene expression patterns [99] may also contribute to the variability in the CD8 T-cell response. Our model could also be tested by quantifying the effects of the latter factors on disease severity and comparing the results to quantitative experimental data.

CD8 T-cell exhaustion has been proposed as an evolutionary design to prevent mortality due to immunopathology [30,100]. By preventing extensive tissue damage due to CD8 T-cell killing of infected cells, exhaustion can avert mortality. The price of reduced CD8 T-cell efficiency is often persistent infection, as seen with HIV and hepatitis C [30]. With severe SARS-CoV-2 infection, although extensive CD8 T-cell exhaustion is seen, it appears inadequate to prevent mortality; immunopathology caused by proinflammatory cytokines dominates. Potent activation of the NF-*κ*B pathway by components of the SARS-CoV-2 virion may trigger the production of detrimental proinflammatory cytokines [101,102]. Heightened interferon expression in the lung [9,17,18,103,104] impairs cell proliferation, impeding tissue repair after proinflammatory cytokine-mediated immunopathology [105]. Moreover, interferons may synergize with proinflammatory cytokines to fuel immunopathology by triggering cell death pathways [106,107]. In contrast, immunopathology due to CD8 T-cells appears minimal. CD8 T-cells infiltrate the alveolar tissues of COVID-19 patients [104] and can kill infected cells. At the peak of the infection, 10^4^−10^6^ cells are estimated to be infected out of the 10^11^ estimated target cells in the respiratory tract [39]. Thus, direct CD8 T-cell killing of infected cells would affect a small fraction of cells in the respiratory tract. This may also explain why long-term persistence appears rare with SARS-CoV-2 infection: Inducing CD8 T-cell exhaustion, the common strategy underlying persistent infection, can only minimally affect immunopathology dominated by cytokines. We speculate that the absence of persistence may be a general feature of those viral infections where immunopathology is predominantly cytokine mediated. Indeed, hypercytokinemia has been associated with the fatal outcomes following influenza A (H5N1) infection [108]. Nonetheless, regardless of the relative contributions of CD8 T-cells and innate immune responses to immunopathology, which remain to be quantified, severely infected patients consistently displayed elevated levels of immunopathology than mildly infected patients in our analysis.

A strategy of great interest today for reinvigorating exhausted CD8 T-cells is the use of immune checkpoint inhibitors [109]. The inhibitors are approved for use in certain cancers. Because of their promise, five clinical trials are underway for testing their efficacy in treating severe COVID-19, of which one (NCT04333914) is on cancer patients, and the remaining (NCT04413838, NCT04343144, NCT04356508, and NCT04268537) are on non-cancer patients infected by SARS-CoV-2 [110]. A major risk of checkpoint inhibitor therapy is increased immunopathology due to a heightened CD8 T-cell response. Based on our model predictions and arguments above, we speculate that with COVID-19, the risk of increased immunopathology from immune checkpoint inhibitor therapy is likely to be small, given the predominance of cytokine-mediated pathology. A retrospective analysis of melanoma patients showed that checkpoint inhibitor therapy did not increase the risk of mortality due to COVID-19 [111]. Rather, the beneficial effects of an improved CD8 T-cell response may outweigh any minimal enhancement in immunopathology. Indeed, a recent study reported that immune checkpoint inhibitors can increase the T cell response in SARS-CoV-2 infected melanoma patients, without interfering with early interferon induction or aggravating inflammation [112].

Our model could be tested further by examining whether it can recapitulate the implications of different therapeutic interventions [113] and of emerging viral variants [114] on disease outcomes. Given the mechanisms of action of available drugs and drug candidates [113], their effects on typical individuals in the mild or severe infection categories could be simulated using the corresponding modal parameter estimates we identified for the respective categories in this study. These could then be tested against measurements from patients administered the interventions. Several recently identified circulating mutants are known to be more infectious/transmissible than the original SARS-CoV-2 strain and to escape immune responses [115]. These characteristics could be incorporated in our model by suitably increasing the infectivity (e.g., see [116]) and/or decreasing the strength of the immune response, to simulate how emerging strains could alter the overall severity of the infection, which in turn could be tested against data from patients infected by those strains. We recognize that to estimate the effects of such variations at the population level, knowledge of how the parameter values in our model, particularly those defining the innate and CD8 T-cell responses, are distributed across individuals in a population would be required. With hepatitis C virus infection, for instance, the distribution of the strength of interferon responsiveness across individuals quantitatively predicted the fraction of individuals that spontaneously cleared the infection [117,118] and together with the distribution of the CD8 T-cell response captured the success of interferon-based and other therapies [47,117–119]. With HIV, distributions of underlying parameters predicted the distribution of viral breakthrough times following antibody therapy [120]. Such predictions with SARS-CoV-2, once parameter distributions become available, may help refine clinical and epidemiological projections of healthcare requirements.

Our study has limitations. First, we neglected the role that cytokines play in the expansion of CD8 T-cells [121] because fits of our model incorporating such an effect to the available data were poor (S3 Text). Perhaps, a larger patient cohort may improve the fits and allow incorporating the latter effect. Second, our model did not incorporate any negative effect of immunopathology on the immune response; for instance, lymphopenia [15,122], which is generally thought to be caused by immunopathology, could compromise the immune response. Third, we employed a simplified model of CD8 T-cell exhaustion, following earlier studies [30,31,33], which allows exhaustion to be reversed fully upon lowering antigen levels. Recent studies have demonstrated that exhaustion is reversible only in a subset of exhausted cells [109]. CD8 T-cells can also exhibit more complex dynamics including collective effects [123]. Future studies may overcome the above limitations and yield more accurate predictions and insights. Notwithstanding, given the ability of our model to fit multiple longitudinal patient datasets as well as offer explanations of several confounding clinical observations, we expect our key inferences regarding the roles of the innate and CD8 T-cell responses in determining the heterogeneous outcomes of SARS-CoV-2 infection to hold.

## Methods

### Study data

Viral load data utilized for this study were digitized from previously published clinical studies [38,60]. Data from infected individuals with at least three measurements above detection limits within 20 days of symptom onset were included in our analysis. Thus, we had 8 patients with mild symptoms [38] and 14 patients with severe symptoms [60]. In the former cohort, all individuals were young and had no comorbidities. In the latter, 80% were hospitalized with symptoms of severe disease. They had different comorbidities, such as diabetes, hypertension and obesity, and 7 were above 65 years of age. The clinical measurements were digitized using a custom script in the MATLAB (version R2020a) image analysis toolbox (www.mathworks.com).

### Parameter estimation and model selection

The extracted datasets were used for fitting different models. Fitting was done following the nonlinear mixed effects modeling approach. In this approach, model parameters are assumed to be drawn for each individual from underlying population distributions. The objective of the fitting exercise is to estimate the means and the variances of the distributions, termed ‘population parameters’, by fitting data of all the individuals simultaneously. Values sampled from these distributions, termed ‘individual parameters’, then recapitulate individual patient data. Briefly, the measurement, *y*_*ij*_, made on individual *i* at time point *t*_*ij*_ is expressed as

$$y_{ij}=f(t_{ij},\varrho_{i})+e_{ij}$$

where the nonlinear dynamical model *f* evaluated at time *t*_*ij*_ and using the parameters *ϱ*_*i*_ representing individual *i* yields a prediction of the observation (or measurement) with the residual error *e*_*ij*_. The typical parameter *ϱ* in the model is assumed to follow a lognormal distribution across the individuals in the population so that its value *ϱ*_*i*_ for individual *i* can be written as

$$\log\left(\varrho_{i}\right)=\log\mu +\Psi_{i}$$

where *μ* is the population mean of *ϱ*, also known as the ‘fixed effect’ and Ψ_*i*_~*N*(0,*σ*) represents the ‘random effect’, assumed to follow a normal distribution with mean zero and standard deviation *σ*. The error *e*_*ij*_ is assumed to be a combination of constant (*a*_*i*_) and proportional (*b*_*i*_) contributions, so that

$$e_{ij}=(a_{i}+b_{i}f(t_{ij},\varrho_{i}))\epsilon_{ij}$$

where $\epsilon_{ij}∼N(0,1)$ is a standard normal random variable.

We performed fitting using the stochastic approximation expectation maximization (SAEM) algorithm in Monolix 2020R1 (www.lixoft.com). The fitting yielded best-fit population parameters, as their fixed and random effects, the latter characterized using *σ*, and individual parameters together with a characterization of the errors. To compare alternative models, we estimated the corrected Akaike information criterion (AICc) and the corrected Bayesian information criterion (BICc) for each model. The model with the lowest AICc/BICc was selected for further mathematical analysis (S3 Text).

To ensure that the fitting captured the basic trends of the viral dynamics, we right censored the peaks in the data for each patient. This ensured that parameter combinations that underpredicted the peaks were disfavored. For patients 1 and 2, where a relatively longer viral incubation was evident from visual inspection of the data, we introduced left censored data points of the infection load in the first few days so that the number of infected cells did not rise in these early time points. (Note that left censoring a data point in Monolix implies that the data point is below the lower limit of detection, and the fitting algorithm disfavors parameter combinations that overpredict the value at that data point. Similarly, the algorithm disfavors parameter combinations that underpredict a right censored data point.) We fit the following model equations to the data:

$$\frac{dI^{*}}{dt}=[k_{1}(1-X^{*})I^{*}(1-I^{*})-I^{*}E^{*}]H(t-\tau )$$ (5)

$$\frac{dE^{*}}{dt}=\left[k_{3}(\frac{I^{*}E^{*}}{k_{p}^{*}+I^{*}})-k_{4}(\frac{I^{*}E^{*}}{k_{e}^{*}+I^{*}})\right]H(t-\tau )$$ (6)

$$\frac{dX^{*}}{dt}=[k_{5}^{*}I^{*}-k_{6}X^{*}]H(t-\tau )$$ (7)

These equations without the Heaviside functions (*H*(*t*−*τ*)) were derived by rescaling our mathematical model (Results) using the following relations: $k_{p}^{*}=k_{p}/I_{max},\;k_{5}^{*}=k_{5}.I_{max}.\varepsilon_{I},\;I^{*}=I/I_{max},\;X^{*}=X.\varepsilon_{I},\;k_{e}^{*}=k_{e}/I_{max}$, and E* = *E*. *k*_2_. Next, we introduced the Heaviside functions, *H*(*t*−*τ*), which equals 1 when *t*>*τ* and 0 otherwise, to account for the delay in viral replication post exposure, *τ*. Visual inspection of the dataset indicated that at least for some patients, the viral load did not start rising immediately after exposure. The dynamical events of the infection were thus initiated after the duration *τ*, which we estimated from the fits. Further, as elaborated in the results section, to fit the datasets from mild patients, we fixed *k*_4_ = 0. We assumed the following initial conditions: $I_{0}^{*}=10^{-6},\;X_{0}^{*}=0$. The former initial condition was based on the estimate that the maximum number of infected cells at the peak of the infection might be ~10^6^ cells [39]. Further, we tested the sensitivity of the fits to this assumption (S4 Table). The value of $E_{0}^{*}$ was estimated from the fits. We fixed *k*_6_ to 0.2 day^-1^ following previous studies [124,125]. We carried out a formal structural identifiability analysis of the rescaled model using SIAN in the Maple platform (www.maplesoft.com) [126]. All the fit parameters of the model, $k_{1},\;k_{3},\;k_{5}^{*}$, and $k_{p}^{*}$, and the initial conditions: *I*(0), *E*(0), and *X*(0) were structurally globally identifiable, when a continuous and noise-free input for *I* was supplied.

We used lognormal distributions for all parameters except *k*_1_ and *k*_*p*_. Logit distributions were used for the latter parameters along with biologically relevant ranges for their values. *k*_1_ and *k*_*p*_×10^6^ were thus allowed to vary in the ranges 2–7 and 10–5000, respectively [30,74]. The fitted population parameters (Tables 2 and 4 and 5) and individual parameters (Tables 2 and S8 and S10) were obtained from Monolix, and further simulations were run in MATLAB. To obtain uncertainties in the individual fits, we generated 50 realizations by sampling parameter combinations from the conditional parameter distributions for each patient and estimated the associated means and standard deviations (S4 and S14 and S17 Figs and S5 and S9 and S11 Tables). We also performed visual predictive checks and assessed the shrinkage of the parameters within the Monolix environment to assess the reliability of our fits and parameter estimates (S3 and S12 and S16 Figs).

For fitting the viral load dataset from severe patients, for which day 0 was the symptom onset time, the model calculations started from the time point −*ζ*. We recognized that viral propagation may start before symptom onset, at a time determined by *ζ*. We thus wrote:

$$\frac{dI^{*}}{dt}=[k_{1}(1-X^{*})I^{*}(1-I^{*})-I^{*}E^{*}]H(t+\zeta )$$ (8)

$$\frac{dE^{*}}{dt}=\left[k_{3}(\frac{I^{*}E^{*}}{k_{p}^{*}+I^{*}})-k_{4}(\frac{I^{*}E^{*}}{k_{e}^{*}+I^{*}})\right]H(t+\zeta )$$ (9)

$$\frac{dX^{*}}{dt}=[k_{5}^{*}I^{*}-k_{6}X^{*}]H(t+\zeta )$$ (10)

We fixed the following parameters for these fits: *k*_4_ = 2 day^-1^; *k*_*e*_ = 7×10^5^ cells; *k*_6_ = 0.2 day^-1^.

### Selection of parameters not estimated in the fitting

In our fitting exercise for the mildly infected patients, we ignored the parameters associated with exhaustion and immunopathology. We obtained the latter parameters for subsequent fits and calculations as follows. We chose *k*_4_ from a previously published analysis [30]. We then chose *k*_*e*_ such that no major effect of exhaustion was observed for the simulations corresponding to the best-fits to the mildly infected patient data (S20 Fig). This ensured internal consistency with our assumption and agreement with observations of minimal pathology in mildly infected patients. The parameters for immunopathology were either taken from a previously published source [30] or assumed. Variations in these parameters did not alter our inferences (S21 Fig). The following values of the parameters were used in all simulations, unless stated otherwise: *k*_4_ = 1.5 day^-1^; *k*_*e*_ = 7×10^5^ cells; *α* = 1×10^4^ cells^-1^day^-1^; *β* = 2×10^4^ day^-1^; and *γ* = 0.5 day^-1^. The parameters *α*, *β* and *γ* which describe the dynamics of tissue damage (Eq 4), are unknown constants; our results were not sensitive to their values in predicting the relative extents of immunopathology across different disease severity categories (S8 Text and S21 Fig).

### Fixed points and linear stability analysis

We solved the model equations for steady state and obtained the following fixed points:

1.
I=0,E≥0,X=0

2.
$I=\frac{k_{6}}{k_{5}},\;E=0,\;X=1$

3.
$I=1,\;E=0,\;X=\frac{k_{5}}{k_{6}}$

4.
$I=\frac{k_{e}k_{3}-k_{p}k_{4}}{k_{4}-k_{3}},\;E=k_{1}(1-\frac{k_{5}}{k_{6}}I)(1-I),\;X=\frac{k_{5}}{k_{6}}I$

MATLAB (version R2020a) was used to obtain the fixed points and to determine their stability. Individual fixed points and their corresponding Jacobian matrices were estimated using the Symbolic Math Toolbox (www.mathworks.com). Calculation of the eigenvalues and eigenvectors for individual fixed points yielded the nature of their stability and facilitated determination of the phase portraits (S5 Text). For the steady-state analysis, estimated population parameter values were used (Table 2).

## Supporting information

S1 Fig The schema of calculation of immunopathology. The peak of the instantaneous tissue damage (*D*) was detected for the simulation with the population parameters (left). A line parallel to the X-axis was drawn at the half-maximal level of *D*. The two intercepts of the curve of *D* with the horizontal line were identified. The area under the curve (AUC) was calculated within these half-maximal intercepts. The same threshold was used for parameters associated with an individual (right) and the AUC was calculated. The ratio of the latter AUC and the former was used as an estimate of the extent of immunopathology, *P*. Hence, immunopathology for model simulations with any parameter set is: *Immunopathology*_*test*_*parameters*_ = *AUC*_*test*_*parameters*_/*AUC*_*population*_*parameters*_.
(TIF)

S2 Fig Comparison of the immunopathology estimated by different metrics. (A) The dynamical profiles of tissue damage (*D*) are shown for the simulations in Fig 4A. The black annotated triangles at the top and right indicate the nature and the direction of the variation of the indicated parameters. Vertical dotted lines are peaks in *D*. The blue horizontal dashed line represents the common threshold, as indicated in metric V (S1 Text), and the purple shaded region is its AUC. The red dashed line represents the threshold calculated following metric III, and the light orange shaded area its AUC. (B)-(G) Colour maps in 5×5 grids represent the immunopathology scores calculated for the subplots shown in (A) following different metrics (see S1 Text). The empty grid represents diverging immunopathology (see Fig 4).
(TIF)

S3 Fig Shrinkage and visual predictive check of the fits to sputum viral load data from mild patient cohort. (A) Parameter shrinkage. For each fit parameter (individual panels), the distribution of the population parameter (black line) and values sampled from the conditional distributions of the estimates of the individual parameters (histogram) are shown along with estimates of the shrinkage. Shrinkage = 1−(*var*(*η*)/*ω*^2^), where *ω* is the standard deviation of the random effect, and *var*(*η*) is the variance of the samples drawn from the conditional distributions of individual parameter estimates. (B) Visual predictive check. The blue segmented lines represent the trends of the observed data, and the blue and pink patches represent the trends of the model outputs generated via simulations. The lower, middle and upper blue lines represent the 10th, 50th and 90th percentile of the data, respectively. The patches indicated 90% confidence intervals for the median (middle), the 10th percentile (top) and the 90th percentile (bottom) of the simulations. Overall, the parameter shrinkages are low and the simulations correctly capture the variability in the data, indicating that the fits are good.
(TIF)

S4 Fig Quantifying uncertainties in individual fits in Fig 2. The thin grey curves in each plot show model predictions using parameter combinations sampled from conditional distributions based on individual patient data fits. 50 realizations are presented for each patient. The error bars indicate standard deviations from these realizations. The bold curve in each plot is the prediction using the mode of the conditional distribution, as shown in Fig 2 in the main text. The open circles represent the data points. The patient IDs are the same as in Fig 2, and shown in boxes with blue numbers. The means and standard errors of the parameter values are listed in S5 Table.
(TIF)

S5 FigModel fits to data in the absence of effector response.Fits (solid lines in panel A) of our model (Eqs 5–7) with $E_{0}^{*}=0$ to data (symbols) from patients 7, 8, and 10. Fits from Fig 2 are reproduced for comparison (dashed lines). Corresponding predictions of the innate immune response (panel C), and the associated phase plane plots indicating prolonged oscillations. Best-fit parameter estimates for the three patients were as follows. Patient 7: *k*_1_ = 4.49 days^-1^, $k_{5}^{*}=2.98$ days^-1^ and *τ* = 1.58 days; Patient 8: *k*_1_ = 4.62 days^-1^, $k_{5}^{*}=3.17$ days^-1^ and *τ* = 0.58 days; and Patient 10: *k*_1_ = 4.54 days^-1^, $k_{5}^{*}=3.11$ days^-1^ and *τ* = 0.88 days.(TIF)

S6 FigSensitivity of the dynamics of innate response to *k*_5_.The width of the curves is proportional to the strength of ***k***_**5**_. (A) and (B) represent the dynamics of *X* and *I*, respectively. The population estimates (fixed effects) of the parameters estimated in Fig 2 (Table 2) were used. Parameter values used: *k*_1_ = 4.49/day, *k*_3_ = 0.74/day, $k_{5}^{*}=2.83$/day, $E_{0}^{*}=6.65\times 10^{-3}$/day, $k_{p}^{*}=2.497\times 10^{-4},\;\tau =1.51$ day, *k*_6_ = 0.2/day, *k*_4_ = 1.5/day, $k_{e}^{*}=0.7$, *α* = 1.0×10^4^, *β* = 2.0×10^4^/day, *γ* = 0.5/day. The fold-changes for variation in $k_{5}^{*}$ are: 0.5, 0.75, 1, 2, 5.(TIF)

S7 FigCD8 T-cell exhaustion plays an important role in prolonged SARS-CoV-2 infection.(A) Effect of simultaneous variation of parameters determining the strengths of innate response and CD8 T-cell exhaustion on the trajectory of the infection is shown. The black annotated triangles at the right and the top depict the nature and the direction of the variation of the indicated parameters. For instance, *k*_4_ increases top to bottom and *k*_5_ is increases from right to left. Individual subplots show the dynamics of infected cells, effector CD8 T-cell response and innate immune response. Each subplot is a double Y-axis plot. The left Y-axis shows the normalized infected cell dynamics. The right Y-axis shows the other two species. The colored patches at the top of the subplots represent the extent of immunopathology. The range of immunopathology is given by the color scale at the bottom. On the left-side of the color scale, a separate legend denotes the texture used for depicting unbounded immunopathology (see text). Unity on the colorscale indicates the immunopathology quantified in the central subplot (subplot with an arrowhead), calculated using the population parameters estimated from Fig 2. (Table 2, see text). The population estimates (fixed effects) of the parameters estimated in Fig 2 (Table 2) were used. Parameter values used: *k*_1_ = 4.49/day, *k*_3_ = 0.74/day, $k_{5}^{*}=2.83$/day, $E_{0}^{*}=6.65\times 10^{-3}$/day, $k_{p}^{*}=2.497\times 10^{-4},\;\tau =1.51$ day, *k*_6_ = 0.2/day, *k*_4_ = 1.5/day, $k_{e}^{*}=0.7,\;\alpha =1.0\times 10^{4},\;\beta =2.0\times 10^{4}$/day, *γ* = 0.5/day. Variations in *k*_4_ are obtained as the following fold-changes to the above value: 0.35, 0.75, 1, 2, 3. The fold-changes for variation in $k_{5}^{*}$ are: 0.5, 0.75, 1, 2, 5. Values of $k_{e}^{*}$ used in (B) are: 0.01, 0.1, 0.3, 0.5, 0.7.(TIF)

S8 Fig Recapitulating dynamics in prolonged SARS-CoV-2 positive patients. (A) A model simulation with long-duration infection is depicted. Tuning the parameters *k*_5_, *k*_*e*_, and *k*_4_ allowed realization of the long-duration infection scenarios. (B) Tissue damage profiles with the nominal value of *β* (left) and a ten-fold lower value (right). Note that in the right panel, the initial peak of tissue damage is 10% of that in the left panel.
(TIF)

S9 Fig Flowchart depicting parameter regimes defining the stability of fixed point 2. See S5 Text for a description of the parameter regimes and stability criteria.
(TIF)

S10 Fig Monostability and bistability, sample trajectories and associated immunopathology. (A) Trajectories in 3D space defined by infected cells, CD8 T-cells and cytokine-mediated innate response for parameter combinations where clearance alone is a stable fixed point. Each trajectory uses different initial conditions. The colors of the trajectories represent the immunopathology associated, defined in the scale bar at the top. Immunopathology corresponding to population parameter estimates (Table 2) is represented by unity on the color scale. (B) Dashed red line is a trajectory headed towards fixed point 2. For such trajectories, immunopathology was typically unbounded in our model.
(TIF)

S11 Fig Dependence of infection dynamics on viral inoculum size. (A) Infected cells dynamics with varying initial infected cell pool sizes (1, 10, 50, 100 cells). (B) Immunopathology of the 4 trajectories compared with the calculated immunopathology for parameters representing severely infected patients (Table 5).
(TIF)

S12 Fig Shrinkage and visual predictive check of the fits to nasopharyngeal viral load data from mild patient cohort. (A) Parameter shrinkage. For each fit parameter (individual panels), the distribution of the population parameter (black line) and values sampled from the conditional distributions of the estimates of the individual parameters (histogram) are shown along with estimates of the shrinkage. Shrinkage = 1−(*var*(*η*)/*ω*^2^), where *ω* is the standard deviation of the random effect, and *var*(*η*) is the variance of the samples drawn from the conditional distributions of individual parameter estimates. (B) Visual predictive check. The blue segmented lines represent the trends of the observed data, and the blue and pink patches represent the trends of the model outputs generated via simulations. The lower, middle and upper blue lines represent the 10^th^, 50^th^ and 90^th^ percentile of the data, respectively. The patches indicated 90% confidence intervals for the median (middle), the 10^th^ percentile (top) and the 90^th^ percentile (bottom) of the simulations. Overall, the parameter shrinkages are low and the simulations correctly capture the variability in the data, indicating that the fits are good.
(TIF)

S13 Fig The innate and adaptive response predicted from model fits to the Wӧlfel et al. swab data. (A) The numbers in the boxes on the top of each plot represents the patient IDs as provided in Bӧhmer et al. [37]. The blue area plots represent the predicted innate immune response corresponding to the infection dynamics shown in Fig 5A in the main text. (B) The green area plots show the predicted dynamics of CD8 T-cell mediated adaptive immune response.
(TIF)

S14 Fig Quantifying uncertainties in individual fits in Fig 5A. The thin grey curves in each plot show model predictions using parameter combinations sampled from conditional distributions based on individual patient data fits. 50 realizations are presented for each patient. The error bars indicate standard deviations from these realizations. The bold curve in each plot is the prediction using the mode of the conditional distribution, as shown in Fig 5A in the main text. The open circles represent the data points, and crosses represent data points below detection limit. The patient IDs are the same as in Fig 5A, and shown in boxes with numbers in blue. The means and standard errors of the parameter values are listed in S9 Table.
(TIF)

S15 Fig The innate and adaptive response predicted from model fits to the Néant et al. swab dataset. (A) The blue area plots represent the predicted innate immune response corresponding to the infection dynamics shown in Fig 5B in the main text. The alpha numeric entries in the boxes indicate the position of the plots in the original figure in Neant et al. [60] (see Fig 5B). X-axes represent time in days, post symptom onset. (B) The green area plots show the predicted dynamics of CD8 T-cell mediated adaptive immune response.
(TIF)

S16 Fig Shrinkage and visual predictive check of the fits to the nasopharyngeal viral load data from severely infected patient cohort. (A) Parameter shrinkage. For each fit parameter (individual panels), the distribution of the population parameter (black line) and values sampled from the conditional distributions of the estimates of the individual parameters (histogram) are shown along with estimates of the shrinkage. Shrinkage = 1−(*var*(*η*)/*ω*^2^), where *ω* is the standard deviation of the random effect, and *var*(*η*) is the variance of the samples drawn from the conditional distributions of individual parameter estimates. (B) Visual predictive check. The blue segmented lines represent the trends of the observed data, and the blue and pink patches represent the trends of the model outputs generated via simulations. The lower, middle and upper blue lines represent the 10th, 50th and 90th percentile of the data, respectively. The patches indicated 90% confidence intervals for the median (middle), the 10th percentile (top) and the 90th percentile (bottom) of the simulations. Overall, the parameter shrinkages are low and the simulations correctly capture the variability in the data, indicating that the fits are good.
(TIF)

S17 Fig Quantifying uncertainties in individual fits in Fig 5B. The thin grey curves in each plot show model predictions using parameter combinations sampled from conditional distributions based on individual patient data fits. 50 realizations are presented for each patient. The error bars indicate standard deviations from these realizations. The bold curve in each plot is the prediction using the mode of the conditional distribution, as shown in Fig 5B in the main text. The open circles represent the data points. The patient IDs are the same as in Fig 5B and shown in boxes with numbers in purple fonts. The means and standard errors of the parameter values are listed in S11 Table.
(TIF)

S18 Fig Fits of the model with an alternative exhaustion formalism to viral load data from patients with severe symptoms. Best-fits of the model (S7 Text) (solid lines) to data (symbols) of nasopharyngeal viral load from patients with severe symptoms. Cross marks represent data points below the limit of detection. Entries in the boxes in purple fonts show patient IDs as in Fig 5B. The dashed lines reproduce the fits of the model in Fig 5B.
(TIF)

S19 Fig Robustness of model to antibody responses. Fits of our model to the data in Fig 5B (symbols) restricted to 15 days post symptom onset (solid lines) and projected to day 25 and beyond (dashed lines) compared to the fits in Fig 5B (dotted lines). Patient IDs are the same as in Fig 5B. The resulting population parameter estimates are in S13 Table.
(TIF)

S20 Fig The fits are not majorly affected upon reintroducing CD8 T cell exhaustion. We recalculated the dynamics in Fig 2 following the reintroduction of the CD8 T-cell exhaustion term using best-fit parameters for each patient and the chosen values of *k*_4_ and *k*_*e*_ (Methods). The panels and the quantities depicted are same as in Fig 2.
(TIF)

S21 Fig Sensitivity estimates of perturbations in the parameters associated with immunopathology. The relative difference in immunopathology between that corresponding to the population parameters estimated from mild and severe patient cohorts (see S8 Text), for different values of (A) α, (B) β, and (C) γ. α and β were varied from 0.1x to 10x of their default values, as indicated, whereas γ was varied from 0.5x to 1.5x of its default value.
(TIF)

S1 Table Events of exposure of the patients. Events of repeated exposure are indicated in the third column. Here ’0,1’ indicates that the individual was exposed on day 0 and on day 1.
(XLSX)

S2 Table Time of symptom onset in the patients. (XLSX)

S3 Table Dates of first measurements of viral loads in patients. (XLSX)

S4 TableSensitivity of the fit parameters to different initial values of $I_{0}^{*}$.(XLSX)

S5 Table Uncertainties in individual parameter estimates for fits in Fig 2. Means of parameter values (M) and their standard errors (SEM) for the parameters estimated from fits to individual patient data in Fig 2. 50 realizations of the model were used for these estimates of standard errors (see S4 Fig). (Note that the estimates in Table 3 contain the modes and not means of the parameter values.).
(XLSX)

S6 Table Estimated values of the population parameters of the candidate models. (XLSX)

S7 Table Values of the corrected Akaike information criterion (AICc) and the corrected Bayesian information criterion (BICc) for the candidate models. (XLSX)

S8 Table Individual model parameters estimated using the Wölfel *et al*. swab dataset. (XLSX)

S9 Table Uncertainties in individual parameter estimates for fits in Fig 5A. Means of parameter values (M) and their standard errors (SEM) for the parameters estimated from fits to individual patient data in Fig 5A. 50 realizations of the model were used for the SEM estimates (see S14 Fig). (Note that the estimates in Table S8 contain the modes and not means of the parameter values.).
(XLSX)

S10 Table Individual model parameters estimated from fits to the Néant *et al*. swab dataset. (XLSX)

S11 Table Uncertainties in individual parameter estimates for fits in Fig 5B. Means of parameter values (M) and their standard errors (SEM) for the parameters estimated from fits to individual patient data in Fig 5B. 50 realizations of the model were used for these estimates (see S17 Fig). (Note that the estimates in S10 Table contain the modes and not means of the parameter values.).
(XLSX)

S12 Table Population parameter estimates obtained from the fits of the model with the alternative exhaustion formalism (S18 Fig). (XLSX)

S13 Table Robustness of population parameter estimates to antibody responses. Comparison of population parameter estimates obtained by fitting our model to the Neant et al. dataset using data restricted to 15 days post symptom onset or using all the data.
(XLSX)

S1 Text Alternate procedures of calculation of immunopathology. (DOCX)

S2 Text Data collection and curation. (DOCX)

S3 Text Detailed description of model selection. (DOCX)

S4 Text Clearance is not stable in our model without CD8 T-cell response. (DOCX)

S5 Text Fixed points and their linear stability analysis. (DOCX)

S6 Text Effect of varying viral inoculum size. (DOCX)

S7 Text Model with an alternative formalism for CD8 T-cell exhaustion. (DOCX)

S8 Text Effect of perturbations in parameters associated with immunopathology. (DOCX)
