# Supplementary figures and images for: Modeling recapitulates the heterogeneous outcomes of SARS-CoV-2 infection and quantifies the differences in the innate immune and CD8 T-cell responses between patients experiencing mild and severe symptoms
Source: PLoS Pathog. 2022 Jun 27;18(6):e1010630. doi: 10.1371/journal.ppat.1010630 (PMC9269964; doi:10.1371/journal.ppat.1010630)

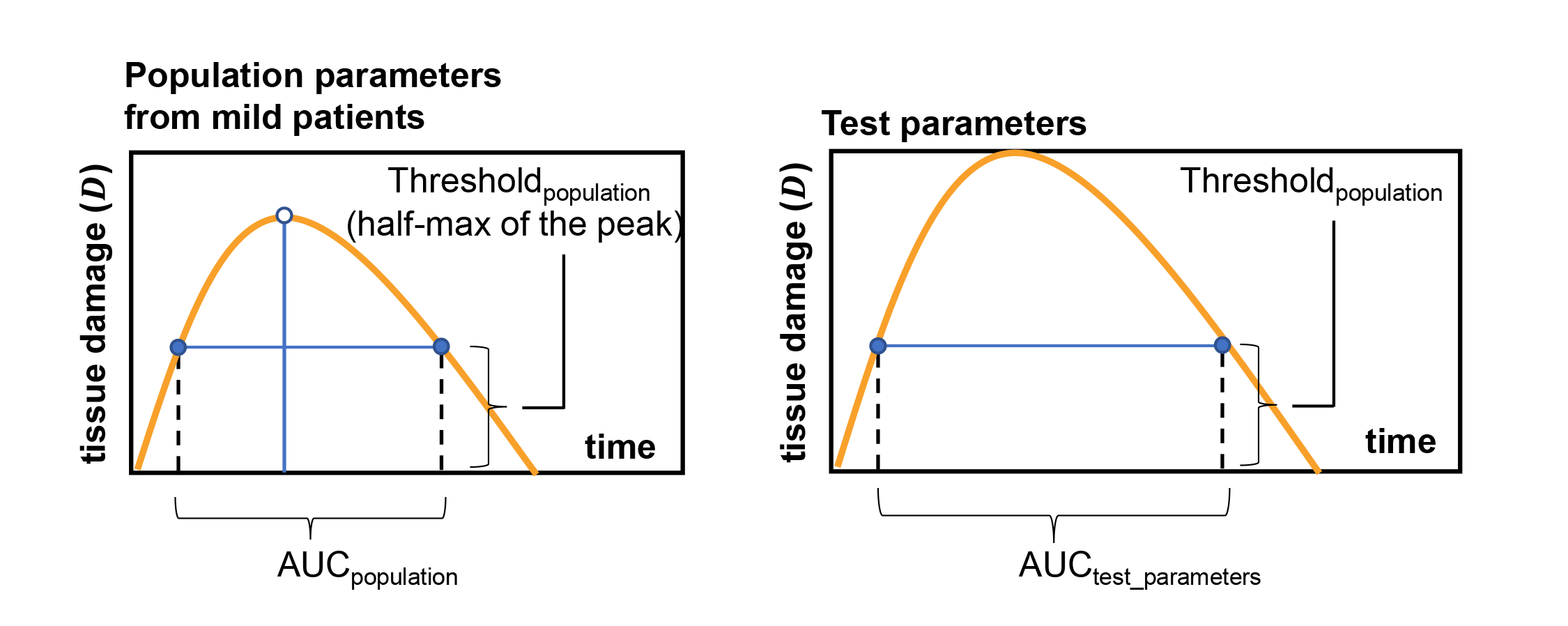

Supplement: S1 Fig — The peak of the instantaneous tissue damage (D) was detected for the simulation with the population parameters (left). A line parallel to the X-axis was drawn at the half-maximal level of D. The two intercepts of the curve of D with the horizontal line were identified. The area under the curve (AUC) was calculated within these half-maximal intercepts. The same threshold was used for parameters associated with an individual (right) and the AUC was calculated. The ratio of the latter AUC and the former was used as an estimate of the extent of immunopathology, P. Hence, immunopathology for model simulations with any parameter set is: Immunopathologytest_parameters = AUCtest_parameters/AUCpopulation_parameters. (TIF) [file ppat.1010630.s001.tif]

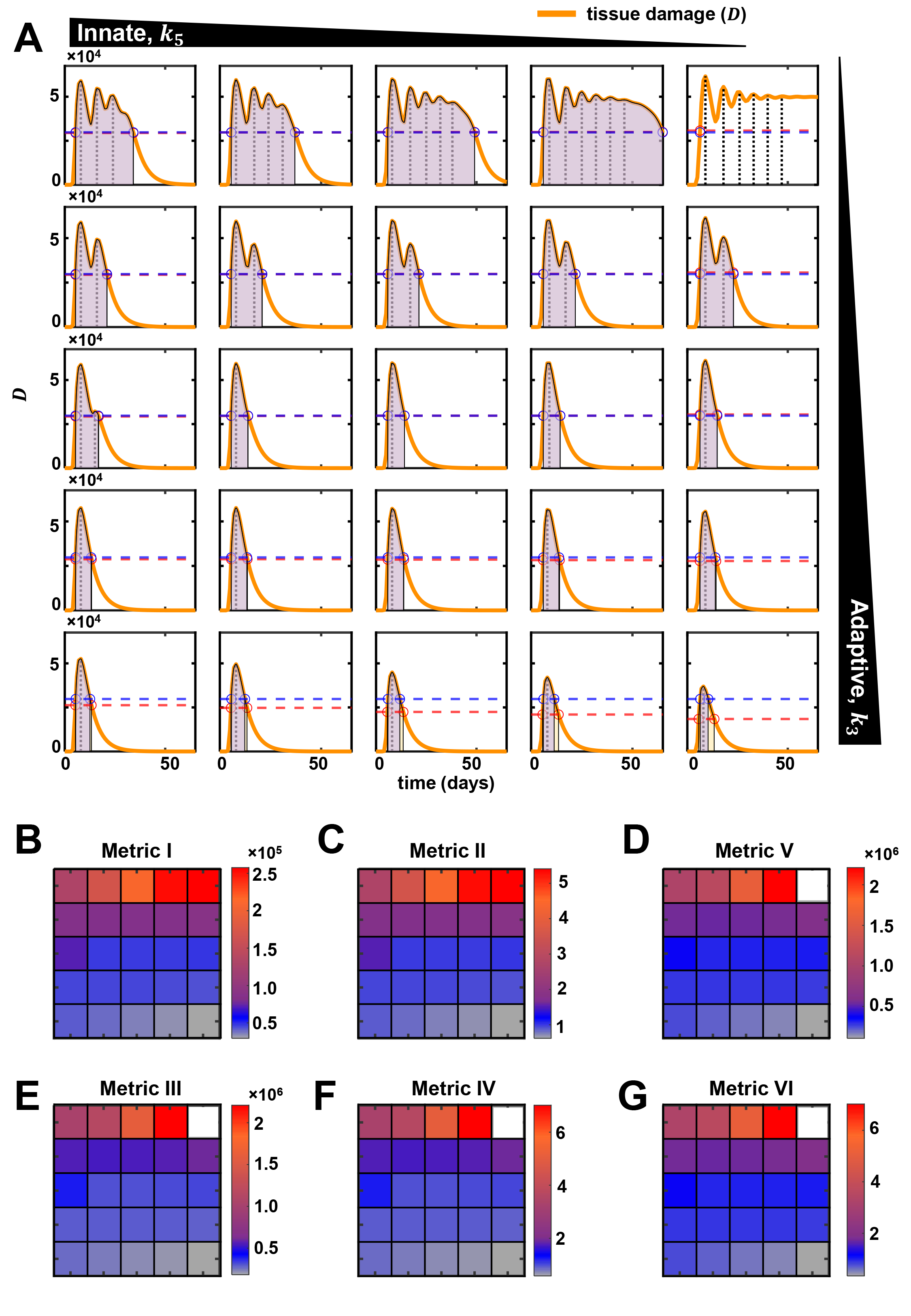

Supplement: S2 Fig — (A) The dynamical profiles of tissue damage (D) are shown for the simulations in Fig 4A. The black annotated triangles at the top and right indicate the nature and the direction of the variation of the indicated parameters. Vertical dotted lines are peaks in D. The blue horizontal dashed line represents the common threshold, as indicated in metric V (S1 Text), and the purple shaded region is its AUC. The red dashed line represents the threshold calculated following metric III, and the light orange shaded area its AUC. (B)-(G) Colour maps in 5×5 grids represent the immunopathology scores calculated for the subplots shown in (A) following different metrics (see S1 Text). The empty grid represents diverging immunopathology (see Fig 4). (TIF) [file ppat.1010630.s002.tif]

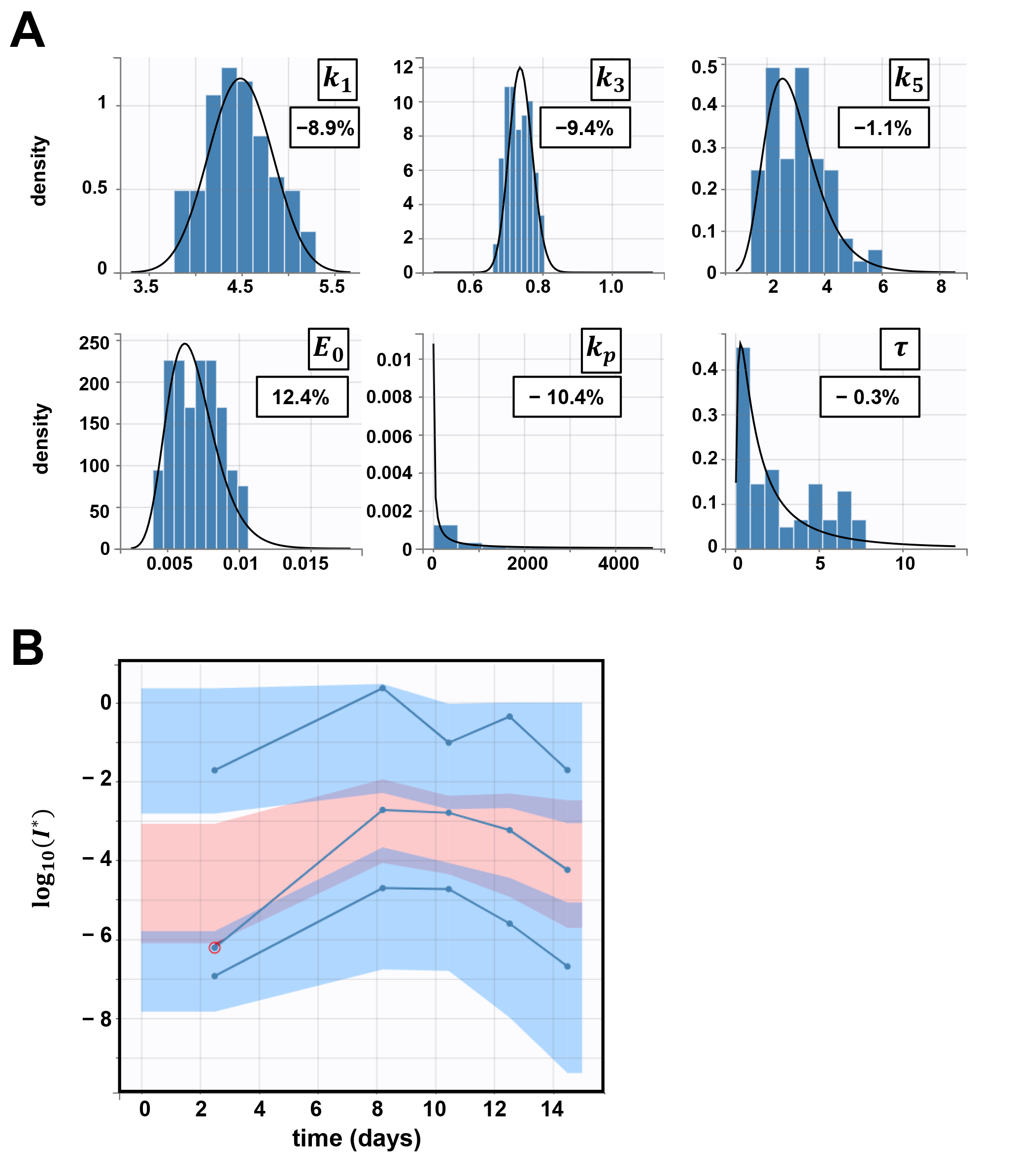

Supplement: S3 Fig — (A) Parameter shrinkage. For each fit parameter (individual panels), the distribution of the population parameter (black line) and values sampled from the conditional distributions of the estimates of the individual parameters (histogram) are shown along with estimates of the shrinkage. Shrinkage = 1−(var(η)/ω2), where ω is the standard deviation of the random effect, and var(η) is the variance of the samples drawn from the conditional distributions of individual parameter estimates. (B) Visual predictive check. The blue segmented lines represent the trends of the observed data, and the blue and pink patches represent the trends of the model outputs generated via simulations. The lower, middle and upper blue lines represent the 10th, 50th and 90th percentile of the data, respectively. The patches indicated 90% confidence intervals for the median (middle), the 10th percentile (top) and the 90th percentile (bottom) of the simulations. Overall, the parameter shrinkages are low and the simulations correctly capture the variability in the data, indicating that the fits are good. (TIF) [file ppat.1010630.s003.tif]

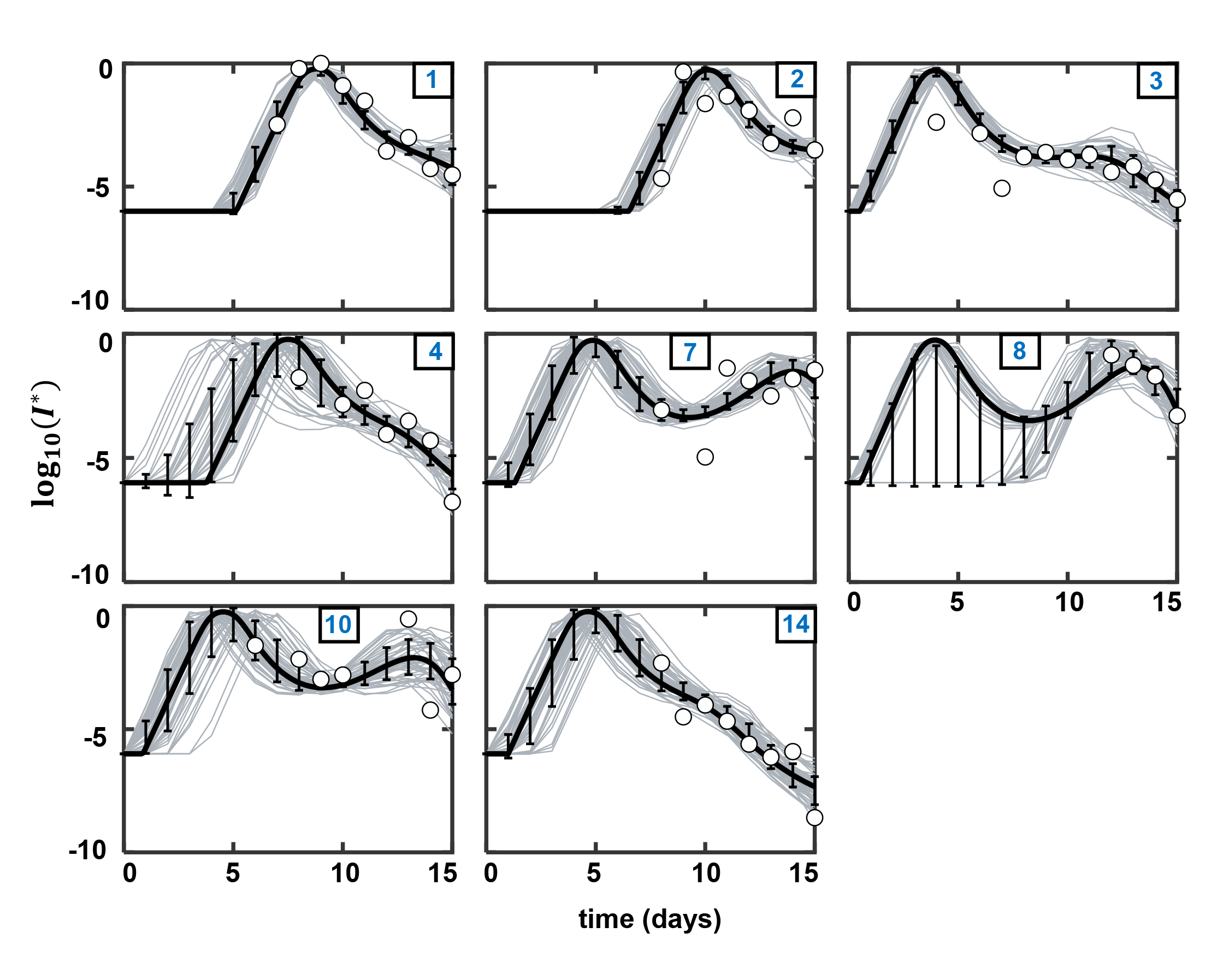

Supplement: S4 Fig — The thin grey curves in each plot show model predictions using parameter combinations sampled from conditional distributions based on individual patient data fits. 50 realizations are presented for each patient. The error bars indicate standard deviations from these realizations. The bold curve in each plot is the prediction using the mode of the conditional distribution, as shown in Fig 2 in the main text. The open circles represent the data points. The patient IDs are the same as in Fig 2, and shown in boxes with blue numbers. The means and standard errors of the parameter values are listed in S5 Table. (TIF) [file ppat.1010630.s004.tif]

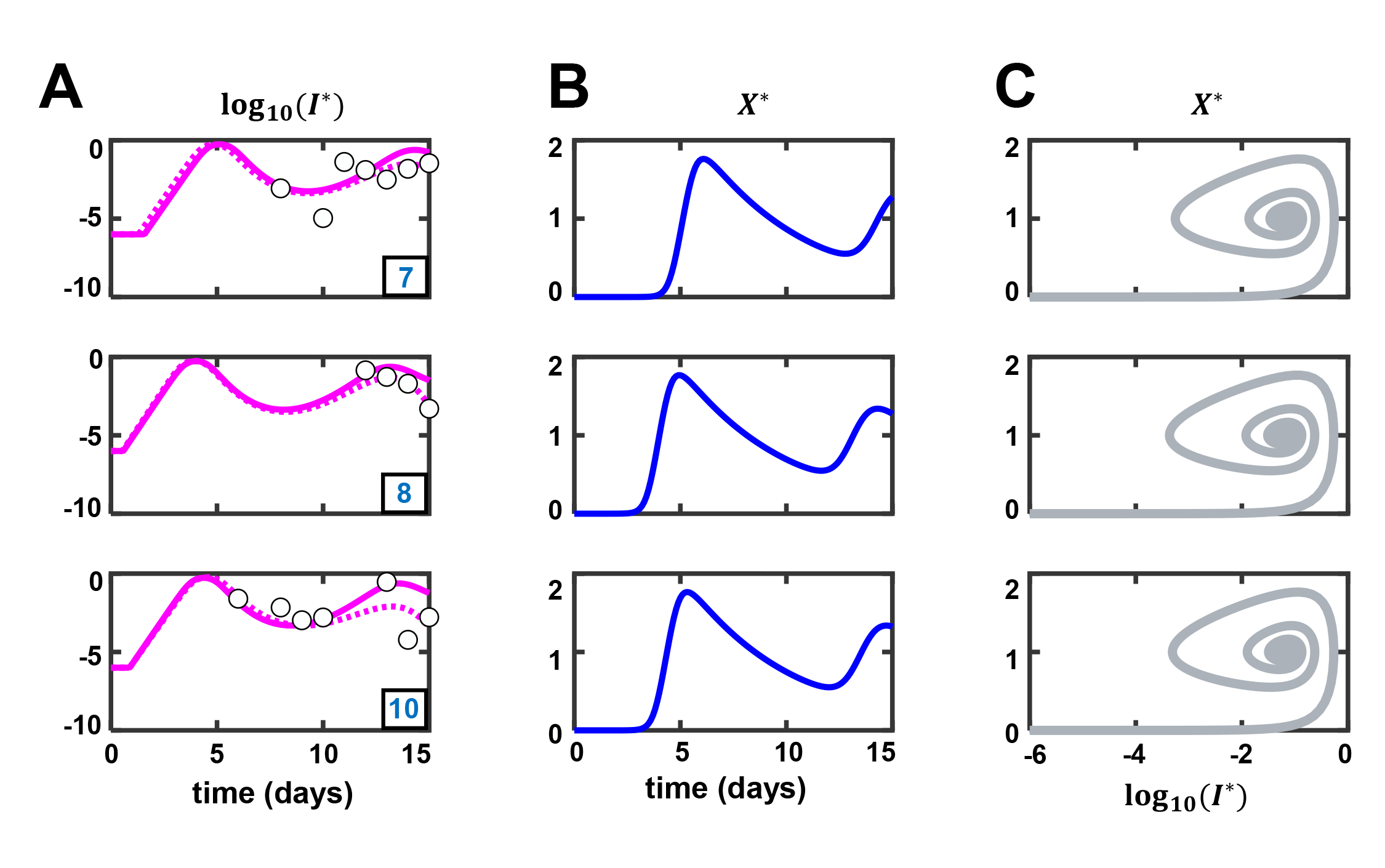

Supplement: S5 Fig — Fits (solid lines in panel A) of our model (Eqs 5–7) with E0*=0 to data (symbols) from patients 7, 8, and 10. Fits from Fig 2 are reproduced for comparison (dashed lines). Corresponding predictions of the innate immune response (panel C), and the associated phase plane plots indicating prolonged oscillations. Best-fit parameter estimates for the three patients were as follows. Patient 7: k1 = 4.49 days-1, k5*=2.98 days-1 and τ = 1.58 days; Patient 8: k1 = 4.62 days-1, k5*=3.17 days-1 and τ = 0.58 days; and Patient 10: k1 = 4.54 days-1, k5*=3.11 days-1 and τ = 0.88 days. (TIF) [file ppat.1010630.s005.tif]

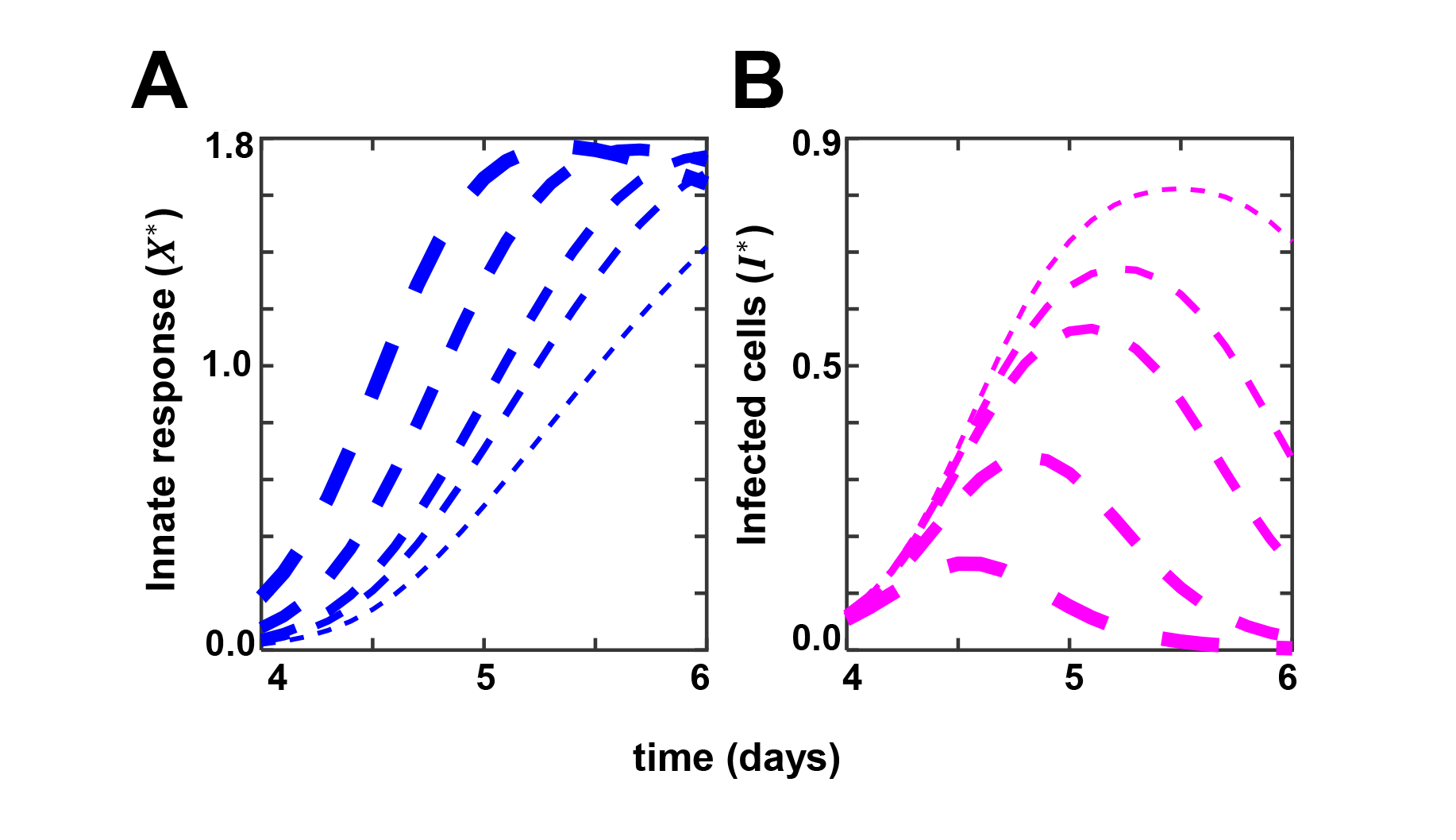

Supplement: S6 Fig — The width of the curves is proportional to the strength of k5. (A) and (B) represent the dynamics of X and I, respectively. The population estimates (fixed effects) of the parameters estimated in Fig 2 (Table 2) were used. Parameter values used: k1 = 4.49/day, k3 = 0.74/day, k5*=2.83/day, E0*=6.65×10^−3^/day, kp*=2.497×10^−4^,τ=1.51 day, k6 = 0.2/day, k4 = 1.5/day, ke*=0.7, α = 1.0×10^4^, β = 2.0×10^4^/day, γ = 0.5/day. The fold-changes for variation in k5* are: 0.5, 0.75, 1, 2, 5. (TIF) [file ppat.1010630.s006.tif]

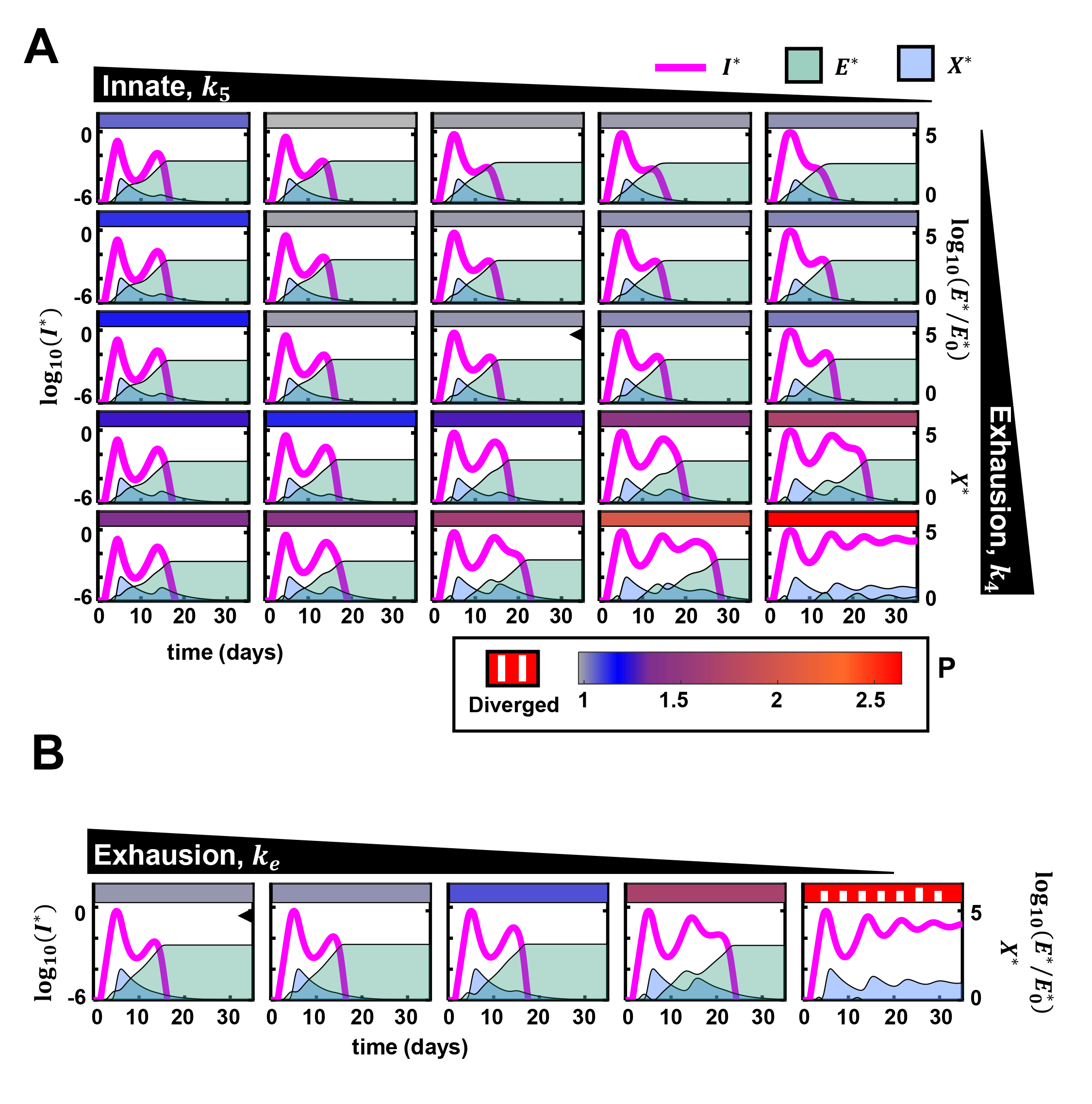

Supplement: S7 Fig — (A) Effect of simultaneous variation of parameters determining the strengths of innate response and CD8 T-cell exhaustion on the trajectory of the infection is shown. The black annotated triangles at the right and the top depict the nature and the direction of the variation of the indicated parameters. For instance, k4 increases top to bottom and k5 is increases from right to left. Individual subplots show the dynamics of infected cells, effector CD8 T-cell response and innate immune response. Each subplot is a double Y-axis plot. The left Y-axis shows the normalized infected cell dynamics. The right Y-axis shows the other two species. The colored patches at the top of the subplots represent the extent of immunopathology. The range of immunopathology is given by the color scale at the bottom. On the left-side of the color scale, a separate legend denotes the texture used for depicting unbounded immunopathology (see text). Unity on the colorscale indicates the immunopathology quantified in the central subplot (subplot with an arrowhead), calculated using the population parameters estimated from Fig 2. (Table 2, see text). The population estimates (fixed effects) of the parameters estimated in Fig 2 (Table 2) were used. Parameter values used: k1 = 4.49/day, k3 = 0.74/day, k5*=2.83/day, E0*=6.65×10^−3^/day, kp*=2.497×10^−4^,τ=1.51 day, k6 = 0.2/day, k4 = 1.5/day, ke*=0.7,α=1.0×10^4^,β=2.0×10^4^/day, γ = 0.5/day. Variations in k4 are obtained as the following fold-changes to the above value: 0.35, 0.75, 1, 2, 3. The fold-changes for variation in k5* are: 0.5, 0.75, 1, 2, 5. Values of ke* used in (B) are: 0.01, 0.1, 0.3, 0.5, 0.7. (TIF) [file ppat.1010630.s007.tif]

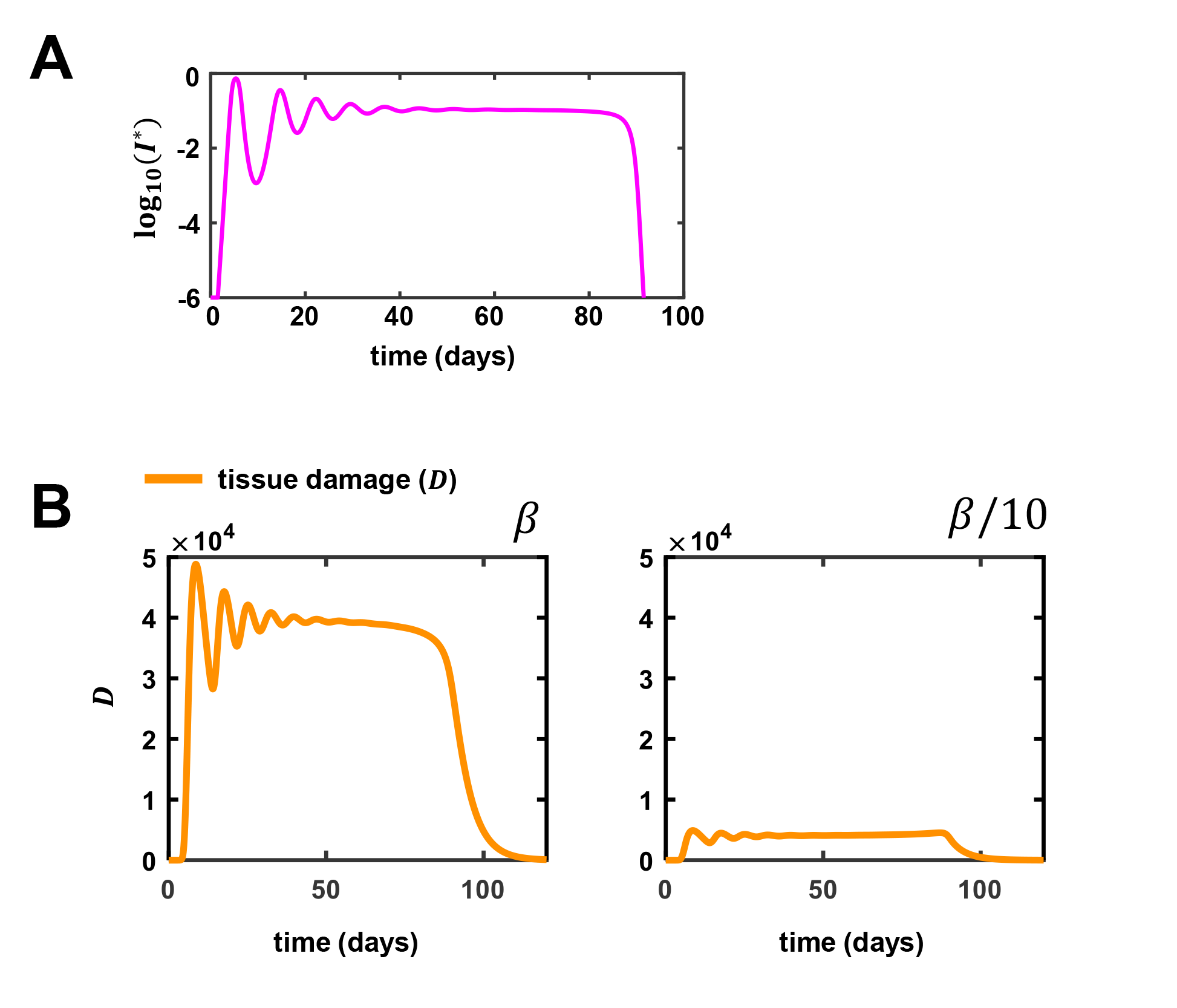

Supplement: S8 Fig — (A) A model simulation with long-duration infection is depicted. Tuning the parameters k5, ke, and k4 allowed realization of the long-duration infection scenarios. (B) Tissue damage profiles with the nominal value of β (left) and a ten-fold lower value (right). Note that in the right panel, the initial peak of tissue damage is 10% of that in the left panel. (TIF) [file ppat.1010630.s008.tif]

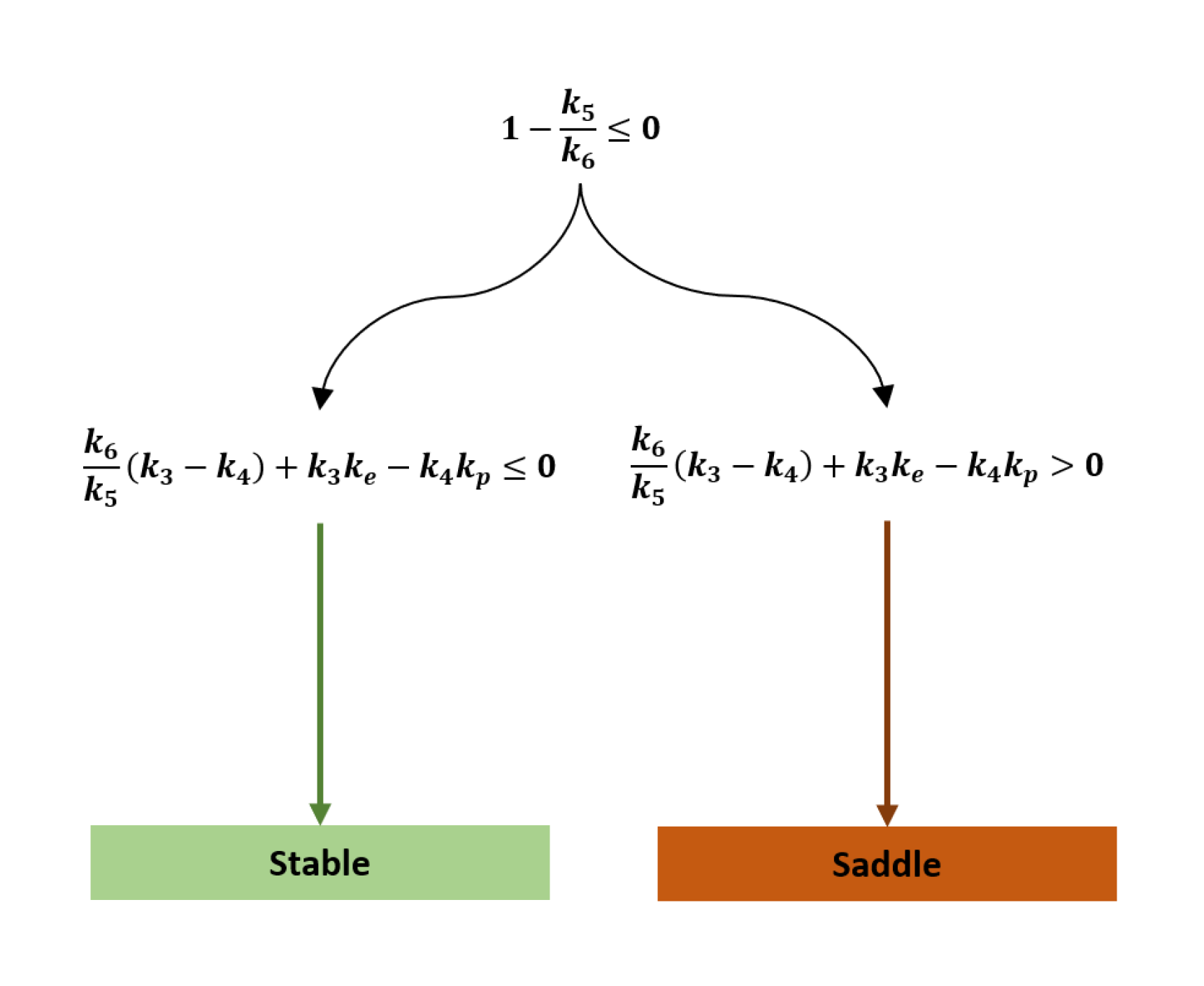

Supplement: S9 Fig — See S5 Text for a description of the parameter regimes and stability criteria. (TIF) [file ppat.1010630.s009.tif]

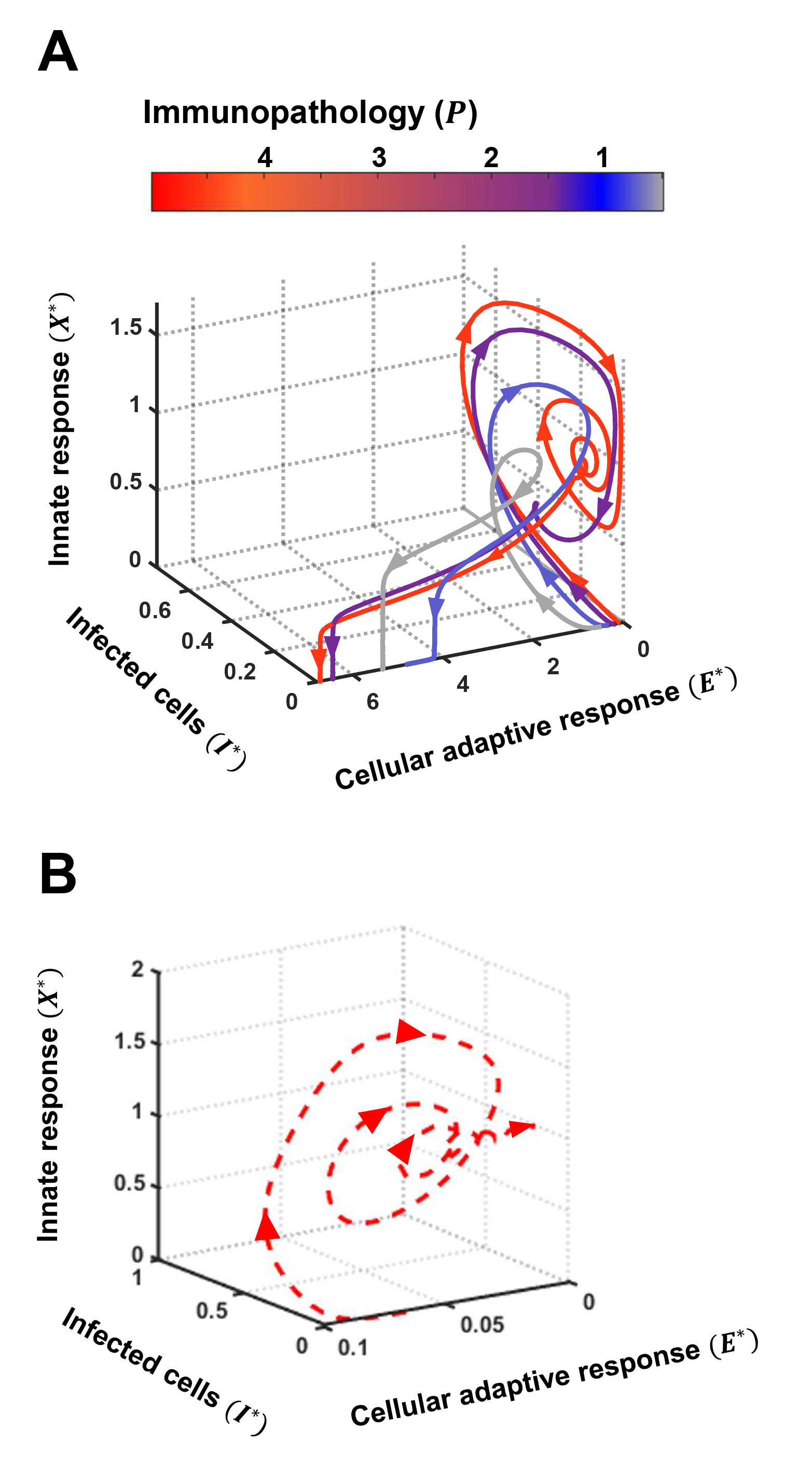

Supplement: S10 Fig — (A) Trajectories in 3D space defined by infected cells, CD8 T-cells and cytokine-mediated innate response for parameter combinations where clearance alone is a stable fixed point. Each trajectory uses different initial conditions. The colors of the trajectories represent the immunopathology associated, defined in the scale bar at the top. Immunopathology corresponding to population parameter estimates (Table 2) is represented by unity on the color scale. (B) Dashed red line is a trajectory headed towards fixed point 2. For such trajectories, immunopathology was typically unbounded in our model. (TIF) [file ppat.1010630.s010.tif]

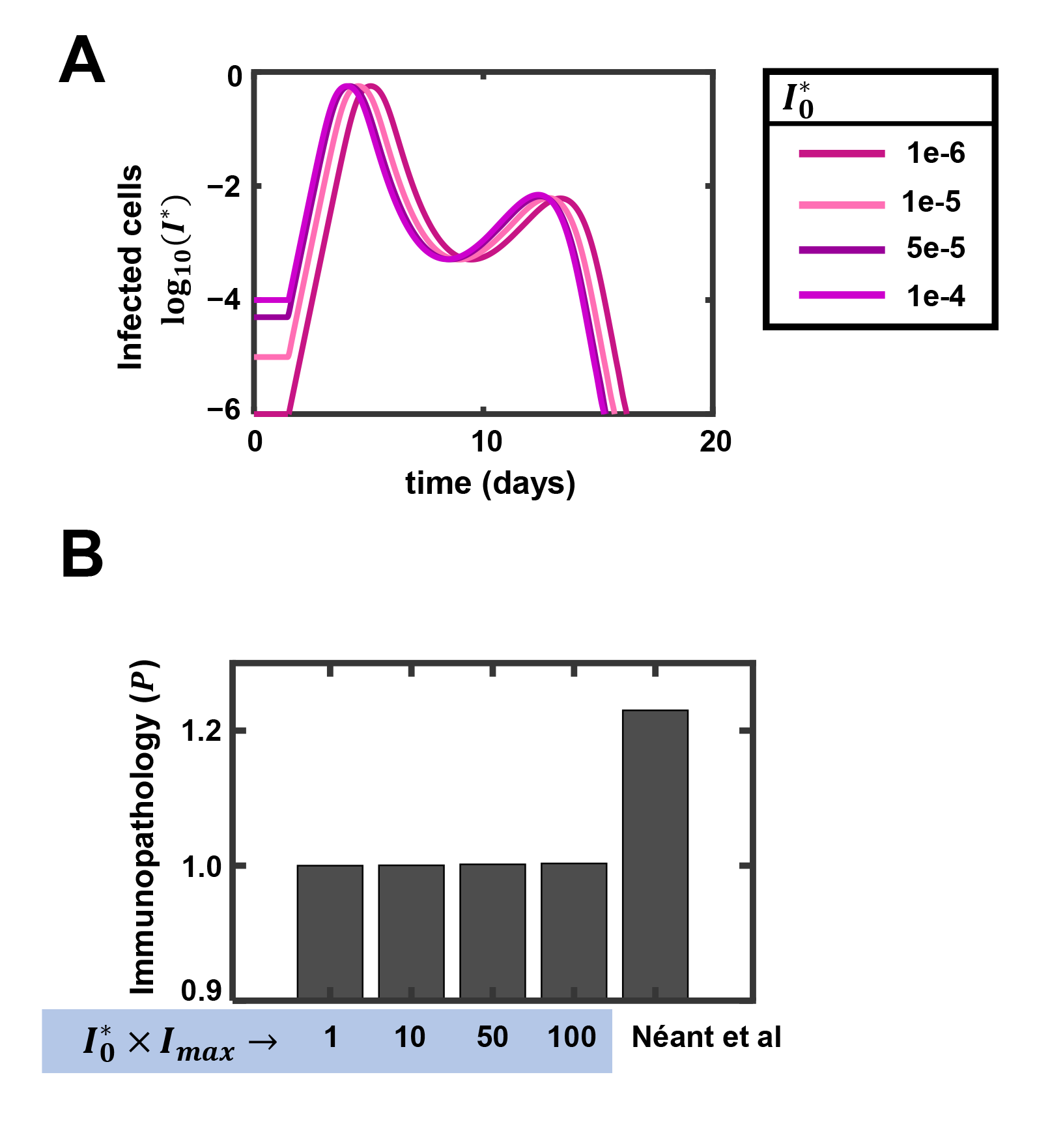

Supplement: S11 Fig — (A) Infected cells dynamics with varying initial infected cell pool sizes (1, 10, 50, 100 cells). (B) Immunopathology of the 4 trajectories compared with the calculated immunopathology for parameters representing severely infected patients (Table 5). (TIF) [file ppat.1010630.s011.tif]

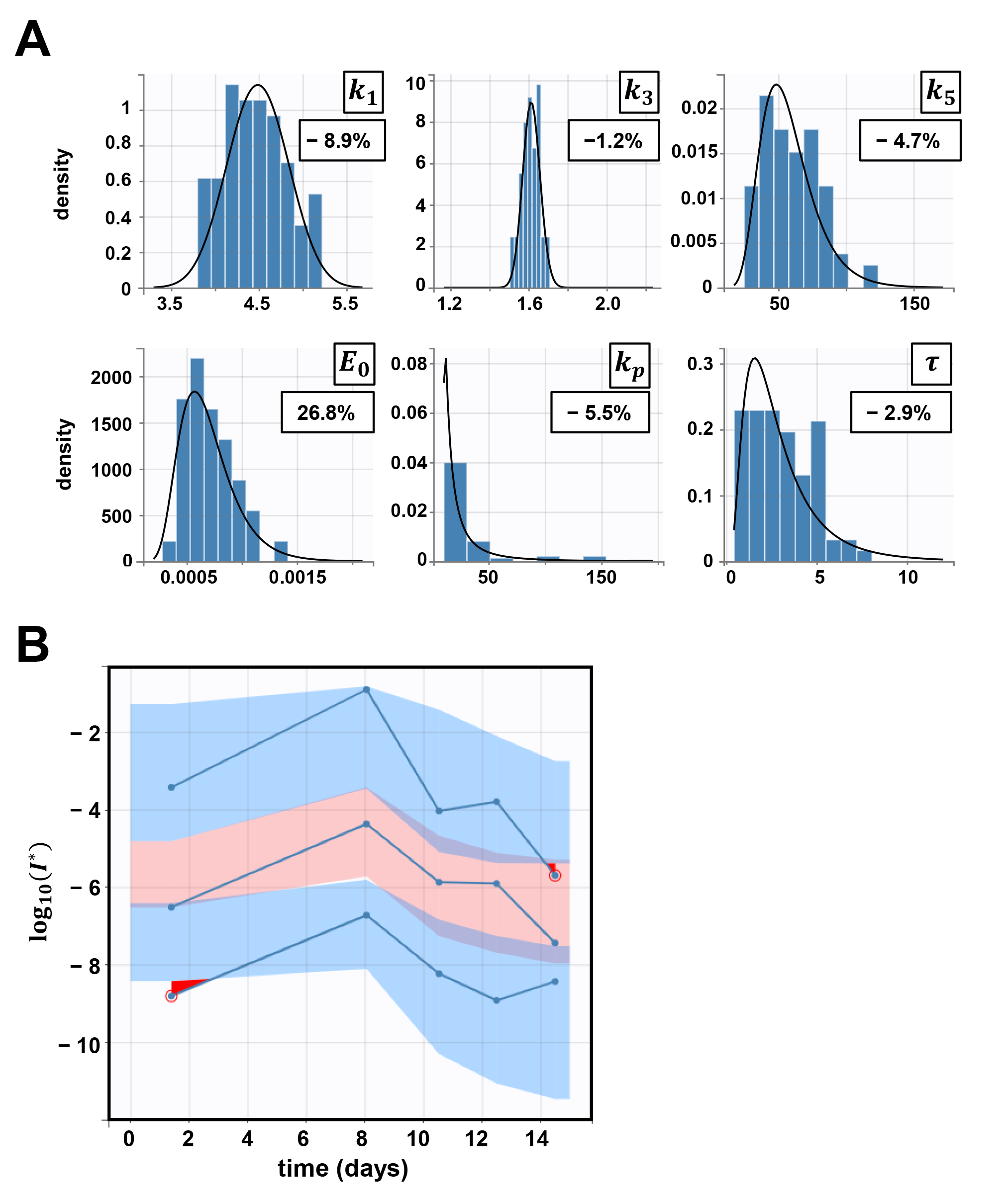

Supplement: S12 Fig — (A) Parameter shrinkage. For each fit parameter (individual panels), the distribution of the population parameter (black line) and values sampled from the conditional distributions of the estimates of the individual parameters (histogram) are shown along with estimates of the shrinkage. Shrinkage = 1−(var(η)/ω2), where ω is the standard deviation of the random effect, and var(η) is the variance of the samples drawn from the conditional distributions of individual parameter estimates. (B) Visual predictive check. The blue segmented lines represent the trends of the observed data, and the blue and pink patches represent the trends of the model outputs generated via simulations. The lower, middle and upper blue lines represent the 10th, 50th and 90th percentile of the data, respectively. The patches indicated 90% confidence intervals for the median (middle), the 10th percentile (top) and the 90th percentile (bottom) of the simulations. Overall, the parameter shrinkages are low and the simulations correctly capture the variability in the data, indicating that the fits are good. (TIF) [file ppat.1010630.s012.tif]

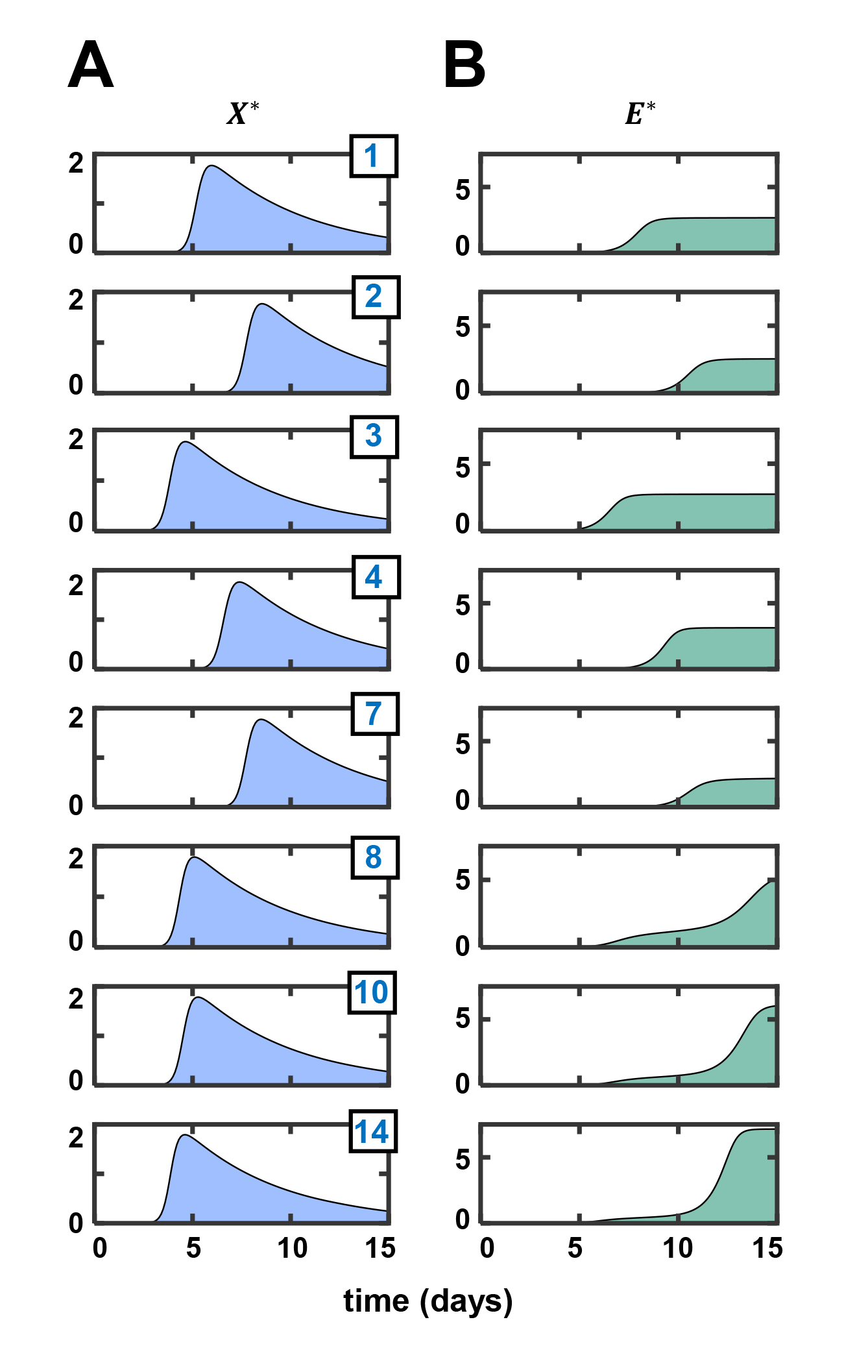

Supplement: S13 Fig — (A) The numbers in the boxes on the top of each plot represents the patient IDs as provided in Bӧhmer et al. [37]. The blue area plots represent the predicted innate immune response corresponding to the infection dynamics shown in Fig 5A in the main text. (B) The green area plots show the predicted dynamics of CD8 T-cell mediated adaptive immune response. (TIF) [file ppat.1010630.s013.tif]

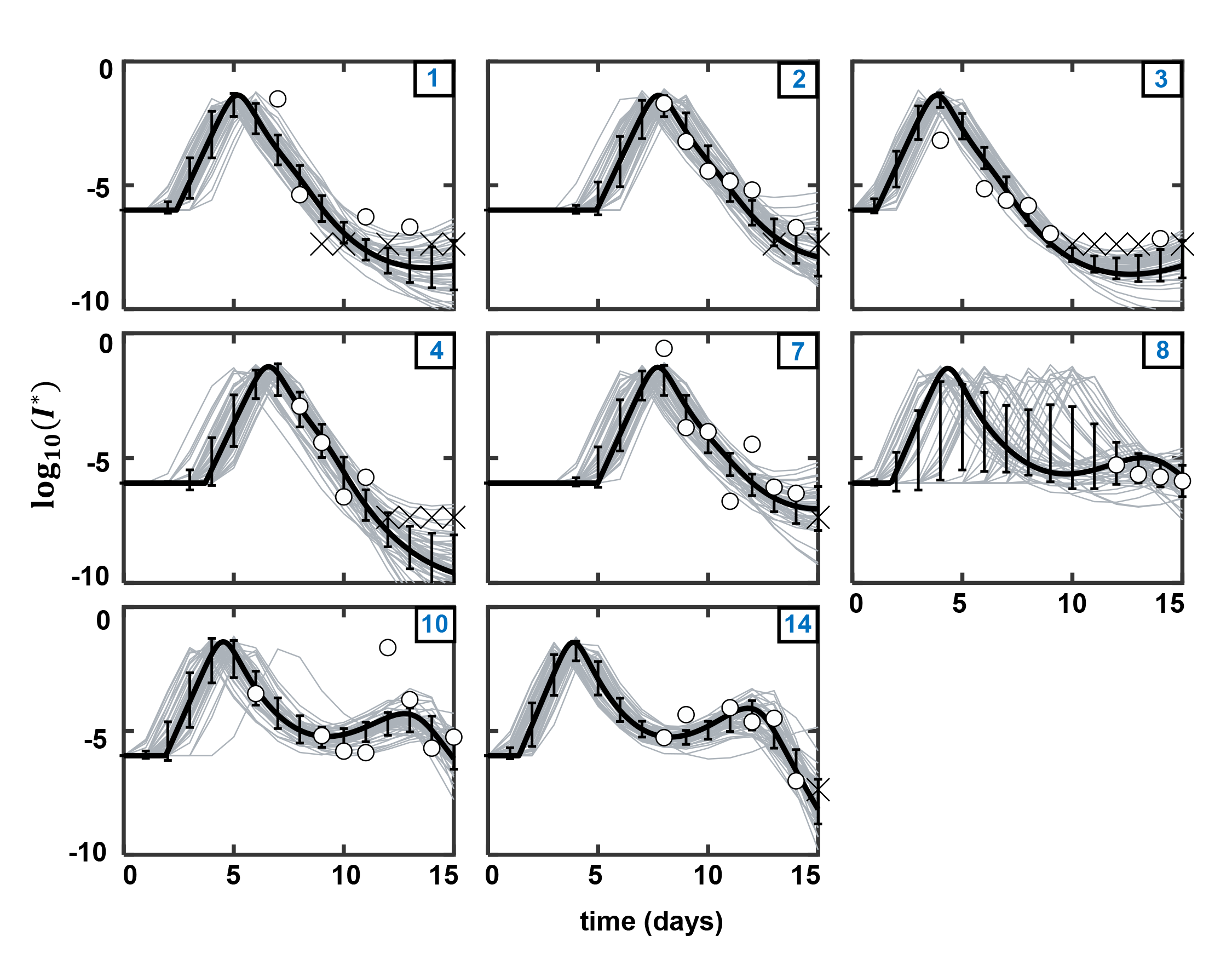

Supplement: S14 Fig — The thin grey curves in each plot show model predictions using parameter combinations sampled from conditional distributions based on individual patient data fits. 50 realizations are presented for each patient. The error bars indicate standard deviations from these realizations. The bold curve in each plot is the prediction using the mode of the conditional distribution, as shown in Fig 5A in the main text. The open circles represent the data points, and crosses represent data points below detection limit. The patient IDs are the same as in Fig 5A, and shown in boxes with numbers in blue. The means and standard errors of the parameter values are listed in S9 Table. (TIF) [file ppat.1010630.s014.tif]

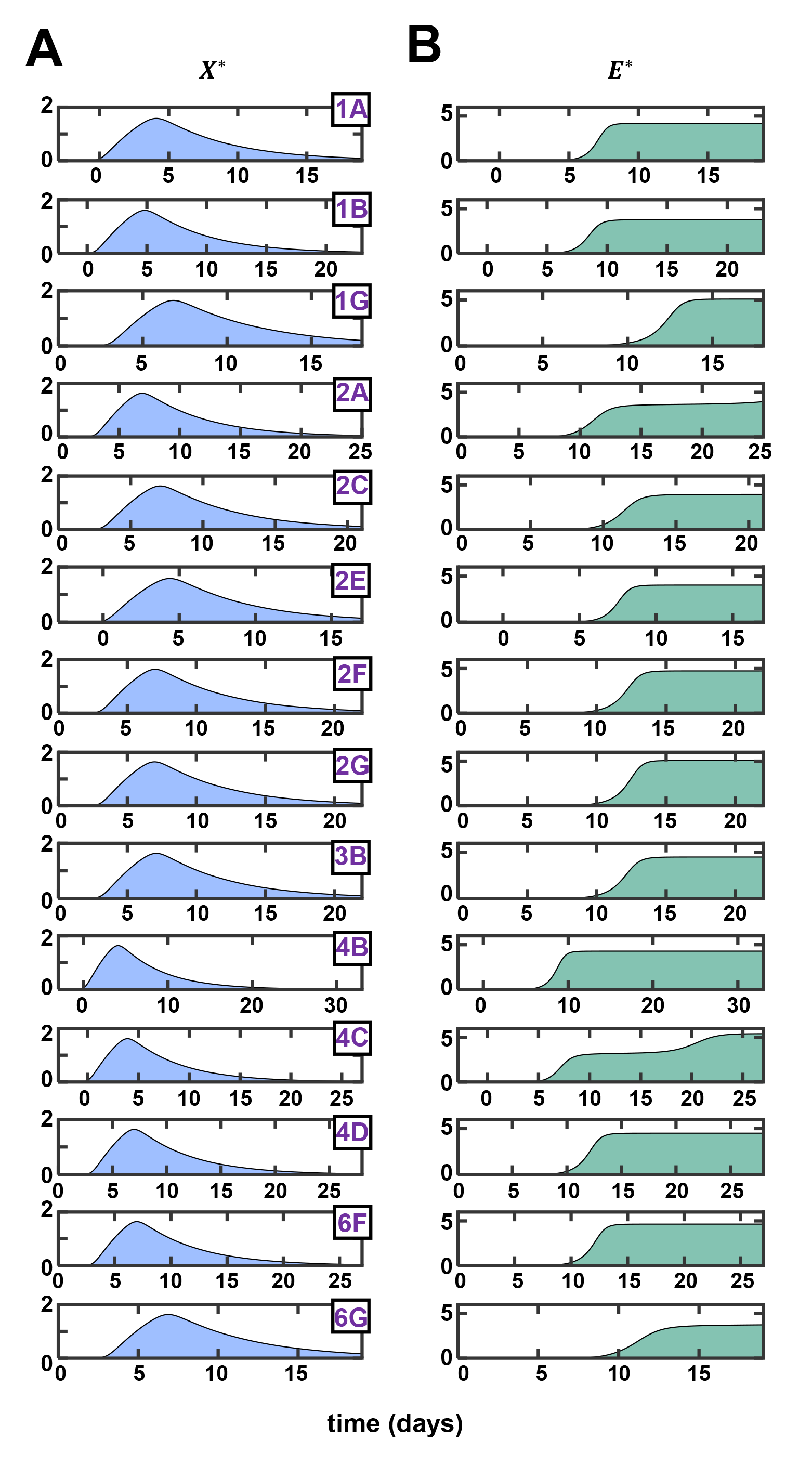

Supplement: S15 Fig — (A) The blue area plots represent the predicted innate immune response corresponding to the infection dynamics shown in Fig 5B in the main text. The alpha numeric entries in the boxes indicate the position of the plots in the original figure in Neant et al. [60] (see Fig 5B). X-axes represent time in days, post symptom onset. (B) The green area plots show the predicted dynamics of CD8 T-cell mediated adaptive immune response. (TIF) [file ppat.1010630.s015.tif]

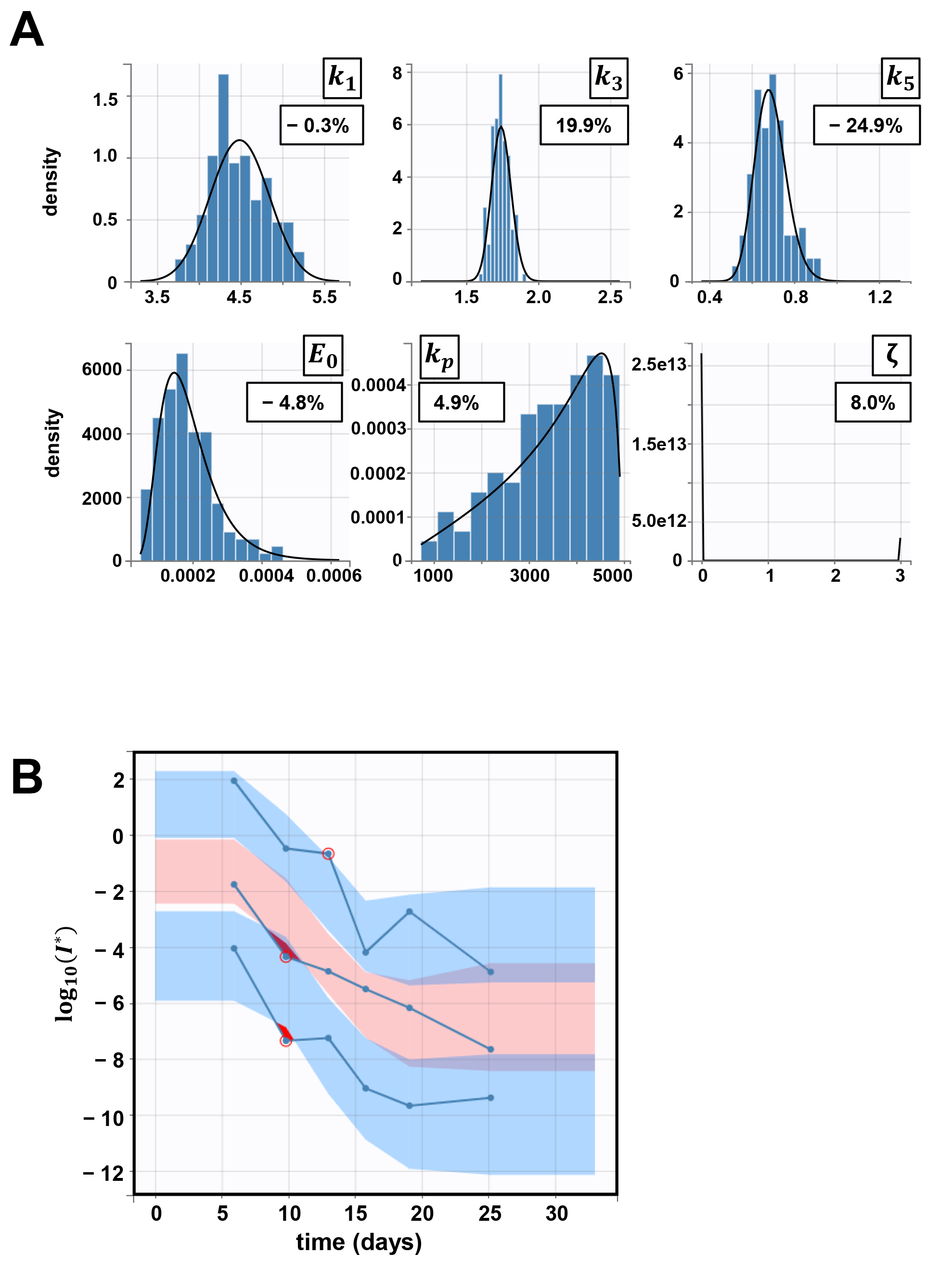

Supplement: S16 Fig — (A) Parameter shrinkage. For each fit parameter (individual panels), the distribution of the population parameter (black line) and values sampled from the conditional distributions of the estimates of the individual parameters (histogram) are shown along with estimates of the shrinkage. Shrinkage = 1−(var(η)/ω2), where ω is the standard deviation of the random effect, and var(η) is the variance of the samples drawn from the conditional distributions of individual parameter estimates. (B) Visual predictive check. The blue segmented lines represent the trends of the observed data, and the blue and pink patches represent the trends of the model outputs generated via simulations. The lower, middle and upper blue lines represent the 10th, 50th and 90th percentile of the data, respectively. The patches indicated 90% confidence intervals for the median (middle), the 10th percentile (top) and the 90th percentile (bottom) of the simulations. Overall, the parameter shrinkages are low and the simulations correctly capture the variability in the data, indicating that the fits are good. (TIF) [file ppat.1010630.s016.tif]

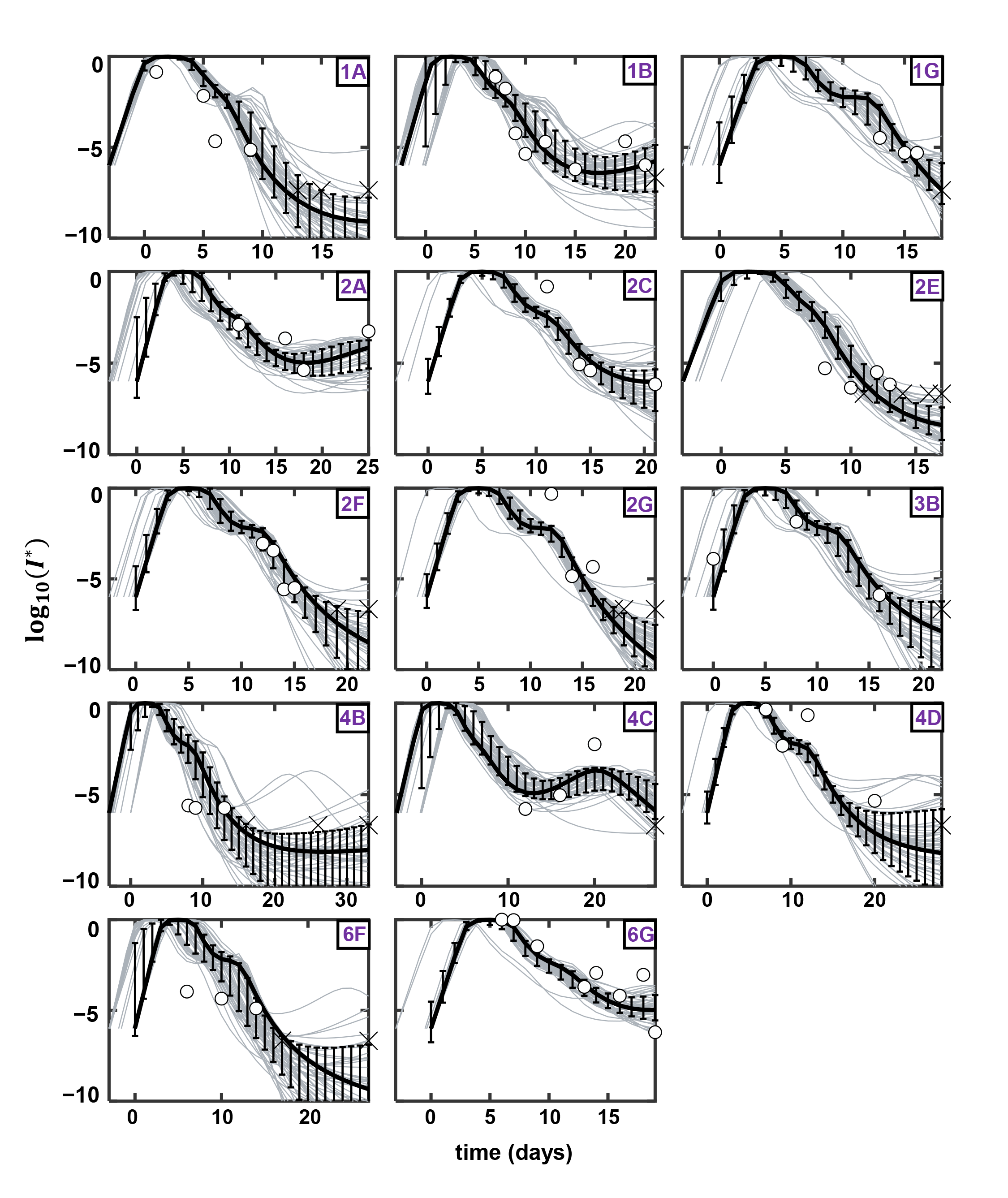

Supplement: S17 Fig — The thin grey curves in each plot show model predictions using parameter combinations sampled from conditional distributions based on individual patient data fits. 50 realizations are presented for each patient. The error bars indicate standard deviations from these realizations. The bold curve in each plot is the prediction using the mode of the conditional distribution, as shown in Fig 5B in the main text. The open circles represent the data points. The patient IDs are the same as in Fig 5B and shown in boxes with numbers in purple fonts. The means and standard errors of the parameter values are listed in S11 Table. (TIF) [file ppat.1010630.s017.tif]

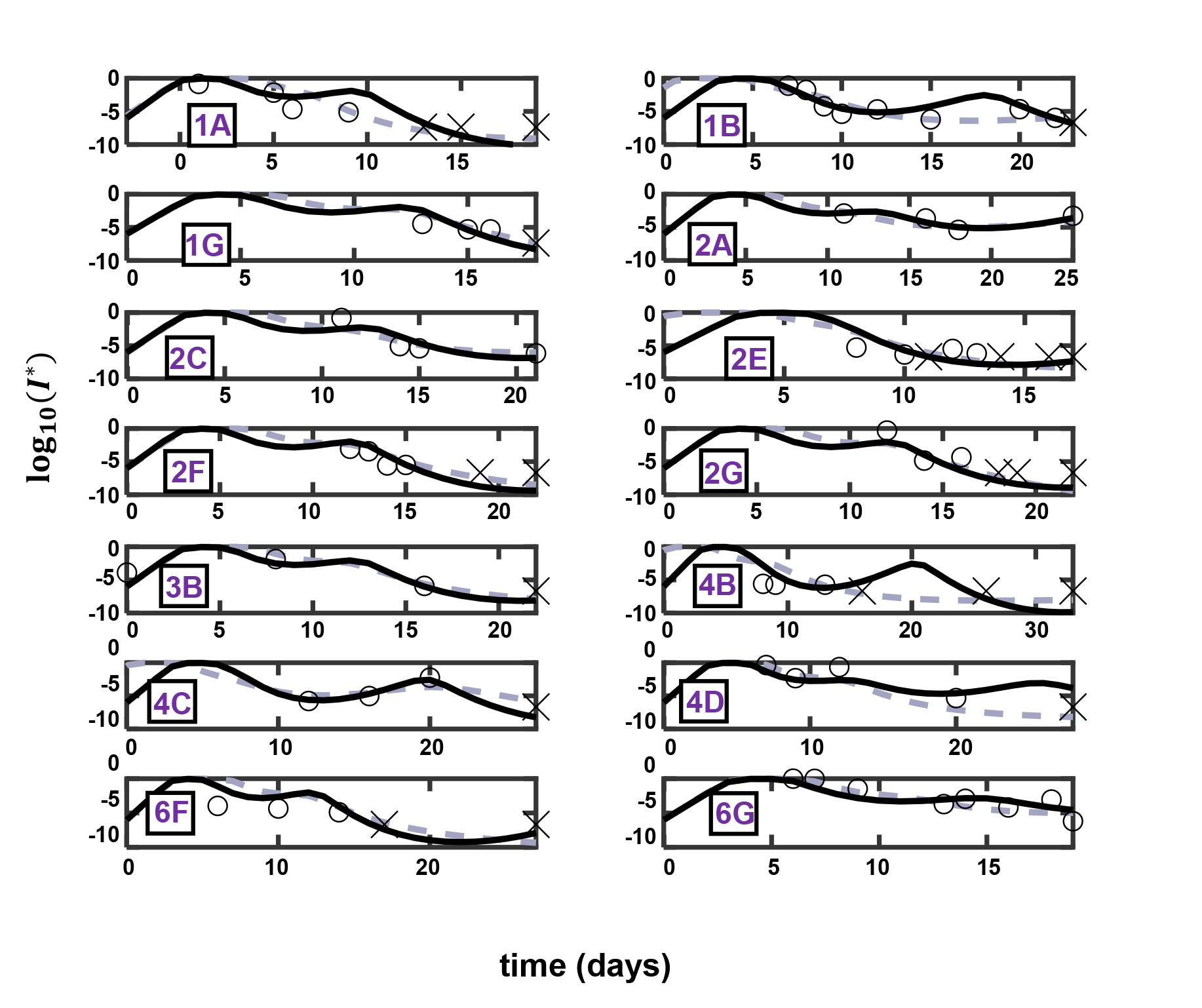

Supplement: S18 Fig — Best-fits of the model (S7 Text) (solid lines) to data (symbols) of nasopharyngeal viral load from patients with severe symptoms. Cross marks represent data points below the limit of detection. Entries in the boxes in purple fonts show patient IDs as in Fig 5B. The dashed lines reproduce the fits of the model in Fig 5B. (TIF) [file ppat.1010630.s018.tif]

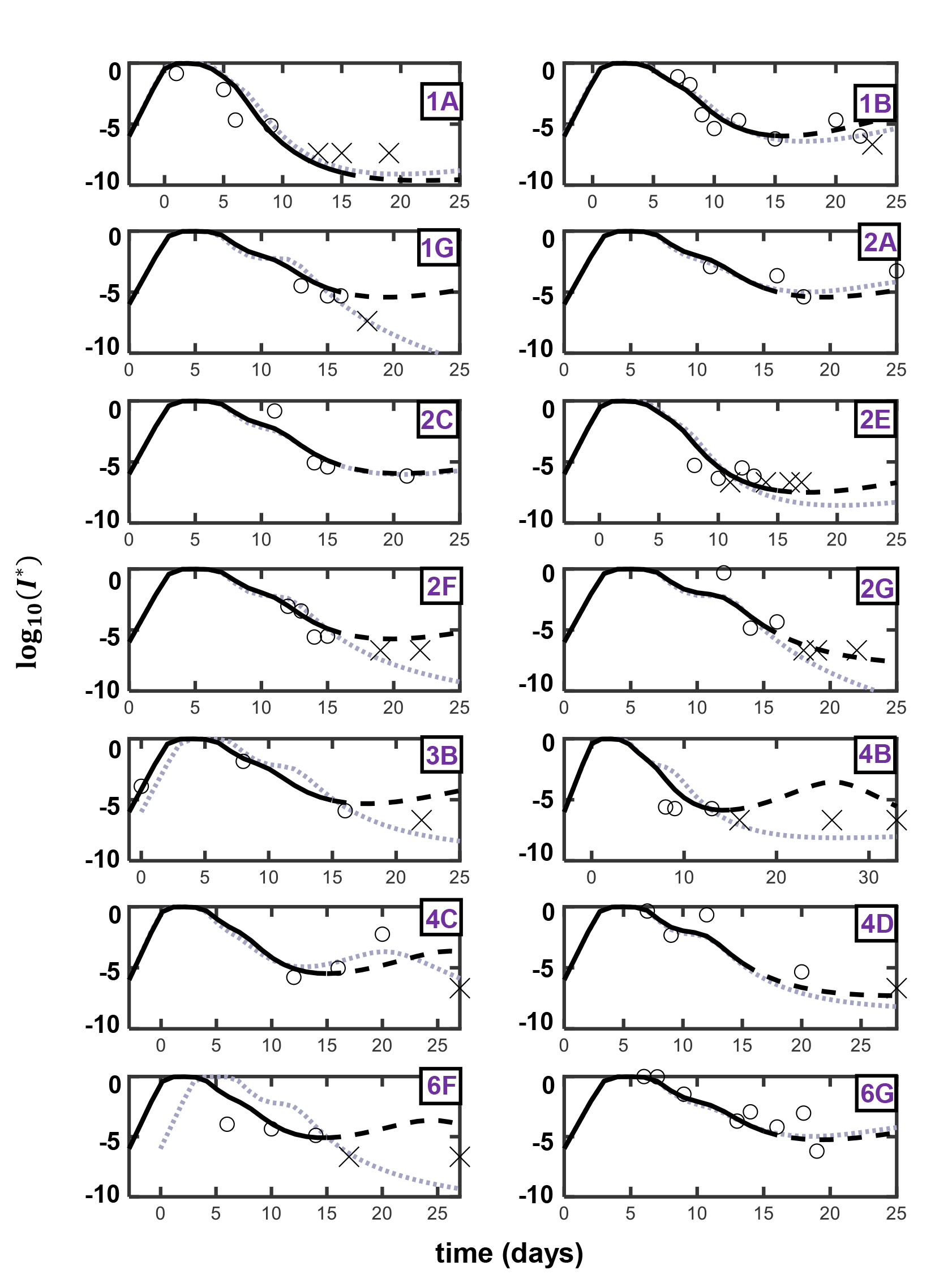

Supplement: S19 Fig — Fits of our model to the data in Fig 5B (symbols) restricted to 15 days post symptom onset (solid lines) and projected to day 25 and beyond (dashed lines) compared to the fits in Fig 5B (dotted lines). Patient IDs are the same as in Fig 5B. The resulting population parameter estimates are in S13 Table. (TIF) [file ppat.1010630.s019.tif]

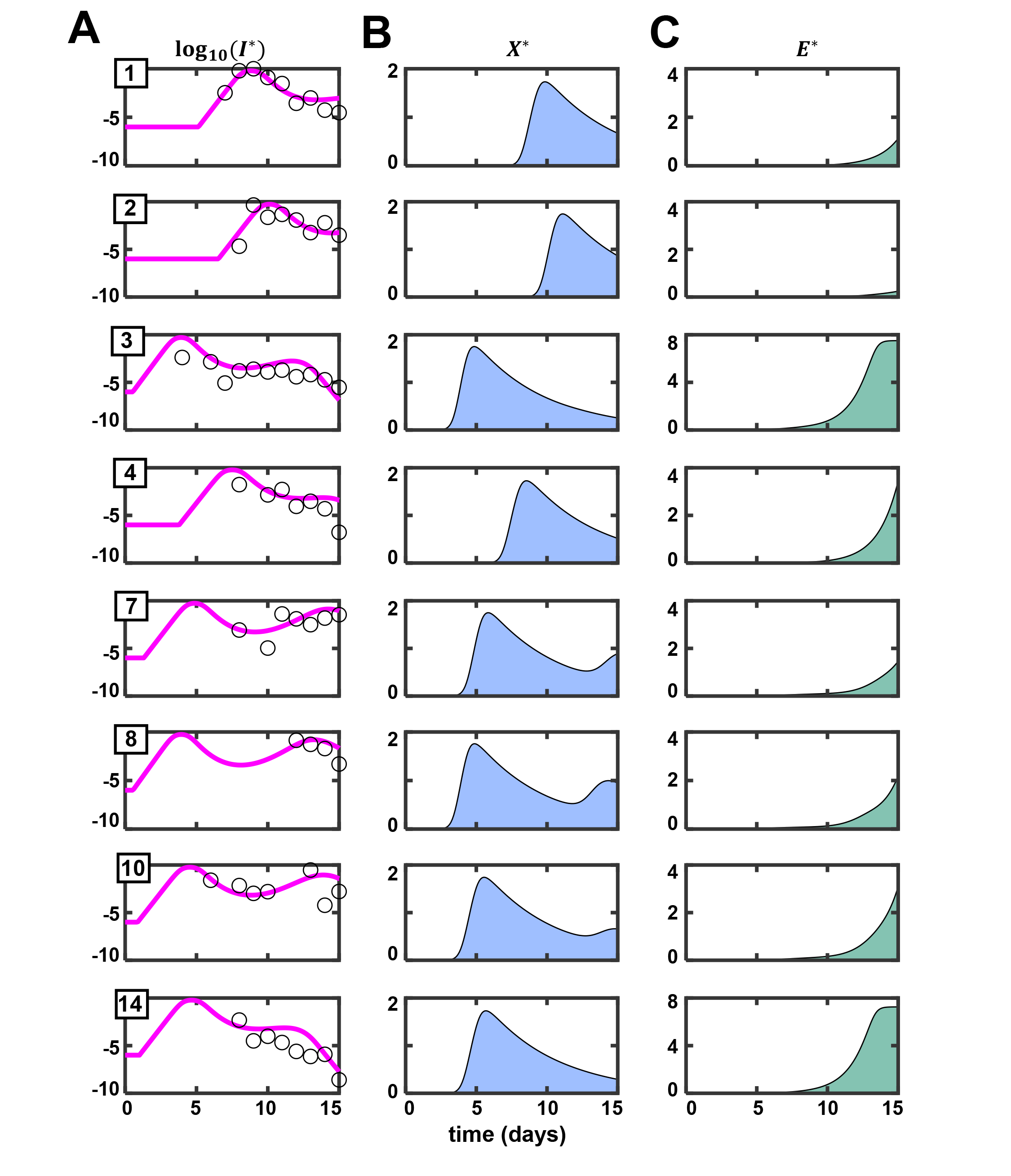

Supplement: S20 Fig — We recalculated the dynamics in Fig 2 following the reintroduction of the CD8 T-cell exhaustion term using best-fit parameters for each patient and the chosen values of k4 and ke (Methods). The panels and the quantities depicted are same as in Fig 2. (TIF) [file ppat.1010630.s020.tif]

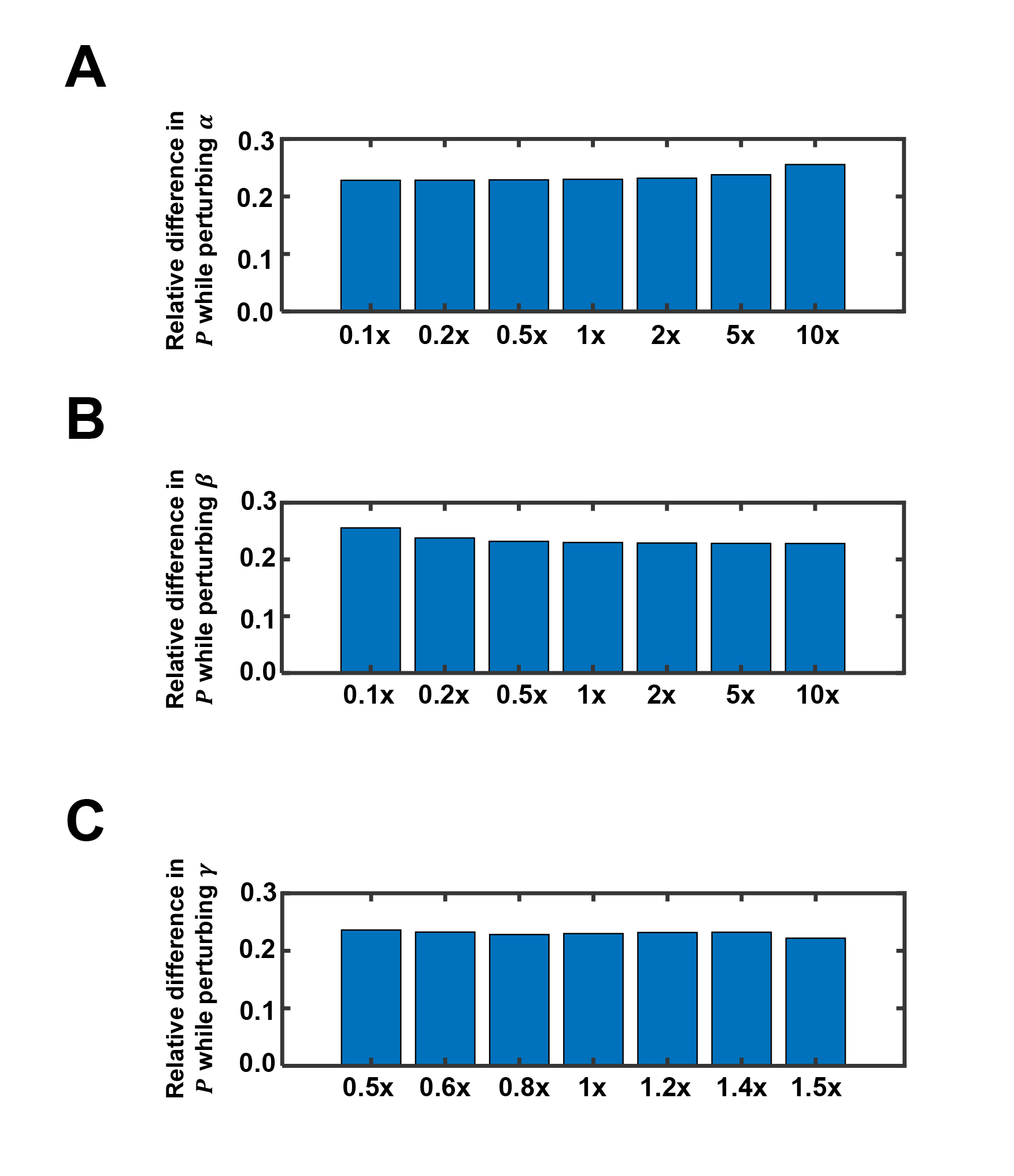

Supplement: S21 Fig — The relative difference in immunopathology between that corresponding to the population parameters estimated from mild and severe patient cohorts (see S8 Text), for different values of (A) α, (B) β, and (C) γ. α and β were varied from 0.1x to 10x of their default values, as indicated, whereas γ was varied from 0.5x to 1.5x of its default value. (TIF) [file ppat.1010630.s021.tif]
