# Supplementary material for: Modeling recapitulates the heterogeneous outcomes of SARS-CoV-2 infection and quantifies the differences in the innate immune and CD8 T-cell responses between patients experiencing mild and severe symptoms
Source: PLoS Pathog. 2022 Jun 27;18(6):e1010630. doi: 10.1371/journal.ppat.1010630 (PMC9269964; doi:10.1371/journal.ppat.1010630)
Supplement: S1 Text — (DOCX) [file ppat.1010630.s035.docx]

**S1 Text: Alternative metrics of immunopathology**

We considered the following alternative ways of calculating the immunopathology $P$ using model predictions of the tissue damage $D$:

Metric I: All the peaks of $D$ are found and summed to yield $P$. This would be a marker of the maximum instantaneous tissue damage suffered.

Metric II: Metric I is normalised by its value for the population parameters of mild patients.

Metric III: The half maximal level of the highest peak in $D$ is set as a threshold. The AUC of $D$ from the time it rises above and falls below the threshold is defined as $P$. This represents the overall tissue damage suffered.

Metric IV: Metric III is normalised by its value for the population parameters of mild patients.

Metric V: The half maximal level of the highest peak in $D$ for the population parameters of the mild patients is set as a threshold. The AUC of $D$ from the time it rises above and falls below the threshold is defined as $P$. This represents the overall tissue damage as above but is applicable across datasets that may have different extents of $D$.

Metric VI: Metric V is normalised by its value for the population parameters of mild patients.

We also assessed a variant of Metric VI, where we let the protective effect of the innate immune response dominate its pathological effect (by setting $\beta=0$ in evaluating $D$; see main text).

We quantified these metrics in our analysis of Fig 4. This is presented in S2 Fig. The trends across the metrics were similar. We thus choose metric VI for all our analysis because of its ready applicability across datasets and its normalization to mild patients which allows easy interpretability.
