# Supplementary material for: Modeling recapitulates the heterogeneous outcomes of SARS-CoV-2 infection and quantifies the differences in the innate immune and CD8 T-cell responses between patients experiencing mild and severe symptoms
Source: PLoS Pathog. 2022 Jun 27;18(6):e1010630. doi: 10.1371/journal.ppat.1010630 (PMC9269964; doi:10.1371/journal.ppat.1010630)
Supplement: S2 Text — (DOCX) [file ppat.1010630.s036.docx]

**S2 Text: Data collection and curation**

We collected the dates of exposures of all patients from the report by Böhmer et al. [1] (S1 Table). Some patients had multiple events of exposure on consecutive days; i.e., they encountered infected individuals on multiple occasions before they started showing symptoms themselves. We selected the first day of exposure as day 0, assuming that the viral transmission occurred on the first exposure. Further, we collected the dates of symptom onset for all patients and calculated the days post-infection associated with those dates (S2 Table). Next, we digitized the viral load data of these patients from the Figure 2 of the report by Wölfel et al. [2]. In the latter figure, viral load is plotted versus days post symptom onset. Combining the latter with the duration for symptom onset, we deduced the dates of the first viral load measurements, or diagnosis (S3 Table), allowing us to determine viral load as a function of the time from the first exposure, which is presented in our Fig 2. We thus collated data from day 0, i.e., the date of exposure, to day 15 into the infection. The earliest diagnostic data was available for patient 3, on day 4 post exposure. The latest diagnostic data was for patient 8, on day 12 post exposure. All dates provided in the tables are for the year 2020.

**References**

1. Bohmer MM, Buchholz U, Corman VM, Hoch M, Katz K, Marosevic DV, et al. Investigation of a COVID-19 outbreak in Germany resulting from a single travel-associated primary case: a case series. Lancet Infect Dis. 2020;20(8):920-8. doi: 10.1016/S1473-3099(20)30314-5.

2. Wolfel R, Corman VM, Guggemos W, Seilmaier M, Zange S, Muller MA, et al. Virological assessment of hospitalized patients with COVID-2019. Nature. 2020;581(7809):465-9. doi: 10.1038/s41586-020-2196-x.
