# Supplementary material for: Modeling recapitulates the heterogeneous outcomes of SARS-CoV-2 infection and quantifies the differences in the innate immune and CD8 T-cell responses between patients experiencing mild and severe symptoms
Source: PLoS Pathog. 2022 Jun 27;18(6):e1010630. doi: 10.1371/journal.ppat.1010630 (PMC9269964; doi:10.1371/journal.ppat.1010630)
Supplement: S3 Text — (DOCX) [file ppat.1010630.s037.docx]

**S3 Text: Detailed description of model selection**

The following candidate models were fit to the data and the corrected Akaike Information Criterion (AICc) and Bayesian Information Criterion (BICc) were determined (S7 Table). We present the equations and mention the differences from the model presented in the main text. We fit the alternate models to the data points shown in Fig 2 of the main text (S1 and S3 Tables) and estimated the AICc and BICc. (In particular, we fixed $k_{4}$ = 0 and the fixed and the random effect of $k_{1}$ as estimated in Table 2, in all following cases.)

**S3A. Model with saturating innate immune response and its priming of effector response**

$$\frac{dI}{dt}={[k}_{1}\left( 1-\varepsilon_{I}X \right)I\left( 1-\frac{I}{I_{max}} \right)-k_{2}IE]H(t-\tau)$$

$$\frac{dE}{dt}=[k_{3}\left( \frac{1}{k_{p}+I} \right)\left( 1+\varepsilon_{E}X \right)IE-k_{4}\left( \frac{1}{k_{e}+I} \right)IE]H(t-\tau)$$

$$\frac{dX}{dt}=[k_{5}I\left( 1-\frac{I}{k_{i}} \right)-k_{6}X]H(t-\tau)$$

Here, in addition to the terms in the model in the main text, the effect of the innate immune response, *X*, on CD8 T-cell stimulation, represented by the term $\varepsilon_{E}X$, and a maximum limit to the rate of induction of innate response via the carrying capacity $k_{i}$were introduced. All the other parameters and variables are the same as in the main text.

We rescaled the model using $k_{p}^{*}=k_{p}/I_{max}$, $k_{e}^{*}=k_{e}/I_{max}$, $k_{5}^{*}=k_{5}.I_{max}.\varepsilon_{I}$, $I^{*}=I/I_{max}$, $X^{*}=X.\varepsilon_{I}$, and $E^{*}=E.k_{2}$, ${\varepsilon_{E}^{*}=\varepsilon_{E}/\varepsilon}_{I}$, $k_{I}^{*}=k_{i}/I_{max}$, and obtained

$$\frac{dI^{*}}{dt}=\left[ k_{1}\left( 1-X^{*} \right)I^{*}\left( 1-I^{*} \right)-I^{*}E^{*} \right]H\left( t-\tau\right)$$

$$\frac{dE^{*}}{dt}=\left[ k_{3}\left( \frac{I^{*}E^{*}}{k_{p}^{*}+I^{*}} \right)(1+\varepsilon_{E}^{*}X^{*})-k_{4}\left( \frac{I^{*}E^{*}}{k_{e}^{*}+I^{*}} \right) \right]H\left( t-\tau\right)$$

$$\frac{dX^{*}}{dt}=\left[ k_{5}^{*}I^{*}(1-\frac{I^{*}}{k_{I}^{*}})-k_{6}X^{*} \right]H\left( t-\tau\right)$$

Best-fit parameter estimates are in S6A Table.

**S3B. Model with saturating immune response**

$$\frac{dI}{dt}={[k}_{1}\left( 1-\varepsilon_{I}X \right)I\left( 1-\frac{I}{I_{max}} \right)-k_{2}IE]H(t-\tau)$$

$$\frac{dE}{dt}=[k_{3}\left( \frac{1}{k_{p}+I} \right)IE-k_{4}\left( \frac{1}{k_{e}+I} \right)IE]H(t-\tau)$$

$$\frac{dX}{dt}=[k_{5}I\left( 1-\frac{I}{k_{i}} \right)-k_{6}X]H(t-\tau)$$

Here, the saturating innate immune response but not its effect on priming CD8 T-cells in model S3A was included.

We rescaled the model using $k_{p}^{*}=k_{p}/I_{max}$, $k_{e}^{*}=k_{e}/I_{max}$, $k_{5}^{*}=k_{5}.I_{max}.\varepsilon_{I}$, $I^{*}=I/I_{max}$, $X^{*}=X.\varepsilon_{I}$, and $E^{*}=E.k_{2}$, $k_{I}^{*}=k_{i}/I_{max}$, and obtained

$$\frac{dI^{*}}{dt}=\left[ k_{1}\left( 1-X^{*} \right)I^{*}\left( 1-I^{*} \right)-I^{*}E^{*} \right]H\left( t-\tau\right)$$

$$\frac{dE^{*}}{dt}=\left[ k_{3}\left( \frac{I^{*}E^{*}}{k_{p}^{*}+I^{*}} \right)-k_{4}\left( \frac{I^{*}E^{*}}{k_{e}^{*}+I^{*}} \right) \right]H\left( t-\tau\right)$$

$$\frac{dX^{*}}{dt}=\left[ k_{5}^{*}I^{*}(1-\frac{I^{*}}{k_{I}^{*}})-k_{6}X^{*} \right]H\left( t-\tau\right)$$

Best-fit parameter estimates are in S6B Table.

**S3C. Model without antiviral activity of innate immune response**

$$\frac{dI}{dt}={[k}_{1}I\left( 1-\frac{I}{I_{max}} \right)-k_{2}IE]H(t-\tau)$$

$$\frac{dE}{dt}=[k_{3}\left( \frac{1}{k_{p}+I} \right)\left( 1+\varepsilon_{E}X \right)IE-k_{4}\left( \frac{1}{k_{e}+I} \right)IE]H(t-\tau)$$

$$\frac{dX}{dt}=[k_{5}I\left( 1-\frac{I}{k_{i}} \right)-k_{6}X]H(t-\tau)$$

In this model, we removed the term $\left( 1-\varepsilon_{I}X \right)$ representing the innate immune response against the spread of the infection, from the dynamics of $I$ in model S3A.

We rescaled the model using $k_{p}^{*}=k_{p}/I_{max}$, $k_{e}^{*}=k_{e}/I_{max}$, $k_{5}^{*}=k_{5}.I_{max}.\varepsilon_{E}$, $I^{*}=I/I_{max}$, $X^{*}=X.\varepsilon_{E}$, and $E^{*}=E.k_{2}$, $k_{I}^{*}=k_{i}/I_{max}$, and obtained

$$\frac{dI^{*}}{dt}=\left[ k_{1}\left( 1-I^{*} \right)I^{*}-I^{*}E^{*} \right]H\left( t-\tau\right)$$

$$\frac{dE^{*}}{dt}=\left[ k_{3}\left( \frac{I^{*}E^{*}}{k_{p}^{*}+I^{*}} \right)(1+X^{*})-k_{4}\left( \frac{I^{*}E^{*}}{k_{e}^{*}+I^{*}} \right) \right]H\left( t-\tau\right)$$

$$\frac{dX^{*}}{dt}=\left[ k_{5}^{*}I^{*}(1-\frac{I^{*}}{k_{I}^{*}})-k_{6}X^{*} \right]H\left( t-\tau\right)$$

Best-fit parameter estimates are in S6C Table.

**S3D. Model without antiviral activity of CD8 T-cells**

$$\frac{dI}{dt}={[k}_{1}\left( 1-\varepsilon_{I}X \right)I\left( 1-\frac{I}{I_{max}} \right)]H(t-\tau)$$

$$\frac{dE}{dt}=[k_{3}\left( \frac{1}{k_{p}+I} \right)\left( 1+\varepsilon_{E}X \right)IE-k_{4}\left( \frac{1}{k_{e}+I} \right)IE]H(t-\tau)$$

$$\frac{dX}{dt}=[k_{5}I\left( 1-\frac{I}{k_{i}} \right)-k_{6}X]H(t-\tau)$$

Here, we removed the term $k_{2}IE$, which represents the CD8 T-cell mediated clearance of the infected cells from model S3A.

We rescaled the model using $k_{p}^{*}=k_{p}/I_{max}$, $k_{e}^{*}=k_{e}/I_{max}$, $k_{5}^{*}=k_{5}.I_{max}.\varepsilon_{I}$, $I^{*}=I/I_{max}$, $X^{*}=X.\varepsilon_{I}$, ${\varepsilon_{E}^{*}=\varepsilon_{E}/\varepsilon}_{I}$, $k_{I}^{*}=k_{i}/I_{max}$, and obtained

$$\frac{dI^{*}}{dt}=\left[ k_{1}\left( 1-X^{*} \right)I^{*}\left( 1-I^{*} \right) \right]H\left( t-\tau\right)$$

$$\frac{dE}{dt}=\left[ k_{3}\left( \frac{I^{*}E}{k_{p}^{*}+I^{*}} \right)(1+\varepsilon_{E}^{*}X^{*})-k_{4}\left( \frac{I^{*}E}{k_{e}^{*}+I^{*}} \right) \right]H\left( t-\tau\right)$$

$$\frac{dX^{*}}{dt}=\left[ k_{5}^{*}I^{*}(1-\frac{I^{*}}{k_{I}^{*}})-k_{6}X^{*} \right]H\left( t-\tau\right)$$

Best-fit parameter estimates are in S6D Table.

**S3E. Main model**

$$\frac{dI}{dt}={[k}_{1}\left( 1-\varepsilon_{I}X \right)I\left( 1-\frac{I}{I_{max}} \right)-k_{2}IE]H(t-\tau)$$

$$\frac{dE}{dt}=[k_{3}\left( \frac{1}{k_{p}+I} \right)IE-k_{4}\left( \frac{1}{k_{e}+I} \right)IE]H(t-\tau)$$

$$\frac{dX}{dt}=[k_{5}I-k_{6}X]H(t-\tau)$$

This is the model in the main text. Its rescaling and fitting are described in the main text.

**S3F. Model with innate immune response priming the effector response**

$$\frac{dI}{dt}={[k}_{1}\left( 1-\varepsilon_{I}X \right)I\left( 1-\frac{I}{I_{max}} \right)-k_{2}IE]H(t-\tau)$$

$$\frac{dE}{dt}=[k_{3}\left( \frac{1}{k_{p}+I} \right)\left( 1+\varepsilon_{E}X \right)IE-k_{4}\left( \frac{1}{k_{e}+I} \right)IE]H(t-\tau)$$

$$\frac{dX}{dt}=[k_{5}I-k_{6}X]H(t-\tau)$$

Here, the effect of the innate immune response on priming CD8 T-cells but not the saturating limit to the innate immune response in model S3A was included.

We rescaled the model using $k_{p}^{*}=k_{p}/I_{max}$, $k_{e}^{*}=k_{e}/I_{max}$, $k_{5}^{*}=k_{5}.I_{max}.\varepsilon_{I}$, $I^{*}=I/I_{max}$, $X^{*}=X.\varepsilon_{I}$, and $E^{*}=E.k_{2}$, ${\varepsilon_{E}^{*}=\varepsilon_{E}/\varepsilon}_{I}$, and obtained

$$\frac{dI^{*}}{dt}=\left[ k_{1}\left( 1-X^{*} \right)I^{*}\left( 1-I^{*} \right)-I^{*}E^{*} \right]H\left( t-\tau\right)$$

$$\frac{dE^{*}}{dt}=\left[ k_{3}\left( \frac{I^{*}E^{*}}{k_{p}^{*}+I^{*}} \right)(1+\varepsilon_{E}^{*}X^{*})-k_{4}\left( \frac{I^{*}E^{*}}{k_{e}^{*}+I^{*}} \right) \right]H\left( t-\tau\right)$$

$$\frac{dX^{*}}{dt}=\left[ k_{5}^{*}I^{*}-k_{6}X^{*} \right]H\left( t-\tau\right)$$

Best-fit parameter estimates are in S6E Table.

**S3G. Model with effector response dependent on rate of antigen increase**

$$\frac{dI}{dt}={[k}_{1}\left( 1-\varepsilon_{I}X \right)I\left( 1-\frac{I}{I_{max}} \right)-k_{2}IE]H(t-\tau)$$

$$\frac{dE}{dt}=[k_{3}\left( \frac{k_{1}I}{k_{p}+k_{1}I} \right)E-k_{4}\left( \frac{1}{k_{e}+I} \right)IE]H(t-\tau)$$

$$\frac{dX}{dt}=[k_{5}I-k_{6}X]H(t-\tau)$$

Here, the proliferation rate of effector cells is dependent on the rate of antigen increase, $k_{1}I$, rather than the level of antigen $I$. This model, however, was structurally identical to the main model (S3E), which is evident upon rearranging the first term on the right hand side of the equation for $E$ as $k_{3}\left( \frac{I}{k_{p}/k_{1}+I} \right)E$. Because $k_{p}$ was not known *a priori*, this model was indistinguishable from the main model using fits to the available data. The dependence of effector cell proliferation on the rate of antigen increase has been recognized previously [1-3]. The above model could be used to evaluate its role in SARS-CoV-2 infection once independent data on $k_{p}$ becomes available.

**References**

1. Grossman Z, Paul WE. Dynamic tuning of lymphocytes: physiological basis, mechanisms, and function. Annu Rev Immunol. 2015;33:677-713. doi: 10.1146/annurev-immunol-032712-100027.

2. Sontag ED. A dynamic model of immune responses to antigen presentation predicts different regions of tumor or pathogen elimination. Cell syst. 2017;4(2):231-41. e11. doi: 10.1016/j.cels.2016.12.003.

3. Cirelli KM, Carnathan DG, Nogal B, Martin JT, Rodriguez OL, Upadhyay AA, et al. Slow delivery immunization enhances HIV neutralizing antibody and germinal center responses via modulation of immunodominance. Cell. 2019;177(5):1153-71. e28. doi: 10.1016/j.cell.2019.04.012.
