# Supplementary material for: Modeling recapitulates the heterogeneous outcomes of SARS-CoV-2 infection and quantifies the differences in the innate immune and CD8 T-cell responses between patients experiencing mild and severe symptoms
Source: PLoS Pathog. 2022 Jun 27;18(6):e1010630. doi: 10.1371/journal.ppat.1010630 (PMC9269964; doi:10.1371/journal.ppat.1010630)
Supplement: S4 Text — (DOCX) [file ppat.1010630.s038.docx]

**S4 Text:** **Clearance is not stable in our model without CD8 T-cell response**

The non-dimensionalized model Eqs 5-7 in the absence of CD8 T-cells become:

$$\frac{dI}{dt}=k_{1}I\left( 1-X \right)\left( 1-I \right)$$

$$\frac{dX}{dt}=k_{5}I-k_{6}X$$

The asterisks are omitted for ease of notation. The terms and variables are defined in the main text. We solved the equations for steady state and obtained the following fixed points:

1. $I=0, X=0$
2. $I=\frac{k_{6}}{k_{5}},X=1$
3. $I=1, X=\frac{k_{5}}{k_{6}}$

Of these, fixed point 1 represents the clearance of infection. Its stability depends on the signs of the eigenvalues of the Jacobian matrix, *J*, of the model equations evaluated at the fixed point:

$$J=\left( \begin{matrix} \frac{\partial\left( \frac{dI}{dt} \right)}{\partial I} & \frac{\partial\left( \frac{dI}{dt} \right)}{\partial X} \\ \frac{\partial\left( \frac{dX}{dt} \right)}{\partial I} & \frac{\partial\left( \frac{dX}{dt} \right)}{\partial X} \end{matrix} \right)_{I,X}$$

We populated the Jacobian matrix and computed the eigenvalues.

For fixed point 1, the eigenvalues were $-k_{6}$ and $k_{1}$. The latter eigenvalue ($k_{1}$) is always positive. Hence, fixed point 1 is not stable. We concluded, therefore, that clearance is not possible in our model in the absence of CD8 T-cells.

Evidence of the importance of CD8 T-cells in the clearance of primary SARS-CoV-2 infection is growing, as reviewed recently [1]. For instance, whereas SARS-CoV-2-infected mice devoid of B cells could clear the virus, depletion of CD8+ cells from these mice led to an approximately hundred-fold increase in the viral load at day 14 post-infection compared to the control [2]. Future studies may test more directly the essentiality of CD8 T-cells to the clearance of SARS-CoV-2 infection.

**References**

1. Moss P. The T cell immune response against SARS-CoV-2. Nat. Immunol. 2022:1-8. doi: 10.1038/s41590-021-01122-w.

2. Israelow B, Mao T, Klein J, Song E, Menasche B, Omer SB, et al. Adaptive immune determinants of viral clearance and protection in mouse models of SARS-CoV-2. Sci. Immunol. 2021;6(64):eabl4509. doi: 10.1126/sciimmunol.abl4509
