# Supplementary material for: Modeling recapitulates the heterogeneous outcomes of SARS-CoV-2 infection and quantifies the differences in the innate immune and CD8 T-cell responses between patients experiencing mild and severe symptoms
Source: PLoS Pathog. 2022 Jun 27;18(6):e1010630. doi: 10.1371/journal.ppat.1010630 (PMC9269964; doi:10.1371/journal.ppat.1010630)
Supplement: S5 Text — (DOCX) [file ppat.1010630.s039.docx]

**S5 Text. Fixed points and their linear stability analysis**

We reproduce the non-dimensionalized model Eqs 5-7 for convenience below. We ignore the equation for *D* as it is decoupled from the rest.

$$\frac{dI}{dt}=k_{1}I\left( 1-X \right)\left( 1-I \right)-IE$$

$$\frac{dE}{dt}=\frac{k_{3}}{k_{p}+I}IE-\frac{k_{4}}{k_{e}+I}IE$$

$$\frac{dX}{dt}=k_{5}I-k_{6}X$$

The terms and variables are defined in the main text. The asterisks are omitted for convenience. We solved the equations for steady state and obtained the following fixed points:

1. $I=0, E \geq0, X=0$
2. $I=\frac{k_{6}}{k_{5}}, E=0, X=1$
3. $I=1, E=0, X=\frac{k_{5}}{k_{6}}$
4. $I=\frac{k_{e}k_{3}-k_{p}k_{4}}{k_{4}-k_{3}}, E=k_{1}\left( 1-\frac{k_{5}}{k_{6}}I \right)\left( 1-I \right),X=\frac{k_{5}}{k_{6}}I$

The stability of the fixed points depends on the signs of eigenvalues of the Jacobian matrix, *J*, of the model equations evaluated at the fixed points:

$$J=\left( \begin{matrix} \frac{\partial\left( \frac{dI}{dt} \right)}{\partial I} & \frac{\partial\left( \frac{dI}{dt} \right)}{\partial E} & \frac{\partial\left( \frac{dI}{dt} \right)}{\partial X} \\ \frac{\partial\left( \frac{dE}{dt} \right)}{\partial I} & \frac{\partial\left( \frac{dE}{dt} \right)}{\partial E} & \frac{\partial\left( \frac{dE}{dt} \right)}{\partial X} \\ \frac{\partial\left( \frac{dX}{dt} \right)}{\partial I} & \frac{\partial\left( \frac{dX}{dt} \right)}{\partial E} & \frac{\partial\left( \frac{dX}{dt} \right)}{\partial X} \end{matrix} \right)_{I,E,X}$$

We populated the Jacobian matrix and computed the eigenvalues. For fixed point 1, the eigenvalues were $-k_{6}$, $0$, and $k_{1}-E$. Note that the fixed point is really a line of fixed points on the E-axis (see S10 Fig). It follows from the eigenvalues that the fixed points with $E<k_{1}$ act as saddle points while those with $E>k_{1}$ act as marginally stable (attracting) fixed points. The latter represent clearance. When the initial value of $E=0$, there is a stable steady state in the $E=0$ plane (Fig 3B).

For fixed point 2, the eigenvalues were:

1. $\left( \frac{k_{3}}{k_{p}+\frac{k_{6}}{k_{5}}}-\frac{k_{4}}{k_{e}+\frac{k_{6}}{k_{5}}} \right)\frac{k_{6}}{k_{5}}$
2. $k_{6}\left( -1+\sqrt{1+\frac{4k_{1}}{k_{5}}\left( 1-\frac{k_{5}}{k_{6}} \right)} \right)$
3. $k_{6}\left( -1-\sqrt{1+\frac{4k_{1}}{k_{5}}\left( 1-\frac{k_{5}}{k_{6}} \right)} \right)$

Depending on the parameters, which we discuss below, this fixed point could be stable or unstable.

For fixed point 3, the eigenvalues were

1. $-k_{6}$
2. $\frac{k_{3}}{k_{p}+1}-\frac{k_{4}}{k_{e}+1}$
3. $k_{1}\left( \frac{k_{5}}{k_{6}}-1 \right)$

Here, one eigenvalue (3^rd^) is always positive because $k_{5}>k_{6}$ for our system. Hence, it can never be a stable fixed point.

For fixed point 4, the calculation of the eigenvalues explicitly was not required as it was always unstable for positive values of *E*. Using the model equations before non-dimensionalization (Eqs. 1-3) for clarity, the eigenvalues must satisfy the characteristic equation $\lambda^{3}-\lambda^{2}\left( J_{11}-k_{6} \right)-\lambda\left( J_{11}k_{6}+J_{12}J_{21}+J_{13}k_{5} \right)-J_{12}J_{21}k_{6}=0$, where $J_{ij}$ is the *ij*^th^ term of the Jacobian. Denoting the three roots of the equation as $\lambda_{1}$, $\lambda_{2}$, and $\lambda_{3}$, it follows that the product of the roots, $\lambda_{1}{\lambda_{2}\lambda_{3}=J}_{12}J_{21}k_{6}$. If fixed point 4 were stable, then its eigenvalues would have negative real parts. Two scenarios are then possible: Either all the roots are negative and real or one of the roots is real and negative and the other are complex conjugates (whose product is always positive). In either case, the product of the roots would be negative. We know that $J_{12}=-k_{2}I<0$ because $I>0$ when $k_{P}\ll k_{e}$ and $k_{3}<k_{4}$. Further, $J_{21}= \frac{k_{4}EI(k_{P}-k_{e})}{{{(k}_{e}+I)}^{2}{(k}_{P}+I)}<0$ when $E>0$. This implies that $J_{12}J_{21}k_{6}>0$. In other words, fixed point 4 cannot be stable for any $E>0$.

Fixed point 2 represented persistent infection where the CD8 T-cell response was suppressed by the virus. Importantly, its stability was independent of the stability of the clearance state. Thus, parameter combinations could be identified where clearance and persistence were both stable. The system could thus exhibit bistability. Such bistability has been proposed earlier for other viral infections, including HIV [1-4]. The outcomes realized would then depend on initial conditions. By analyzing parameter regimes, we identified combinations when fixed points 2 could be stable. The regimes are depicted in S9 Fig.

For the best-fit population parameter estimates (Table 2), the system admitted a single stable steady state, fixed point 1, indicating that clearance was the only outcome realized (S10A Fig). The path to clearance, however, could vary widely and depend on the initial CD8 T-cell population. A large initial effector pool could facilitate rapid clearance, in agreement with observations of such clearance facilitated by cross-reactive effector T-cells [5, 6].

If the parameters are varied in a way that the strength of the immune system decreases, bistability is introduced into the system. Fixed point 2 would then become stable depending on the parameter regimes. In the latter regimes, depending on the initial conditions, trajectories can then either go towards clearance or persistence (S10B Fig). In our predictions the latter trajectories were associated with large infected cell numbers and high inflammatory cytokine levels. Such trajectories may end prematurely due to mortality because of high immunopathology. We note that trajectories heading towards fixed point 1 may also be similarly terminated if initial conditions are such that excessive immunopathology results.

**References**

1. Baral S, Antia R, Dixit NM. A dynamical motif comprising the interactions between antigens and CD8 T cells may underlie the outcomes of viral infections. Proc Natl Acad Sci U S A. 2019;116(35):17393-8. doi: 10.1073/pnas.1902178116.

2. Raja R, Baral S, Dixit NM. Interferon at the cellular, individual, and population level in hepatitis C virus infection: Its role in the interferon‐free treatment era. Immunol. Rev. 2018;285(1):55-71. doi: 10.1111/imr.12689.

3. Conway JM, Perelson AS. Post-treatment control of HIV infection. Proc Natl Acad Sci U S A. 2015;112(17):5467-72. doi: 10.1073/pnas.1419162112.

4. Desikan R, Raja R, Dixit NM. Early exposure to broadly neutralizing antibodies may trigger a dynamical switch from progressive disease to lasting control of SHIV infection. PLoS Comput. Biol. 2020;16(8):e1008064. doi: 10.1371/journal.pcbi.1008064.

5. Lineburg KE, Grant EJ, Swaminathan S, Chatzileontiadou DS, Szeto C, Sloane H, et al. CD8+ T cells specific for an immunodominant SARS-CoV-2 nucleocapsid epitope cross-react with selective seasonal coronaviruses. Immunity. 2021;54(5):1055-65. e5. doi: 10.1016/j.immuni.2021.04.006.

6. Tan AT, Linster M, Tan CW, Le Bert N, Chia WN, Kunasegaran K, et al. Early induction of functional SARS-CoV-2-specific T cells associates with rapid viral clearance and mild disease in COVID-19 patients. Cell Rep. 2021;34(6):108728. doi: 10.1016/j.celrep.2021.108728.
