# Supplementary material for: Modeling recapitulates the heterogeneous outcomes of SARS-CoV-2 infection and quantifies the differences in the innate immune and CD8 T-cell responses between patients experiencing mild and severe symptoms
Source: PLoS Pathog. 2022 Jun 27;18(6):e1010630. doi: 10.1371/journal.ppat.1010630 (PMC9269964; doi:10.1371/journal.ppat.1010630)
Supplement: S6 Text — (DOCX) [file ppat.1010630.s040.docx]

**S6 Text. Effect of varying viral inoculum size**

To check the effects of the size of viral inoculum on the trajectories of the infection, we simulated our model with the estimated population parameters and varied the initial number of infected cells from 1 to 100. Apart from the timing of the peak of infection, the dynamics played out similarly in all the cases (S11A Fig). The peak viral load and the immunopathology suffered were comparable in all cases (S11B Fig). This is consistent with studies on macaques where infection with different inoculum sizes led to comparable disease outcomes [1]. Moreover, smaller the inoculum, longer was the onset time of the rise in viral load [1].

**Reference**

1. Chandrashekar A, Liu J, Martinot AJ, McMahan K, Mercado NB, Peter L, et al. SARS-CoV-2 infection protects against rechallenge in rhesus macaques. Science. 2020;369(6505):812-7. doi: 10.1126/science.abc4776.
