# Supplementary material for: Modeling recapitulates the heterogeneous outcomes of SARS-CoV-2 infection and quantifies the differences in the innate immune and CD8 T-cell responses between patients experiencing mild and severe symptoms
Source: PLoS Pathog. 2022 Jun 27;18(6):e1010630. doi: 10.1371/journal.ppat.1010630 (PMC9269964; doi:10.1371/journal.ppat.1010630)
Supplement: S7 Text — (DOCX) [file ppat.1010630.s041.docx]

**S7 Text. Model with an alternative formalism for CD8 T-cell exhaustion**

Following previous studies [1-4], we considered a model that allowed exhaustion to develop with the accumulation of antigenic stimulation rather than the instantaneous level of antigen. The model equations were thus:

$$\frac{dI}{dt}=k_{1}\left( 1-\varepsilon_{I}X \right)I\left( 1-\frac{I}{I_{max}} \right)-k_{2}IE$$

$$\frac{dE}{dt}=k_{3}\left( \frac{IE}{k_{p}+I} \right)-k_{4}\left( \frac{Q^{n}E}{q_{c}^{n}+Q^{n}} \right)$$

$$\frac{dX}{dt}=k_{5}I-k_{6}X$$

$$\frac{dQ}{dt}=\frac{I}{\phi+I}-d_{q}Q$$

Here, $Q$ marks the level of exhaustion, which increases as the integral of the level of antigenic stimulation $I$, and decreases naturally in the absence of stimulation with the rate constant $d_{q}$. The stimulation has a half-maximal value $\phi$. The effector function, $E$, decreases with $Q$ in a saturable manner with half-maximal level $q_{c}$ and Hill coefficient $n$. The remaining terms in the model are the same as in the main text. To fit the model to data, we rescaled the terms and introduced Heaviside functions, to account for the delay in viral propagation post infection, and obtained:

$$\frac{dI^{*}}{dt}=\left[ k_{1}\left( 1-X^{*} \right)I^{*}\left( 1-I^{*} \right)-I^{*}E^{*} \right]H\left( t-\tau\right)$$

$$\frac{dE^{*}}{dt}=\left[ k_{3}\left( \frac{I^{*}E^{*}}{k_{p}^{*}+I^{*}} \right)-k_{4}\left( \frac{Q^{n}E^{*}}{q_{c}^{n}+Q^{n}} \right) \right]H\left( t-\tau\right)$$

$$\frac{dX^{*}}{dt}=\left[ k_{5}^{*}I^{*}-k_{6}X^{*} \right]H\left( t-\tau\right)$$

$$\frac{dQ}{dt}=[\frac{I^{*}}{\phi^{*}+I^{*}}-d_{q}Q]H\left( t-\tau\right)$$

The model had many more parameters than our main model. For simplification, we set $d_{q}=0$ and $n=1$ and fixed $\phi^{*}=0.01$ following previous estimates [1]. The model offered good fits to the data (S18 Fig) and yielded estimates of parameters (S12 Table). The model, however, displayed a poorer BICc value (298.4) compared to the main model (279).

**References**

1. Baral S, Antia R, Dixit NM. A dynamical motif comprising the interactions between antigens and CD8 T cells may underlie the outcomes of viral infections. Proc Natl Acad Sci U S A. 2019;116(35):17393-8. doi: 10.1073/pnas.1902178116.

2. Conway JM, Perelson AS. Post-treatment control of HIV infection. Proc Natl Acad Sci U S A. 2015;112(17):5467-72. doi: 10.1073/pnas.1419162112.

3. Desikan R, Raja R, Dixit NM. Early exposure to broadly neutralizing antibodies may trigger a dynamical switch from progressive disease to lasting control of SHIV infection. PLoS Comput Biol. 2020;16(8):e1008064. doi: 10.1371/journal.pcbi.1008064.

4. Johnson PL, Kochin BF, McAfee MS, Stromnes IM, Regoes RR, Ahmed R, et al. Vaccination alters the balance between protective immunity, exhaustion, escape, and death in chronic infections. J Virol. 2011;85(11):5565-70. doi: 10.1128/JVI.00166-11.
