# Supplementary material for: Modeling recapitulates the heterogeneous outcomes of SARS-CoV-2 infection and quantifies the differences in the innate immune and CD8 T-cell responses between patients experiencing mild and severe symptoms
Source: PLoS Pathog. 2022 Jun 27;18(6):e1010630. doi: 10.1371/journal.ppat.1010630 (PMC9269964; doi:10.1371/journal.ppat.1010630)
Supplement: S8 Text — (DOCX) [file ppat.1010630.s042.docx]

**S8 Text. Effect of variations in parameters associated with immunopathology**

To test if our results are sensitive to variations in $\alpha, \beta$ or $\gamma$, we estimated the difference between the immunopathology calculated from the parameters (i.e., fixed effects) derived from fitting the clinical datasets of mild and severe patients respectively (Fig 5, tables 4 and 5), for different values of $\alpha, \beta$ or $\gamma$. While the absolute value of the immunopathology may change, if the relative values for the two cases remained robust, it would imply that our results are not sensitive to the choice of values of $\alpha, \beta$ or $\gamma$. We varied $\alpha$ and $\beta$10-fold up and down and calculated the relative difference in immunopathology ($P$) for each parameter set as $\frac{P_{severe}-P_{mild}}{P_{severe}}$. For $\gamma$, i.e., the rate of recovery from the effects of immunopathology, we varied the parameter value 50% up and down, since increasing it ten-fold above the nominal value of 0.5 was unrealistic. The default value of the relative difference was 0.147 (when $\alpha, \beta,\gamma$ had values 1e+4, 2e+4 and 0.5, respectively). Parameters were varied individually (S21 Fig), and we did not observe any remarkable alteration in the relative difference in immunopathology. Our results were thus robust to variations in $\alpha, \beta$ and $\gamma$.
